# digIS: towards detecting distant and putative novel insertion sequence elements in prokaryotic genomes

**DOI:** 10.1186/s12859-021-04177-6

**Published:** 2021-05-20

**Authors:** Janka Puterová, Tomáš Martínek

**Affiliations:** IT4Innovations Centre of Excellence, Faculty of Information Technology, Brno University of Technology, Bozetechova 2, 612 66 Brno, Czechia

**Keywords:** IS elements, Mobile element, Profile HMM, Prokaryotic genomes, Genome annotation

## Abstract

**Background:**

The insertion sequence elements (IS elements) represent the smallest and the most abundant mobile elements in prokaryotic genomes. It has been shown that they play a significant role in genome organization and evolution. To better understand their function in the host genome, it is desirable to have an effective detection and annotation tool. This need becomes even more crucial when considering rapid-growing genomic and metagenomic data. The existing tools for IS elements detection and annotation are usually based on comparing sequence similarity with a database of known IS families. Thus, they have limited ability to discover distant and putative novel IS elements.

**Results:**

In this paper, we present *digIS*, a software tool based on profile hidden Markov models assembled from catalytic domains of transposases. It shows a very good performance in detecting known IS elements when tested on datasets with manually curated annotation. The main contribution of *digIS* is in its ability to detect distant and putative novel IS elements while maintaining a moderate level of false positives. In this category it outperforms existing tools, especially when tested on large datasets of archaeal and bacterial genomes.

**Conclusion:**

We provide *digIS*, a software tool using a novel approach based on manually curated profile hidden Markov models, which is able to detect distant and putative novel IS elements. Although *digIS* can find known IS elements as well, we expect it to be used primarily by scientists interested in finding novel IS elements. The tool is available at https://github.com/janka2012/digIS.

**Supplementary Information:**

The online version contains supplementary material available at 10.1186/s12859-021-04177-6.

## Background

Insertion sequence elements (IS elements) are the smallest and most abundant autonomous transposable elements in prokaryotic genomes, usually ranging from 700 bp to 3 kbp. However, there are exceptions, and some IS families (Tn3) can contain elements having a length greater than 5 kbp. ISs are widespread in prokaryotic genomes and may occur in high copy numbers. They play an essential role in genome evolution, structure, and host-genome adaptability. Due to their movement ability, IS elements represent mutagenic agents and can: cause modulation of expression of neighboring genes, affect virulence, change xenobiotic or antimicrobial resistance, or modulate metabolic activities. Detailed information on IS element function in host genomes can be found in recent reviews [1, 2].

Typically, IS elements consist of one or two open reading frames (ORFs) encoding a transposase (Tpase), a product necessary for transposition within a particular genome or horizontally between genomes (in plasmids). They are flanked by short terminal inverted repeats (IRs) and direct repeats (DRs). Transposases occurring in IS elements include five groups named after amino acid residues located at their conserved catalytic domain that catalyzes the transposition: DDE, DEDD, HUH, Tyrosine (Y), and Serine (S). IS elements with DDE transposase are the most abundant, and their conserved catalytic domain has a typical secondary structure $\beta 1-\beta 2-\beta 3-\alpha 1-\beta 4-\alpha 2/3-\beta 5-\alpha 4-\alpha 5/6$. Classification of IS elements into families is based mainly on Tpase structure, but other features such as IRs and DRs are also considered. Up to now, 29 IS families have been identified [1].

ISfinder [3] is a human-curated database and the most comprehensive source of known IS elements at present. Currently, the database contains more than 5000 entries and is updated regularly. As an extension of the ISfinder database, the authors implemented an ISbrowser interface [4] for visualization of IS elements inside genomes, and they prepared a benchmark dataset, consisting of 118 manually annotated prokaryotic genomes (as of November 2017), that is often used for assessment of IS detection tools performance. Another data source focused on mobile genetic elements, including manually annotated insertion sequences, is ACLAME database [5]. Unfortunately, this database has not been updated since 2009.

Even though the databases of known IS elements are growing, we are probably far from having a complete knowledge of all IS families and their structures. Therefore, for a better understanding of the IS elements function and their role in genome evolution, it is desirable to have an effective tool capable of not only annotating known families but also detecting new ones. This need becomes even more crucial when considering rapid-growing genomic and metagenomic data.

At present, there are several tools available for the detection of IS elements in prokaryotic genomes. Some of them are designed for searching in raw sequenced data (ISQuest [6], ISMapper [7], ISseeker [8], panISa [9]), and the others require assembled sequences (IScan [10], ISsaga [11], OASIS [12], ISEScan [13], TnpPred [14]). Almost all tools utilize a homology-based approach and are dependent on a source of known IS elements (they use a reference database either for verifying their results or for building searching profiles). Only the panISa tool detects IS elements solely based on structural features, such as an alignment of DR regions, and does not require a reference database.

Homology-based methods can be further divided into two main categories: (1) sequence-based and (2) profile-based methods. The first category is represented by tools IScan, OASIS, ISQuest, and ISseeker, which utilize the ISfinder database as a reference library in combination with BLAST software [15] to find close homologs. These tools are often used in annotation pipelines, where outputs with a high level of confidence are required.

The latter category includes ISsaga, TnpPred, and ISEScan. They take advantage of interpolated Markov models or profile hidden Markov models (pHMMs), which provide a more sensitive search, and detect remote homology sequences. ISsaga utilizes GLIMMER [16] and detects ORFs of IS elements or their fragments using an optimized interpolated Markov model built from the ISfinder database. TnpPred is focused on transposases detection (not full-length IS elements) and provides pHMMs for 19 of 29 IS families only. ISEScan uses 621 pHMMs built automatically from Tpases in the ACLAME database, but 355 of them are made up of one sequence only. Based on the configuration, ISEScan searches for whole Tpases or allow the presence of fragments.

Both sequence-based and profile-based tools can find new members of existing IS families, as they usually share significant sequence similarity either at the DNA or Tpase/ORF level. Profile-based methods are able to find remote members with lower similarity, which can represent hitherto undiscovered families—distant putative novel IS elements. However, the reliable identification of new IS families and their members is still challenging even for existing profile-based tools. It is mainly due to the Tpase structure, which comprises of several, often variable, domains. A search for the whole Tpase (ISEScan) is quite specific and unable to uncover novel IS elements with a distinct Tpase structure. On the other hand, allowing for fragments (ISEScan, ISsaga, and TnpPred) may result in many hits having significant similarity to a specific part of a completely different protein (i.e., false positives in terms of tool evaluation).

In this paper, we address the aforementioned challenge using a novel approach to detecting distant members of known IS families and putative novel IS elements. The fundamental idea is to search for the most conserved part of Tpase—the catalytic domain. The search is based on manually curated pHMMs with noise cutoff thresholds. Utilizing this approach, we can detect both known and putative novel IS elements with a moderate level of false positives while maintaining high sensitivity. The proposed method is implemented as *digIS* software and released as open-source at https://github.com/janka2012/digIS. The installed tool, including all dependencies, is also available as a docker image at https://hub.docker.com/r/janka2012/digis.

## Implementation

*digIS* is a command-line tool developed in Python. It utilizes several external tools such as BLAST [15], HMMER [17], and Biopython library [18]. As an input, *digIS* accepts contigs in FASTA format. Optionally, the user can provide a GenBank annotation file for a given input sequence(s). This annotation is later used to improve the classification of identified IS elements (see “Output classification” section).

Firstly, we built a library of manually curated pHMMs, corresponding to Tpase catalytic domains of individual IS families. As a source of sequences, we used the ISfinder database, and for each pHMM, we identified the noise cutoff threshold.

Then, the *digIS* search pipeline operates in the following way:

1. The whole input nucleic acid sequence is translated into amino acid sequences (all six frames).
2. The translated sequences are searched using manually curated pHMMs.
3. Found hits, referred to as *seeds*, are filtered by domain bit score and e-value. Those that overlap or follow one another within a certain distance are merged.
4. Each seed is matched against the database of known IS elements (ISfinder) and its genomic positions are extended according to the best hit.
5. Extended seeds are filtered by noise cutoff score and length. Duplicates, corresponding to the same IS element, are removed.
6. Remaining extended seeds are classified based on sequence similarity and GenBank annotation (if available) to assess their quality.
7. Finally, the classified outputs are reported in the CSV and GFF3 format.

**Fig. 1:**
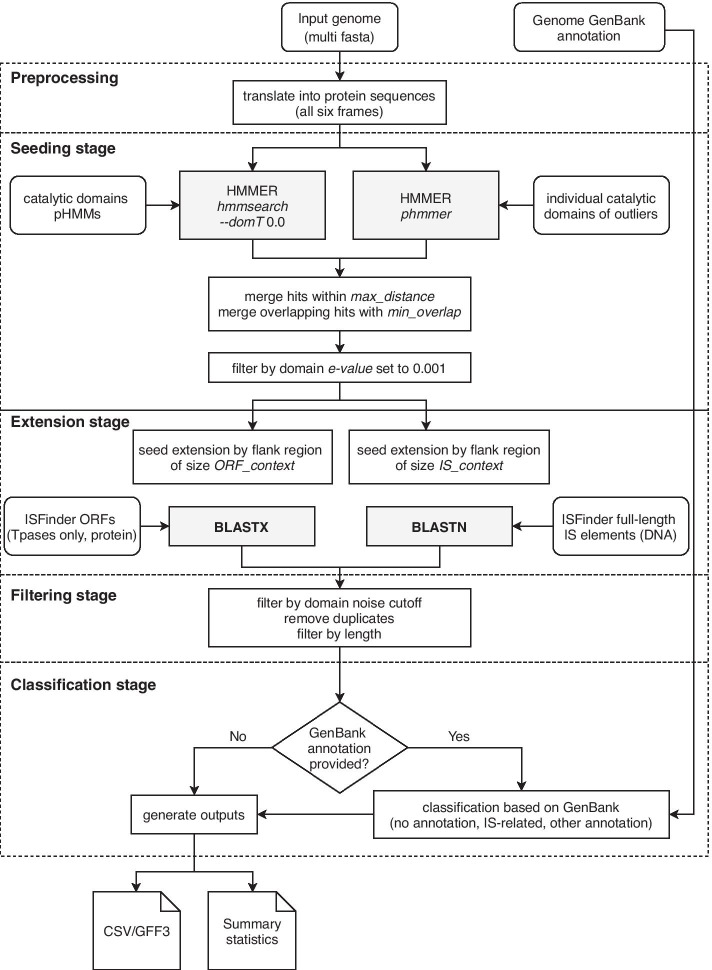
Workflow of *digIS*. digIS components and workflow, grey rectangles represent external tools, rounded rectangles represent input data, white rectangles represent digIS components

The overall *digIS* workflow is depicted in Fig. 1, and the individual steps are described in detail in the following sections.

### Building profile hidden Markov models for the transposase catalytic domain of individual IS families

Tpase sequences were obtained from the ISfinder database. For each IS family, the pHMM was created as follows: (1) the longest ORF sequence, representing Tpase and its catalytic domain, was chosen for each IS element^1^, (2) a multiple sequence alignment (MSA) for a set of Tpases belonging to the same family was created by Clustal Omega [19] and visualized using Jalview [20], (3) for each MSA, a protein secondary structure of the transposase was predicted using JPred4 [21] and used to determine the boundaries of the conserved catalytic core; the MSA was refined based on the positions of the catalytic residues (usually DDE), and the catalytic domain was manually cut using these determined boundaries, (4) such a manually modified MSA was used to construct resultant pHMM using *hmmbuild* from the HMMER package.

Since IS3, IS4, and IS5 families contain multiple subfamilies, a separate model was constructed for each of them. Moreover, IS5/IS5 and IS5/None subfamilies showed various sequence patterns (e.g., long insertions, deletions), and therefore several models were built for them concerning these patterns. MSAs with highlighted sequence groups used to construct these models are available in Additional files 1 and 2. For the ISNCY family, models were built for IS1202 and ISDol1 subfamilies only, since other subfamilies did not contain a sufficient amount of sequences. We required the models to be assembled from at least ten sequences to have a generalizing ability to find distant Tpases. Altogether, 50 pHMMs were constructed.

The remaining sequences of IS5 and ISNCY subfamilies representing outliers/distant sequences were cut with regard to the catalytic residues and secondary structure. They were used later as *individual* protein sequences in *phmmer* search. Overall, 70 outlier sequences were collected.

To eliminate false-positive hits reported by HMMER using pHMMs and still have the ability to detect distant and novel IS elements, a domain noise cutoff threshold—which represents a bit score of the highest-scoring known false positive—was determined for each pHMM as follow: First, a database of manually curated protein sequences from Archaea and Bacteria kingdoms was collected from SwissProt [22] and RefSeq [23] databases (records labeled as ‘REVIEWED’), resulting in 353051 and 232157 records (accessed on 11 March 2019), respectively. Setting this threshold is a common practice and is used, for example, in models stored in Pfam [24] database. Then, each pHMM was queried against this reference protein database employing *hmmsearch* with default settings. Finally, reported hits were sorted in a descending order based on the reported per-domain bit score and evaluated manually to estimate the bit score from which false positive hits were prevalent.

### Searching for IS elements in the input sequence

In the beginning, the whole input nucleic acid sequence is translated into amino acids (all six frames). Then, the search process operates in two steps:

1. *Seeding*: The input genome is scanned using pHMMs and *individual* sequences representing Tpase catalytic domains. Each occurrence with a satisfactory score is labeled as a seed.
2. *Extension*: The genomic position of seeds identified in the previous step are extended based on the similarity boundaries with Tpases and IS elements from the ISfinder database.

In the *Seeding* stage, *digIS* utilizes *hmmsearch* from the HMMER3 package to query pHMMs against the translated sequences with an enabled domain threshold (*–domT* argument) set to 0.0 to report domain hits with a non-negative bit score only. Afterwards, *digIS* employs *phmmer* to query *individual* protein sequences against the translated sequences. The resulting hits are post-processed and filtered by a domain conditional e-value set to 0.001. Next, neighboring records, detected by the same model within a certain distance (700 bp^2^) on the same strand, are merged. This approach allows insertions or variable segments inside catalytic domains that are typical for some Tpases [25]. Next, overlapping records found by different models are merged, since there exists a sequence similarity in the catalytic domain among different Tpases, or a putative novel catalytic domain might be composed of different parts of known domains.

Please note that *digIS* scans the whole input sequence, instead of just open reading frames (ORFs), to not omit some coding regions.

**Fig. 2:**
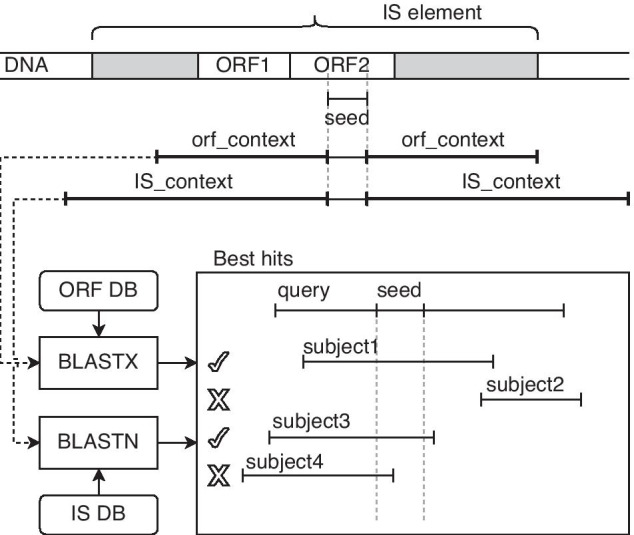
Seed extension process. The seed matches the catalytic domain of the putative IS element in the input genomic sequence. This seed is extended with upstream and downstream flank regions of orf_context and IS_context size, respectively, and is searched against the database of known Tpase/ORFs and IS elements, respectively. Only the best hits, including the whole original seed, are considered for extension. Position of the seed is changed (extended) according to the best hit

During the next stage (*Extension*), the genomic position of each seed is identified in the original nucleic acid sequence and extended with *context_orf* and *context_dna* (upstream and downstream flank regions of a length 1600 bp^3^, and 14000 bp^4^, respectively), see Fig. 2. Next, the extended seed is matched against sequences of known Tpases (ORF level) and IS elements (DNA level), extracted from the ISfinder database, using the BLASTX and BLASTN tools. Finally, the seed’s original position is adjusted (extended) according to the best BLAST hits’ positions.

As the output of the *Extension* stage, the *digIS* tool reports: (1) position at DNA level if the similarity with a known IS element was found using the BLASTN tool; or (2) position at the ORF level if the similarity with a known Tpase was found using the BLASTX tool; or (3) position of the catalytic domain otherwise found during the *Seeding* stage.

### Output filtering

To eliminate the number of reported false positives, *digIS* filters the hits with a score below the previously estimated noise cutoff threshold, and it removes duplicate records covering the same genomic region. Lastly, hits having less than 150 bp (50 aa) in length are filtered out.

### Output classification

To help the user assess the quality of found IS elements, each output hit is supplemented by information about sequence similarity with known IS elements and Tpases extracted from the ISfinder database. The similarity is calculated as a percentage of identity between the extended seed and a known IS element or Tpase sequence, measured according to the database item’s length.

In case the GenBank annotation is provided as an optional input^5^, the classification process is further extended, and each *digIS* hit is classified based on the overlap with GenBank annotation records into the three categories using following rules applied in the subsequent order:

- *IS-related*—hit overlaps with a GenBank record of type: (1) mobile element or mobile element type, (2) repeat region, coding sequence (CDS), gene, or miscellaneous feature annotated as transposase, resolvase, recombinase, recombination/resolution, insertion element, mobile element, transposon, transposable element, DDE, or the annotation contains a name of known IS family or subfamily [27, 28]. A hit classified into this category has high confidence to be a true IS element.
- *no annotation*—hit does not overlap with any GenBank record or overlaps with a record annotated as a hypothetical protein, predicted protein, unknown, or domain of the unknown function (DUF). The hit in this category can be seen as an unknown protein or protein, where the annotation pipeline did not achieve a sufficient level of confidence. Typically, distant or putative novel IS family members may belong to this category.
- *other annotation*—otherwise. The hit in this category is probably not an IS element, because it overlaps and shares significant similarity with a different protein.

Since the previous analysis of GenBank annotation revealed that some IS element transposases were misannotated as integrases [6, 12], we classify all hits annotated as integrases and at the same time having significant identity to a known IS element in the ISfinder database (at ORF or DNA level), as *IS-related* as well.

The latest version of the GenBank annotation was newly expanded to include fragments of IS elements marked as ’pseudo’ with the notation ’incomplete’ [26]. To preserve a conservative approach and high confidence, these records are ignored when classifying hits.

### *digIS* output files

The *digIS* tool generates the following output files: (1) a CSV and GFF3 file containing all found IS elements and their attributes such as sequence ID, genomic location, strand, accuracy, score, sequence similarities with known IS elements (at ORF and DNA level), and classification according to GenBank annotation (if provided); (2) a summary file containing numbers of IS elements per individual families, overall numbers of base pairs and a percentage of an input sequence occupied by IS elements. FASTA sequences of found IS elements can be extracted using the GFF3 file and BEDTools [29] (see instructions on the GitHub repository).

## Results

The performance of the *digIS* tool was evaluated on different datasets and compared with related tools. Specifically, we chose ISEScan (version 1.6), OASIS (version released 18th September 2012), and ISsaga (version with the last update on 20th January 2020). Other state-of-the-art tools were excluded for various reasons. ISMapper, ISseeker, ISQuest, and panISa are designed for IS elements detection in raw sequence reads. TnpPred is available online only, and it is limited to protein sequences with a maximum length of 5000 amino acids. Even though the TnpPred pHMMs are available for download, it is unclear what kind of parameters or filtration mechanisms should be used during the search. Finally, we excluded IScan, because we were not able to install it, including all necessary dependencies.

All tools were run with default or recommended settings. Additionally, ISEScan was executed with two settings: (1) default configuration with the *removeShortIS* option enabled, when IS elements shorter than 400 bp or single copy IS elements without perfect IRs are filtered out; and (2) with *removeShortIS* turned off when all hits are reported (hereinafter referred to as ISEScan–fragments).

We faced several issues when evaluating the tools. At first, the definition of a true positive hit was ambiguous as different tools reported different types of outputs. Some tools reported entire IS elements at the DNA level (ISEScan and OASIS) or their fragments (ISEScan–fragments). Other tools reported individual ORFs or fragments thereof (ISsaga), while the proposed *digIS* tool reported outputs at one of three levels (catalytic domain, ORF, or DNA). Moreover, for tools reporting ORFs or fragments, it is common that several hits correspond to the same IS element from the reference dataset.

**Fig. 3:**
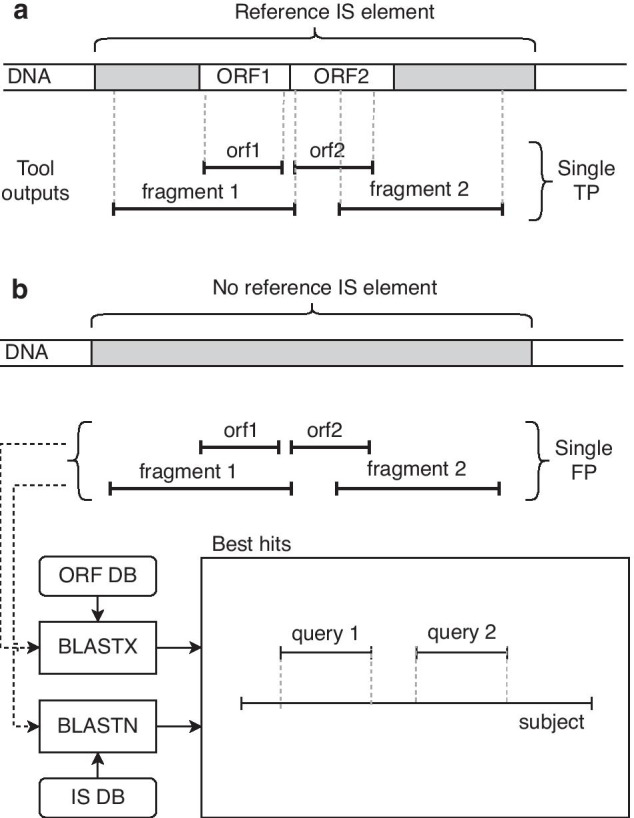
Definition of true positive and false positive with respect to the tools reporting IS element’s fragments or ORFs. **a** Definition of true positive: A hit is considered as a true positive (TP) if it overlaps with any item in the reference dataset. If multiple hits overlap with the same IS element in the reference dataset, then all of these hits count as one hit only. **b** Definition of false positive: Each reported hit of the tool is matched against the database of known IS elements (ISfinder), and if several adjacent hits map to the same IS element at DNA or ORF level, then these hits are counted as only one merged FP

Considering these facts and in an effort to evaluate the tools fairly, reported hits were classified as follows: A hit is considered as a true positive (TP) if it overlaps with any item in the reference dataset, and the length of the overlapping region is $\ge$ 100 bp^6^. If multiple hits overlap with the same IS element in the reference dataset, then all of these hits count as one hit only (as shown in Fig. 3a). A false negative (FN) is defined as a reference dataset element without sufficient overlap with at least one reported hit. A false positive (FP) represents a reported hit without sufficient overlap with at least one item from the reference dataset.

It turns out that some reference datasets may not be complete. For example, if a new IS element is discovered, it is not included in a previously published dataset. A hit matching this new IS element is considered as an FP, even if it was identified correctly by the tool (see “Evaluation on the benchmark ISbrowser and E. coli datasets” section). The number of FPs is then even higher for tools reporting ORFs or fragments of the same IS element. To minimize this side effect, each FP was compared with a database of known IS elements (ISfinder). If several adjacent FPs mapped to the same IS element at DNA or ORF level (as shown in Fig. 3b), they were counted as one merged FP (mFP).

### Evaluation on the benchmark ISbrowser and *E.coli* datasets

The first evaluation of the selected tools was performed on two benchmark datasets (1) a human-curated dataset from ISbrowser, and (2) the IS element annotation of *Escherichia coli* strain K-12 substr. MG 1655 genome [30]. The ISbrowser dataset comprises an annotation of 118 prokaryotic genomes (as of November 2017); 58 of them contain full-length IS elements, including 36 prokaryotic genomes and 22 plasmids. *E.coli* strain K-12 is one of the most well-understood model organisms [31] and is frequently used in microbial studies. The dataset of annotated IS elements for *E.coli* was obtained from the ISEScan publication (Supplementary Materials, Table 5) since EcoGene 3.0 [31], a source devoted to the structural and functional annotation of *E.coli* strain K-12, was unavailable at the time of manuscript preparation. This dataset consists of 49 IS elements of which 40 are full-length.

Results for the ISbrowser and *E.coli* datasets are shown in Table 2. Surprisingly, all tools showed a relatively high number of FPs and corresponding FDR (in the range from 8 to 24%). Therefore, we analyzed the FPs in more detail as follows: First, FPs representing fragments/ORFs of the same IS element were merged as described at the beginning of this section. Then, for each merged FP (mFP), the similarity with known IS elements in the ISfinder database was measured and by using the GenBank annotation it was classified into *IS-related*, *no annotation*, or *other annotation* category as described in “Output classification” section. Based on these results, a histogram was plotted depicting the number of mFPs as a function of similarity at both ORF and DNA levels. Finally, each bar in the histogram was divided according to the classification based on the GenBank annotation. These histograms represent an effective way to visualize the outputs of individual tools, including the identification of areas in which the tool makes errors. Please, see Additional file 4: “ISbrowser dataset” section.

In summary, many mFPs correspond to the hits that are highly likely to represent true IS elements that are not yet included in manually curated datasets. This behavior can be caused by the fact that the human-curated, whole-genome annotation might not be updated as often as databases of known IS elements. The exact numbers of true IS elements are unknown even in human-curated datasets and may evolve over time. Therefore, the common performance metrics, such as the confusion matrix, can not evaluate the tool quality fairly.

To address this issue, we decided to classify mFP hits further to distinguish between those representing IS elements with a high level of evidence and improbable/not IS elements. For these purposes, we used the GenBank annotation, which resulted from a conservative approach combining manually curated data and automatically predicted ones with a high level of confidence. Each mFP hit was classified according to the rules described in the “Output classification” section. Therefore, mFPs classified as *IS-related* can be highly likely considered as IS elements or their parts. Similarly, mFPs classified as *other annotation* can be regarded as improbable or not IS elements since they include parts that have been conservatively identified as other protein products.

The remaining hits classified as *no annotation* can be seen as unknown IS elements or those where the GenBank annotation pipeline has not achieved a sufficient level of confidence. To evaluate these outputs, additional information about sequence similarity with the database of known sequences (ISfinder) was used. Since the IS elements are divided into several independent families, it is difficult to find the exact boundary between IS and non-IS elements for mFPs. It is more appropriate to divide them into three categories:

- *Intra-family member*—a hit having similarity to the extent that is typical for members belonging to the same family.
- *Inter-family member*—a hit having similarity that is common among members of different families.
- *Improbable member*—a hit having similarity lower than usual among family members.

Although there may be several ways to categorize mFPs into these groups, we have chosen a more straightforward approach by defining two similarity thresholds (at the ORF and DNA level) that divide hits into these three categories. To determine the thresholds, a database of known IS elements (ISfinder) was used, the sequence similarities common within existing families and among them were measured, and these values were averaged. The resulting thresholds and their interpretations are given in Table 1. A detailed description of the procedure and the measured data is available in Additional file 5.

**Table 1:** Thresholds for classification based on the sequence similarity

Level/interpretation	Improbable member	Inter-family member	Intra-family member
IS element	SeqID < 50%	50% < SeqID $\le$ 70%	70% < SeqID
Tpase/ORF	SeqID < 25%	25% < SeqID $\le$ 45%	45% < SeqID

In summary, using the GenBank annotation and sequence similarity, the mFPs were classified into three categories according to the following rules:

- *IS element with a high level of evidence (eIS)*—a hit classified as *IS-related* based on the GenBank annotation, or a hit classified as *no annotation* based on the GenBank and *Intra-family member* based on the sequence similarity.
- *Distant or putative novel IS element (pNov)*—a hit classified as *no annotation* based on the GenBank and *Inter-family member* based on the sequence similarity.
- *Improbable or not an IS element (nIS)*—a hit classified as *other annotation* based on the GenBank annotation, or a hit classified as *no annotation* based on the GenBank and *Improbable member* based on the sequence similarity.

Distribution of mFP entries into these three categories is presented in Table 3, columns labeled as *Detailed classification of mFPs*. It can be seen that a large part of the hits initially classified as mFPs falls into the category *IS element with a high level of evidence*. Together with previously identified TPs, they represent the total number of IS elements with a high level of evidence (teIS). Consequently, only the hits in the nIS category are considered to be incorrectly identified by the tool (i.e. false positives). Based on these new metrics, the putative novel discovery rate (pNovDR), and nIS discovery rate (nISDR) were calculated representing the proportion of putative novel and improbable/not IS elements in reported outputs, respectively. Finally, the pNov/nIS ratio was calculated to express how many putative novel elements are found per single incorrectly identified hit.

We presume that these modified metrics reflect the tools’ performance better since they address the issue of incomplete reference datasets. Concurrently, they are based on sequence similarity information with known IS elements (ISfinder) and state-of-the-art annotations with high confidence (GenBank). We are aware of possible discussions and alternatives towards defined classification rules and similarity thresholds. However, if they are applied to all tools equally, they can bring a more reliable image of their performance.

The results in Table 3 related to the ISbrowser dataset show that:

- The tools that detect both full elements and fragments (ISsaga and ISEScan–fragments) can find the highest number of teISs. On the other hand, the reported hits include the highest number of nISs. The overall nISDR is around 9%, and the ratio between pNovs and nISs is low (0.15 and 0.22).
- OASIS found the lowest number of teISs and nISs (nISDR is 1.15%), making it the most conservative tool of all. OASIS found only the hits with a high level of confidence. The output primarily includes records of known IS elements, whereas putative novel elements are rare (0.69%).
- ISEScan is the second most conservative tool in terms of the number of teISs and nISs. Surprisingly, it found even less pNovs compared to the OASIS tool.
- With respect to the number of teISs and nISs, *digIS* falls in the middle between conservative (OASIS and ISEScan) and fragment-reporting tools (ISEScan–fragments and ISsaga) representing a tool with good sensitivity (0.82) and low nISDR (3.58%). Moreover, the number of pNovs is even higher than for ISEScan–fragments. Although ISsaga found one-third more pNovs than *digIS*, it was at the cost of three times more nISs.

The tools show a similar performance on the *E.coli* dataset. However, some characteristics are violated; for instance, none of the tools found any putative novel element, and nISDR is more than double for most tools. These discrepancies are primarily caused by a too small *E.coli* dataset (a single genome with less than 50 IS elements), where some of the metrics are calculated from fewer than ten items. Similar distortion can also be seen in the ISbrowser dataset, where the numbers of pNovs and nISs are too small for the OASIS tool. It results in a disproportionately high pNov/nIS ratio.

### Evaluation on the NCBI Archaea and Bacteria datasets

In the next step, tools were evaluated on much larger datasets to verify the characteristics observed in Table 3 and to specify those affected by the small number of samples. We prepared two additional datasets containing complete archaeal and bacterial genomes from the NCBI GenBank database [32]. In the case of Archaea, all 341 genomes available in the database were used (accessed on 15th June 2019). In the case of Bacteria, 2500 from 14418 available genomes were randomly selected (see Additional file 6 for detailed information about these datasets). Since OASIS could not process 25 bacterial genomes, these were excluded. Altogether, 2475 bacterial genomes were evaluated.

Unlike the ISbrowser and *E.coli* datasets, the manually curated positions of IS elements are not available. Therefore, all hits reported by the tools were considered as FPs and the detailed classification process of FPs described in “Evaluation on the benchmark ISbrowser and E. coli datasets” section was applied. To verify the accuracy of this evaluation method, it was applied to the ISbrowser dataset first. Table 4 shows the number of hits found by the tool (N), the number of merged FPs (mFPs), the output of the classification process (number of eISs, pNovs, and nISs), and an assessment in terms of pNovDR, nISDR, and pNov/nIS ratio. As the number of TPs is not available, the teIS is reduced to eIS.

By comparing the evaluation results for the ISbrowser dataset with and without human-curated annotation (Tables 3, 4), certain differences can be seen. Detailed analysis revealed that these changes arose primarily because the ISbrowser reference dataset contains not only full-length elements, but also annotated fragments of various lengths (a total of 127 fragments). If a tool finds some of these fragments, they are distributed among the categories eIS, pNov, and nIS based on the GenBank annotations and similarities with the ISfinder database. This behavior causes the number of pNovs and nISs to increase at the expense of the total number of eIS. As a side effect, the pNovDR, nISDR, and pNov/nIS ratio are slightly higher. The small changes can also be observed in the histograms (see Additional file 4: “ISbrowser dataset without reference” section), but their overall character remains the same. Considering these subtle differences, it is possible to conclude that the above-described classification allows us an assessment of the tool performance, even when the manually curated annotation is not available.

The results on large NCBI GenBank Archaea and Bacteria datasets in Table 4 confirmed the tools’ characteristics seen on the ISbrowser dataset. Only the following differences were observed:

- The proportion of nISs in the outputs is higher compared to the ISbrowser dataset. For ISEScan and *digIS*, the nISDR is approximately twice as large on the Archaea dataset. ISsaga achieved the highest nISDR (around 20%) for both Archaea and Bacteria datasets. A detailed analysis of the hits revealed that this is primarily due to the higher number of items classified as *other annotation*. A list of the most common GenBank record products that overlapped with these hits is given in Additional file 7.
- Larger NCBI datasets enabled to assess the ratio between pNov and nIS for OASIS more accurately, as it was affected by a small number of items in the *E.coli* and ISbrowser datasets before. This ratio decreased significantly to 0.27 and 0.21. Also, the number of pNovs found by OASIS is no longer higher than those found by the ISEScan tool.
- The histograms depicting the similarity of the outputs with the ISfinder database and their classification according to the GenBank annotation show the same characteristics as for the ISbrowser dataset, except for minor deviations (see Additional file 4: “NCBI Archaea and Bacteria datasets without reference” section).

In summary, tools that also detect fragments (ISsaga and ISEScan–fragments) can identify the most eISs, but at the cost of a large number of nISs. On the other side of the spectrum are conservative tools (OASIS and ISEScan), which show the lowest numbers of nISs, but also eISs. The performance of the proposed *digIS* tool in terms of eISs is closer to fragment-reporting tools, and at the same time, it achieves the number of nISs closer to conservative tools. Moreover, *digIS* is dominant in finding distant/putative novel IS elements with respect to the numbers of nISs (pNov/nIS ratio). This feature is significant, especially on large datasets (NCBI GenBank Archaea/Bacteria), where the *digIS* tool shows the best performance. Please note that *digIS* found even more putative novel elements than the ISEScan–fragments in these datasets.

## Discussion

In this work, we focused on the detection of putative novel IS elements and aimed to find the sequence and structural features common to more IS families. The Tpases are generally considered as the most conserved parts of IS elements. Their structural variability is used as a major feature for their classification into the families [1]. On the other hand, the Tpase catalytic domain and its secondary structure are often preserved among the families [25]. Unfortunately, the accuracy of state-of-the-art tools for secondary structure prediction is not sufficient when applied to a single sequence and MSA is usually required for a more accurate prediction [33].

For this reason, we decided to make a compromise between detecting the general structure and sequence features. We built the library of manually curated pHMMs of a catalytic domain only (not whole transposase). The results of comparing *digIS* with other tools confirmed that the search based on the catalytic domain is sufficiently specific for the area of IS elements. The number of IS elements with a high level of evidence is comparable to fragment-reporting tools, while many improbable/not IS elements are filtered out. To better understand the effectiveness of the catalytic-domain-search technique compared to using the pHMM of the whole Tpase sequence, we performed a detailed analysis of individual tools’ outputs. We focused on hits classified as “other annotation” according to the GenBank annotation, i.e., the records erroneously identified by the tool as IS elements or their parts. We analyzed overlapping GenBank records for these hits and created a histogram showing the number of occurrences for each type of protein or product (see Additional file 7).

From the generated histograms, it can be observed that *digIS* generally reports a small number of records classified as “other annotation”, which is comparable to conservative tools such as OASIS or ISEScan (see Additional file 7; Tables 1, 2, 3). On the other hand, tools that also report fragments (ISsaga and ISEScan–fragments) show a large number of these hits. If we focus on the annotations of these records, it can be seen that they usually represent products functionally related to transposases or parts thereof, such as *DNA-binding protein*, *ATP-binding protein*, *transcriptional regulator*, or *helix-turn-helix domain-containing protein*. In addition, both fragment-reporting tools (ISsaga and ISEScan–fragments) cover a large number of products that were not observed by other tools, including *digIS*, such as *chromosomal replication initiator protein DnaA*, *DNA replication protein DnaC*, or *primosomal protein DnaI*. Detailed analysis revealed that portions of these proteins have significant sequence similarity to the coding segments of IS elements of the IS21 family (see Additional file 7). These examples show that searching for any fragments of IS elements can lead to a large number of false hits, which the application user must manually check. On the other hand, focusing the search on the catalytic domain can effectively filter these hits and, unlike conservative methods reporting full-length elements only, it provides a space for searching for putative novel IS elements.

**Table 2:** Performance of OASIS, ISEScan, ISEScan-fragments, ISsaga, and digIS on manually curated datasets

Tool	TP	FN	FP	Se	FDR (%)
Dataset ISbrowser (N = 1192)					
OASIS	791	401	77	0.66	8.87
ISEScan	925	267	94	0.78	9.22
ISEScan-fragments	1077	115	248	0.90	18.71
ISsaga	1135	57	363	0.95	24.23
digIS	979	213	194	0.82	16.54
Dataset *E. coli* (N = 49)					
OASIS	26	23	4	0.53	13.33
ISEScan	43	6	8	0.88	15.69
ISEScan-fragments	45	4	18	0.92	28.57
ISsaga	48	1	29	0.98	37.66
digIS	43	6	11	0.88	20.37

TP, FN, and FP represent the number of True Positives, False Negatives, and False Positives, respectively; Se is sensitivity; FDR is False Discovery Rate.

**Table 3:** Detailed analysis of false positives of digIS, ISEScan, OASIS, and ISsaga on manually curated

Tool	Common metrics		Detailed classification of mFPs				Modified metrics			
	TP	FP	mFP	eIS	pNov	nIS	teIS	pNovDR (%)	nISDR (%)	pNov/nIS
Dataset ISbrowser (N = 1192)										
OASIS	791	77	75	59	6	10	850	0.69	1.15	0.60
ISEScan	925	94	94	69	3	22	993	0.29	2.16	0.14
ISEScan-fragments	1077	248	239	103	18	118	1179	1.37	8.97	0.15
ISsaga	1135	363	323	148	31	144	1282	2.13	9.88	0.22
digIS	979	194	194	130	22	42	1108	1.88	3.58	0.52
Dataset *E. coli* (N = 49)										
OASIS	26	4	4	4	0	0	30	0.00	0.00	0.00
ISEScan	43	8	7	3	0	4	46	0.00	8.00	0.00
ISEScan-fragments	45	18	17	6	0	11	51	0.00	17.74	0.00
ISsaga	48	29	28	10	0	18	58	0.00	23.68	0.00
digIS	43	11	11	6	0	5	49	0.00	9.26	0.00

N represents the number of outputs found by the tool; mFP represents the number of False Positives after merging fragments or ORFs referencing the same IS element; eIS, pNov, and nIS represent the number of mFPs classified into categories IS element with a high level of evidence, Distant or putative novel IS element, and Improbable or not an IS element, respectively; pNovDR is putative Novel Discovery Rate; nISDR is Improbable or not an IS element Discovery Rate, and pNov/nIS shows the ratio between the number of putative novel IS elements and improbable or not an IS elements.

When comparing the tools without a manually curated reference dataset or an incomplete one, the histogram—showing the number of outputs depending on the similarity to the database of known elements (ISfinder) and GenBank annotation—is a useful indicator of the tool’s quality. It offers an independent view of the characteristics of the outputs and clearly shows, for example, the degree of tool conservation or tendency to detect other genes, that is typical for fragment-reporting tools (ISsaga and ISEScan–fragments). It also allows the identification of various anomalies in the GenBank annotation itself (see Additional file 4).

Despite the histogram’s benefits, it does not allow us to easily quantify and compare the performance of the tools. The comparison is possible only if the outputs are classified into distinct categories such as TPs, TNs, FPs, FNs using manually curated benchmark datasets. In this paper, we were the first to point out the drawbacks of this approach when applied to existing tools for IS elements detection. We addressed the issue of different outputs of individual tools (full-length elements vs. fragments/ORFs). Based on a detailed analysis (see Additional file 4), we have shown that the benchmark datasets themselves are not complete, and therefore their use may skew the evaluation results.

To overcome these issues, we have chosen an alternative classification of the tools’ outputs that relies on GenBank annotation and sequence similarity with the database of known elements (ISfinder). This approach allowed us to identify a group of IS elements with a high level of evidence (eIS) and a group of Improbable or not IS elements (nIS) in the category of presumed false positives. Also, since the boundary between these two groups is not strictly defined, there is a space for the putative novel IS elements group (pNov), which is the main interest of this article. We are aware that the definition of these categories is unambiguous and should be replaced by a high-quality and consistently maintained benchmark dataset in the future. On the other hand, the boundary between the groups of pNovs and nISs will probably be the subject of debate for a long time, as its precise definition would require a knowledge of all non-IS elements.

We experimented, for example, with a different definition of pNov and its effect on tools performance. Currently, pNov is defined as a sequence without a sufficiently specific GenBank annotation, having the sequence similarity that is common among members of different IS families. Without further restrictions, this category may include, for example, the found accessory genes or some of the transposase’s variable domains. To make sure that the found hit is highly likely functional from a transposition point of view, it would be appropriate to require the presence of Tpase and its catalytic domain. Therefore, an analysis of the pNov hits was performed and those that overlap with the catalytic domain of any known IS element were identified (see Additional file 8). This analysis showed that many hits fall outside the catalytic domain, especially for fragment-reporting tools (ISsaga and ISEScan–fragments). If the tools were evaluated according to this stricter definition, then the proposed *digIS* would achieve the best results in the detection of pNovs on an absolute scale.

We analyzed the coverage of pNovs by individual tools to identify which of them are reported by several tools simultaneously or, conversely, exclusively by a specific tool. We also measured pNovs regarding their proximity to existing families of IS elements to reveal a possible preference of the tool to search for pNovs in a certain part of the sequence space (see Additional file 8). It turned out that various tools have a preference to search pNov elements close to various IS families. For example, *digIS* found the most pNovs close to the ISH3 family while ISsaga found the most pNovs close to the IS5 family. In summary, it can be concluded that no tool would include all pNov outputs of other tools.

Finally, we performed an analysis of the found pNovs to verify that they met the common characteristics of IS elements, such as multiple occurrences in the genome, or the presence of IR and DR regions. Using clustering, we found groups of similar hits, then performed their multiple sequence alignment, and identified IR and DR regions. Based on a manual inspection of selected clusters, we have identified four novel IS elements, of which the first two can be found by competing tools and the other two represent new ones found exclusively by the *digIS* tool (see Additional file 9).

**Table 4:** Performance of digIS against ISEScan, OASIS, and ISsaga on NCBI GenBank datasets

Tool	N	Detailed classification of mFPs				Modified metrics		
		mFP	eIS	pNov	nIS	pNovDR (%)	nISDR (%)	pNov/nIS
Dataset ISbrowser (N = 1192)								
OASIS	895	852	828	10	14	1.17	1.64	0.71
ISEScan	1006	993	954	9	30	0.91	3.02	0.30
ISEScan-fragments	1326	1283	1089	41	153	3.20	11.93	0.27
ISsaga	1786	1459	1188	75	196	5.14	13.43	0.38
digIS	1170	1157	1051	50	56	4.32	4.84	0.89
Dataset NCBI Archaea (341 genomes)								
OASIS	5885	5789	5382	100	307	1.73	5.30	0.33
ISEScan	8404	8266	7532	207	527	2.50	6.38	0.39
ISEScan-fragments	12,016	11,550	9622	472	1456	4.09	12.61	0.32
ISsaga	17,698	14,788	10,946	822	3020	5.56	20.42	0.27
digIS	10,607	10,548	8640	728	1180	6.90	11.19	0.62
Dataset NCBI Bacteria (random selection of 2475 genomes)								
OASIS	88,552	87,428	83,992	1176	2260	1.35	2.58	0.52
ISEScan	111,974	110,357	102,266	3274	4817	2.97	4.36	0.58
ISEScan-fragments	151,540	145,248	119,392	6096	19760	4.20	13.60	0.31
ISsaga	217,345	181,880	136,903	8479	36,498	4.66	20.07	0.23
digIS	134,851	132,877	118,805	6722	7350	5.06	5.53	0.91

N represents the number of outputs found by the tool; mFP represents the number of False Positives after merging fragments or ORFs referencing the same IS element; eIS, pNov, and nIS represent the number of mFPs classified into categories IS element with a high level of evidence, Distant or putative novel IS element, and Improbable or not an IS element, respectively; pNovDR is putative Novel Discovery Rate; nISDR is Improbable or not an IS element Discovery Rate, and pNov/nIS shows the ratio between the number of putative novel IS elements and improbable or not an IS elements.

## Conclusions

In this paper we present a novel approach for IS elements detection, that is implemented in the form of *digIS* tool. It combines searching for the catalytic domains of transposases and additional filtering mechanisms that allows to detect not only known IS elements, but also distant putative novel IS elements. Simultaneously, it eliminates a large number of false hits that are typical for fragment–reporting tools.

Comparison with other state-of-the-art tools, such as ISsaga, OASIS, and ISEScan, on different datasets (*E.coli*, ISbrowser, NCBI GenBank Archaea/Bacteria) confirmed that *digIS* can find the majority of known ISs and shows the best ratio between putative novel elements and improbable/not IS elements. This makes it the right choice for scientists who are interested in finding new IS elements.

Finally, we would also highlight the technical aspects of the developed software. *digIS* is one of the few tools that still works and is ready for future use in the form of a Docker image. Simultaneously, it does not limit the user in the number of sequences to be analyzed or other search parameters, as is the case of web-based tools. *digIS* is ready to run in a grid-computing and cloud environment, which is very important for scalability. The transparency and credibility of the tool are further supported by the open-source code on GitHub (Table 4).

## Supplementary information

**Additional file 1.** Multiple sequence alignment of IS5/IS5 subfamily.

**Additional file 2.** Multiple sequence alignment of IS5/None subfamily.

**Additional file 3.** Multiple sequence alignment of ISL3 family.

**Additional file 4.** Analysis of merged FPs.

**Additional file 5.** Calculation of the similarity at IS and ORF level.

**Additional file 6.** Detailed information about NCBI GenBank archaeal and bacterial genomes used in the evaluation.

**Additional file 7.** Analysis of hits classified as *other annotation.*

**Additional file 8.** Analysis of putative novel elements.

**Additional file 9.** Putative novel IS elements detected by *digIS*.

## Data Availability

The reference genomes used during the current study are publicly available and were downloaded from the NCBI GenBank database (https://www.ncbi.nlm.nih.gov/genbank). The detailed instructions for obtaining sequences included in the NCBI Archaea and Bacteria datasets are described in Additional file 6. The manually annotated IS elements for *E.coli* were obtained from the ISEScan publication (Supplementary Materials, Table 5) since EcoGene 3.0 [31], a source devoted to the structural and functional annotation of *E.coli* strain K-12, was unavailable at the time of manuscript preparation.

## Abbreviations

bp: Base pair
CDS: Coding sequence
DR: Direct repeat
DUF: Domain of unknown function
eIS: IS element with a high level of evidence
FN: False negative
FP: False positive
IR: Inverted repeat
IS: Insertion sequence
kbp: Kilobase pair
MSA: Multiple sequence alignment
mFP: Merged false positive
nIS: Improbable or not an IS element
nISDR: Improbable/not an IS element discovery rate
ORF: Open reading frame
pNov: Putative novel
pNovDR: Putative novel discovery rate
pHMM: Profile hidden Markov model
TP: True positive
teIS: Total number of IS element with a high level of evidence

## Footnotes

1 Various IS families carry Tpase consisting of multiple ORFs. These ORFs are present in the ISfinder database in both individual and fusion forms. As duplicated sequences may lead to a bias in pHMMs, only the longest ORF sequence was used.

2 Merge distance 700 bp was identified based on the longest gaps within the models (see Additional file 3 for more details).

3 ORF context size 1600 bp was identified based on the length of the longest transposase ORF in the ISfinder database divided by 2, multiplied by 3 (conversion from amino acids to nucleotides) and rounded up to the nearest hundredth

4 DNA context size 14000 bp was identified based on the length of the longest IS element in the ISfinder database divided by 2 and rounded up to the nearest hundredth

5 GenBank annotation is a result of a complex process [26] that utilizes sources of manually curated data and automatically predicted ones with a high level of confidence.

6 Usually, an overlap based on a percentage of the reference IS element length is used in other studies, but when allowing for fragments, this criterion is not applicable. The requirement for at least 100 bp overlap seems to work well, even when two neighboring IS elements overlap.
