# Supplementary figures and images for: digIS: towards detecting distant and putative novel insertion sequence elements in prokaryotic genomes
Source: BMC Bioinformatics. 2021 May 20;22:258. doi: 10.1186/s12859-021-04177-6 (PMC8147514; doi:10.1186/s12859-021-04177-6)

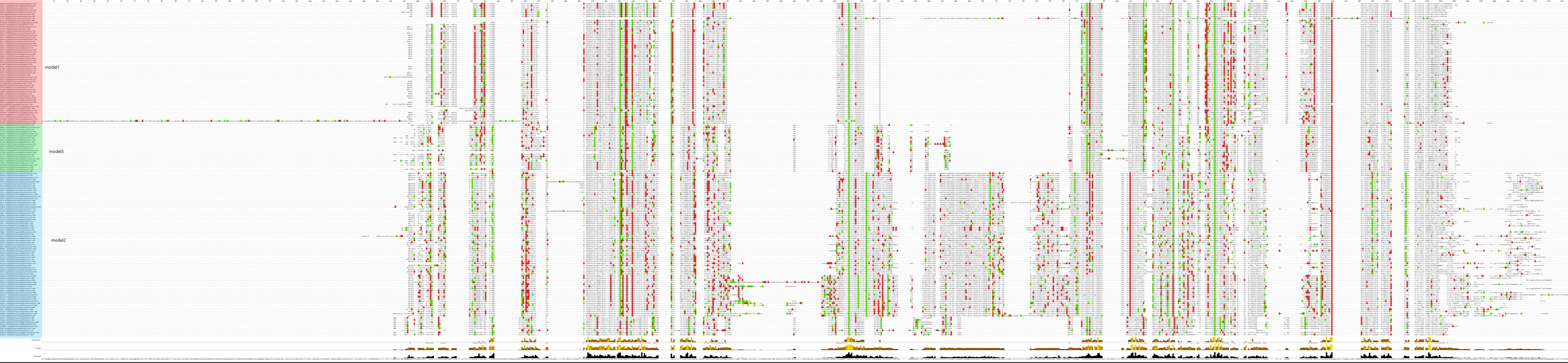

Supplement: Supplementary file 1 — Additional file 1. Multiple sequence alignment of IS5/IS5 subfamily. [file 12859_2021_4177_MOESM1_ESM.png]

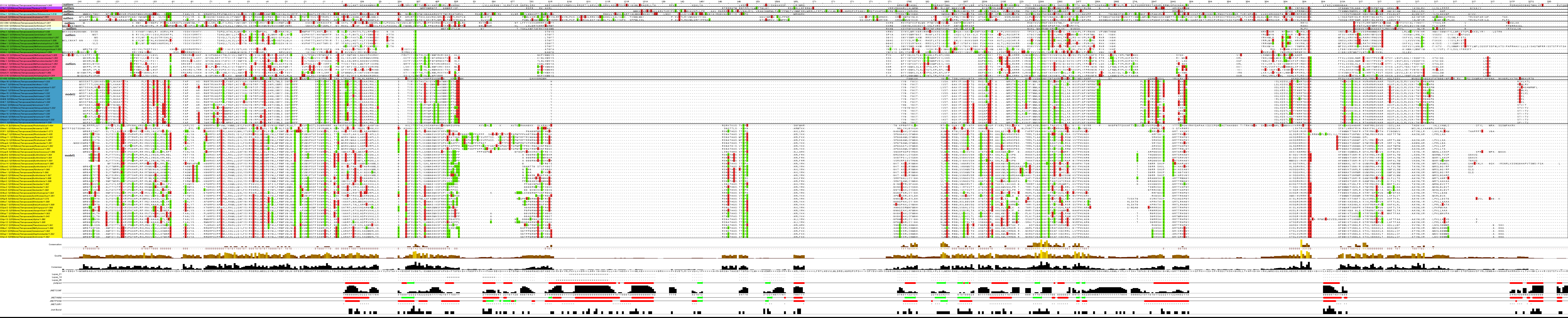

Supplement: Supplementary file 2 — Additional file 2. Multiple sequence alignment of IS5/None subfamily. [file 12859_2021_4177_MOESM2_ESM.png]

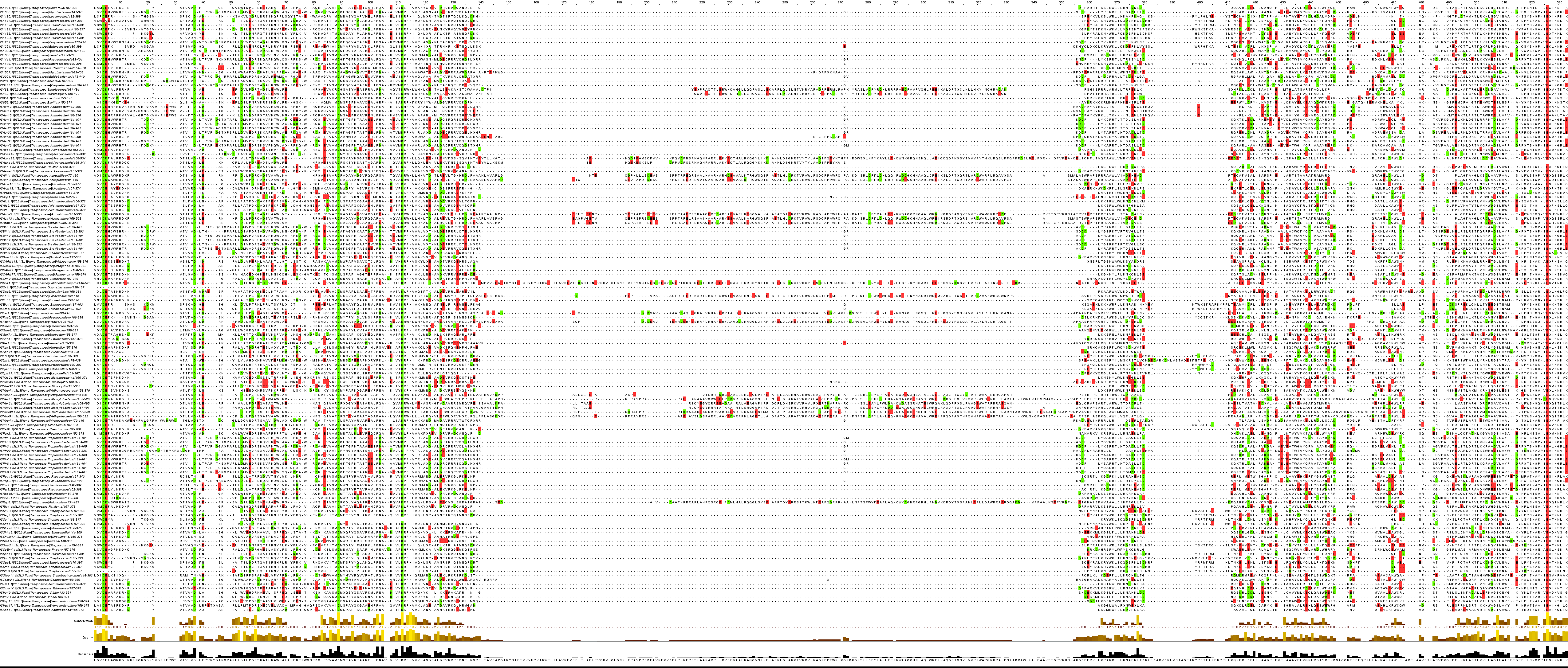

Supplement: Supplementary file 3 — Additional file 3. Multiple sequence alignment of ISL3 family. [file 12859_2021_4177_MOESM3_ESM.png]
