# Supplementary material for: digIS: towards detecting distant and putative novel insertion sequence elements in prokaryotic genomes
Source: BMC Bioinformatics. 2021 May 20;22:258. doi: 10.1186/s12859-021-04177-6 (PMC8147514; doi:10.1186/s12859-021-04177-6)
Supplement: Supplementary file 4 — Additional file 4. Analysis of merged FPs. [file 12859_2021_4177_MOESM4_ESM.docx]

# Analysis of merged FPs

Goals:

- Find the reason why all tools reported many mFP hits on a manually curated ISbrowser dataset.
- Depict the relationship between sequence similarity with a database of known IS elements (ISfinder) and the GenBank annotation of found hits.

Procedure:

1. For each mFP, the similarity with known IS elements in the ISfinder database was measured and a classification was performed based on the GenBank annotation and the rules described in section *Output classification*.
2. Based on the results, a histogram was built showing the numbers of mFPs as a function of similarity at the ORF and DNA level.
3. Each histogram bar was further divided according to the mFP classification based on GenBank annotation into *IS-related*, *no annotation,* and *other annotation* groups.

## ISbrowser dataset

The histograms depicted in Figure 1 and 2 show the following characteristics:

- All tools report many hits classified as *IS-related* that have a high similarity with the ISfinder database^^[[1]](#footnote-0)^^. These hits are highly likely to be true IS elements that have not yet been included into the manually-curated dataset.

As expected, tools with a conservative approach (OASIS and ISEScan) report less of these hits compared to fragments-reporting tools (ISsaga and ISEScan-fragments). Although *digIS* is not focused on fragment detection, it surprisingly reports more hits than ISEScan-fragments.

- On the other side of the spectrum, tools often report many items with low similarity^^[[2]](#footnote-1)^^. This behavior is typical for fragment-reporting tools (ISsaga and ISEScan-fragments). Many fragments are classified as *other annotation*, which means that the tool most likely found a part of another protein or product.

Surprisingly, some hits with low similarity are also classified as *IS-related*. A more detailed analysis showed that these are often genomic regions annotated as transposases, which are not included in the ISfinder database and may not be part of true IS elements. These entries usually have zero similarity at the DNA level but show low/medium similarity at the ORF level. This behavior is also seen when comparing the similarity graphs at the ORF and DNA level. While the number of low-similarity hits is higher at the DNA level, at the ORF level, these hits extend more along the x-axis.

- Finally, the graphs show the areas in which the tools make errors and report hits classified as *other annotation*. Please note that, in addition to the already mentioned low-similarity hits, the number of hits classified as *other annotation* gradually increases from 0.4 below for ISEScan-fragments. The same behavior is not seen for ISsaga, where an increased number of these hits can be observed around 0.4 similarity only. A more detailed analysis shows that these hits usually overlap with GenBank records annotated as 'transcriptional regulator.' It indicates the specific properties of the models used by individual tools and their tendencies to find non-IS elements.

Tools using a conservative approach report very few hits classified as *other annotation* (ISEScan) or even none (OASIS). *digIS* also reports a very low number of outputs of this type, despite the fact that it searches a selected part of an IS element - the catalytic domain of transposase. This finding confirms that the catalytic domain is sufficiently specific for IS element detection and does not interfere with the entries of other proteins or products.

## ISbrowser dataset without reference

The histograms have similar characteristics to ISbrowser datasets with manually-curated annotations (see Figures 3A,B and 4A,B), except for the following differences:

- As expected, a high number of hits, classified as *IS-related* with high similarity to the ISfinder database, can be observed at both the DNA and ORF level. These hits are usually full-length elements that were classified as TPs in the reference dataset.
- A higher number of hits classified as *IS-related* can also be observed at all similarity levels. A more detailed analysis revealed that it is caused by the fragments of IS elements that are also included in a manually-curated ISbrowser dataset (up to 127 fragments). The fragments that the tool found were then distributed in the histogram along the x-axis according to their similarity.
- Finally, a slight increase in hits classified as *other annotation* can also be observed. This is because some fragments are also annotated as other proteins or products based on the GenBank annotation.

## NCBI Archaea and Bacteria datasets without reference

The histograms have characteristics similar to ISbrowser datasets without reference (see Figures 5A,B, 6A,B, 7A,B and 8A,B), except for the following differences:

- A higher number of outputs classified as *other annotation* can be observed at all levels of similarity, especially for fragment-reporting tools (ISSaga and ISEScan-fragments). ISsaga reports this type of hits at the lowest levels of similarity (~11k for Bacteria) compared to ISEScan-fragments (~5k for Bacteria), which spreads hits along the x-axis.
- ISsaga also reports the most hits classified as *IS-related* at the lowest level of similarity (~10k for Bacteria) compared to other tools (~1.8k for Bacteria). A detailed analysis revealed that these hits often overlapped with GenBank records annotated as recombinase (~5800) and transposase (~3600). This behavior does not occur in the Archaea dataset.
- All tools (except OASIS) report many hits with high similarity but classified as *no annotation* in the Bacteria dataset (~6k). These hits are highly likely to be IS elements from the IS1 and IS3 family, which are not yet part of the GenBank annotation pipeline or the older tools (OASIS).


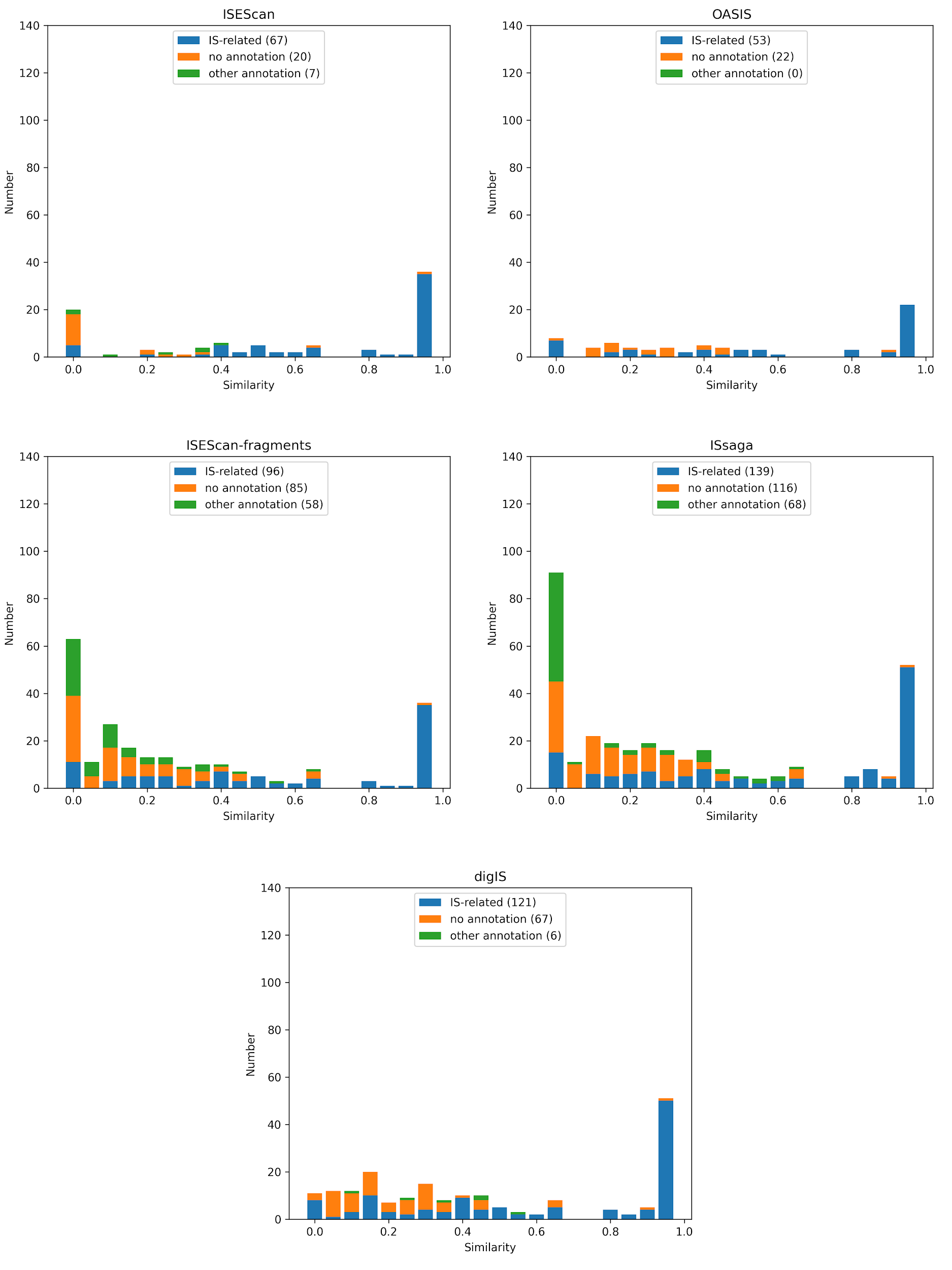


Figure 1: ISbrowser dataset -- Histogram depicting the number of mFPs as a function of their similarity to the ISfinder database (at ORF level) and their classification according to the GenBank annotation.


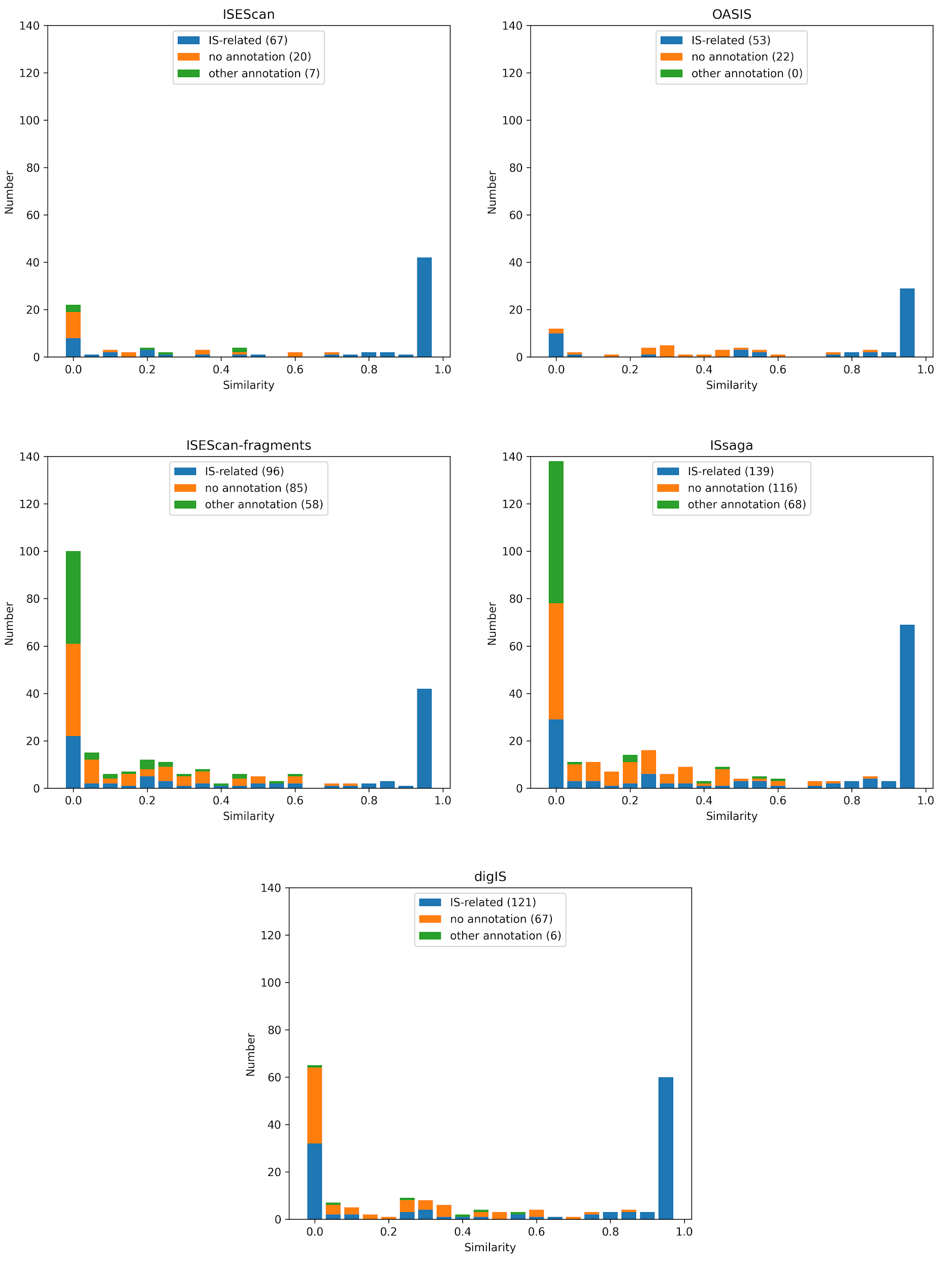


Figure 2: ISbrowser dataset -- Histogram depicting the number of mFPs as a function of their similarity to the ISfinder database (at DNA level) and their classification according to the GenBank annotation.


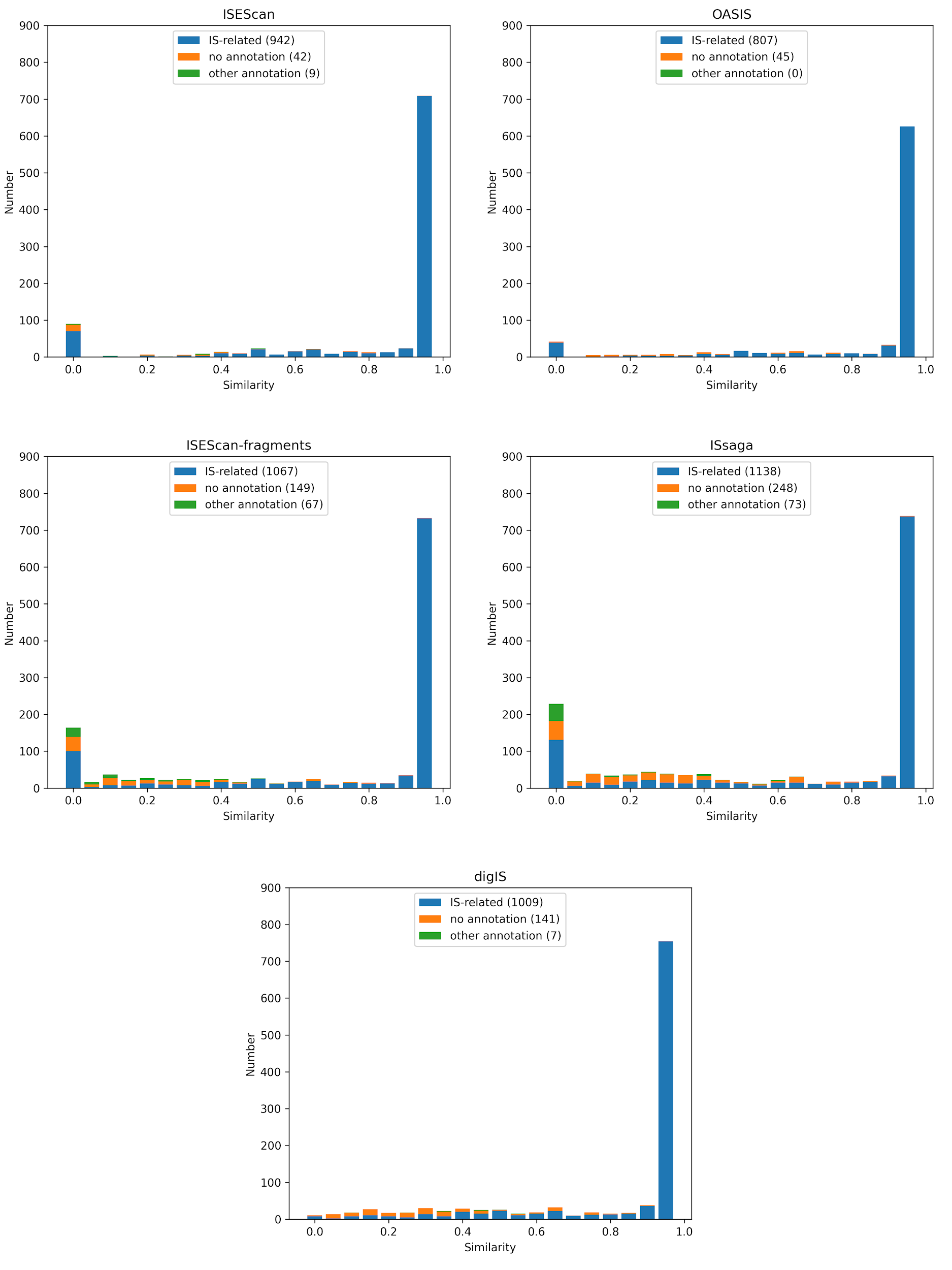


Figure 3A: ISbrowser dataset without reference -- Histogram depicting the number of mFPs as a function of their similarity to the ISfinder database (at ORF level) and their classification according to the GenBank annotation.


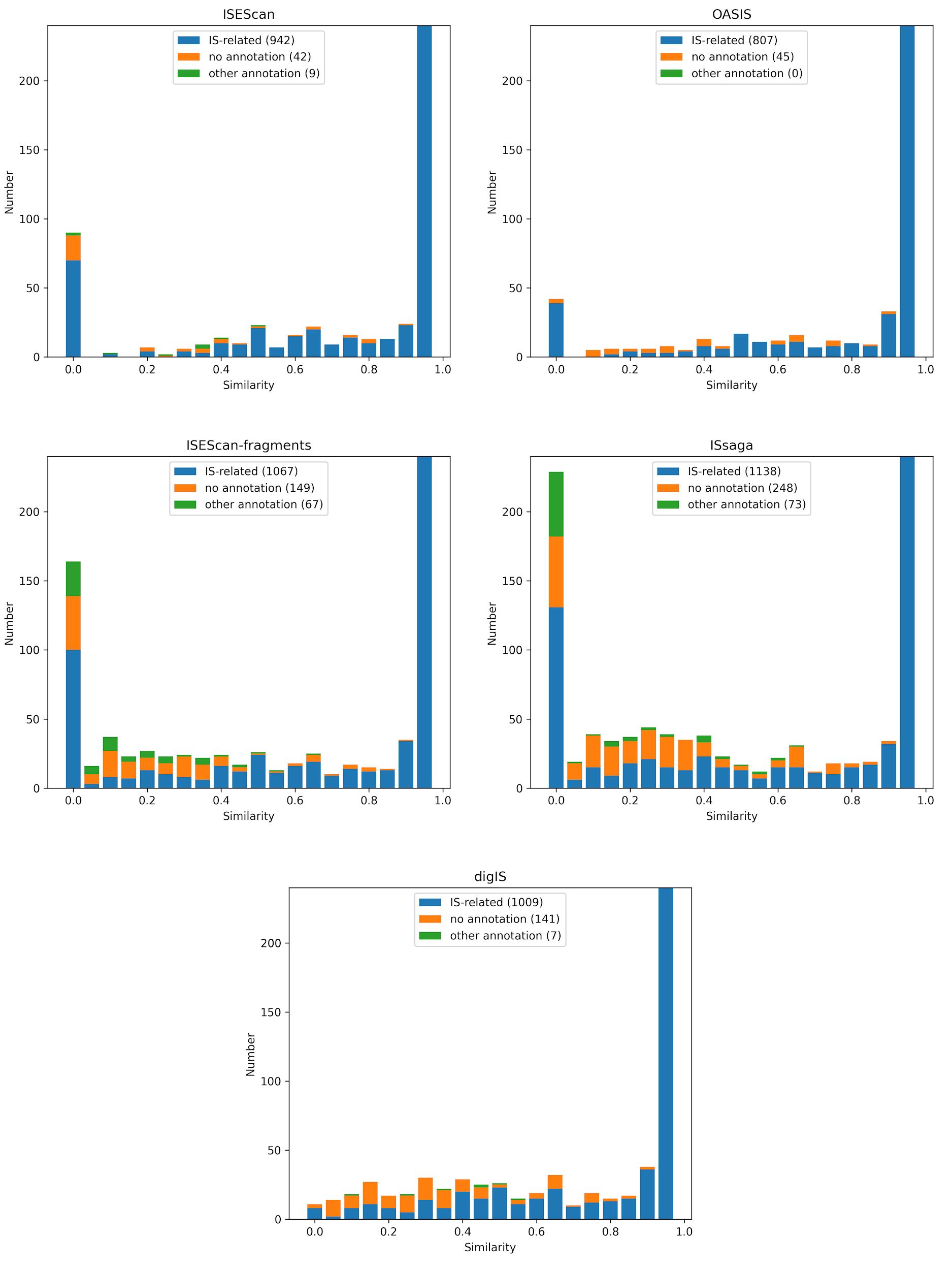


Figure 3B: ISbrowser dataset without reference -- Enlarged view of the histogram depicting the number of mFPs as a function of their similarity to the ISfinder database (at ORF level) and their classification according to the GenBank annotation.


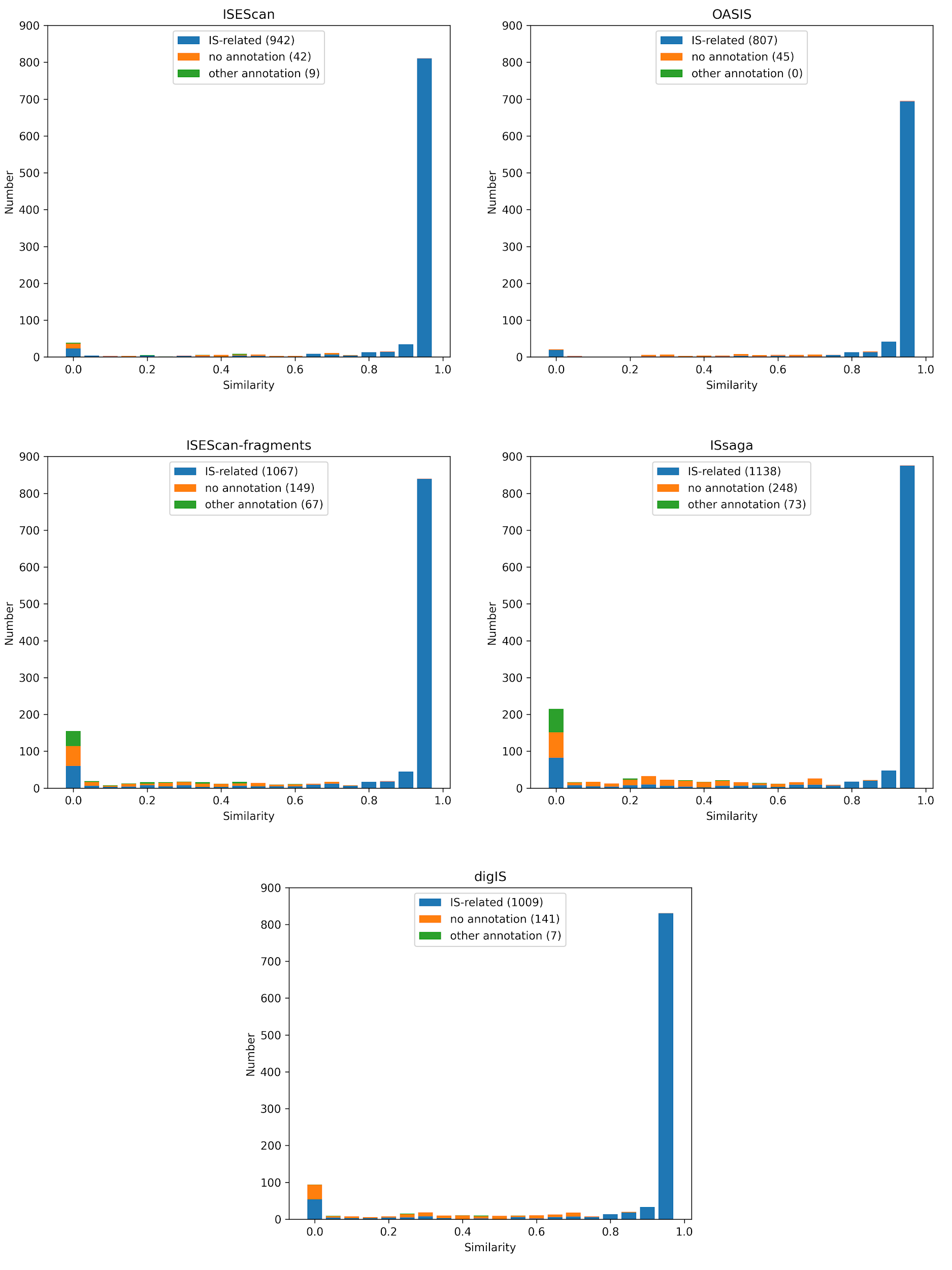


Figure 4A: ISbrowser dataset without reference -- Histogram depicting the number of mFPs as a function of their similarity to the ISfinder database (at DNA level) and their classification according to the GenBank annotation.


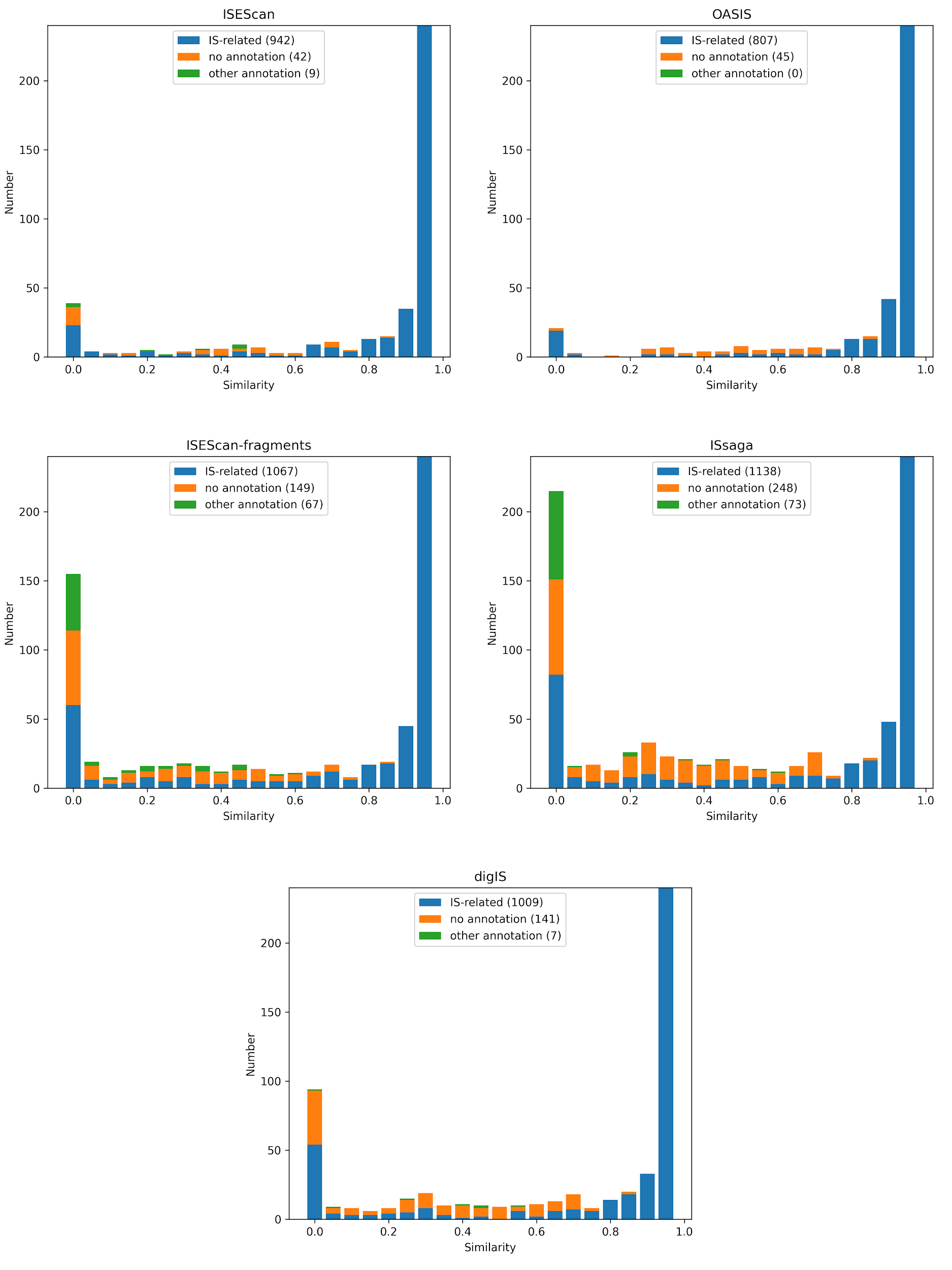


Figure 4B: ISbrowser dataset without reference -- Enlarged view of the histogram depicting the number of mFPs as a function of their similarity to the ISfinder database (at DNA level) and their classification according to the GenBank annotation.


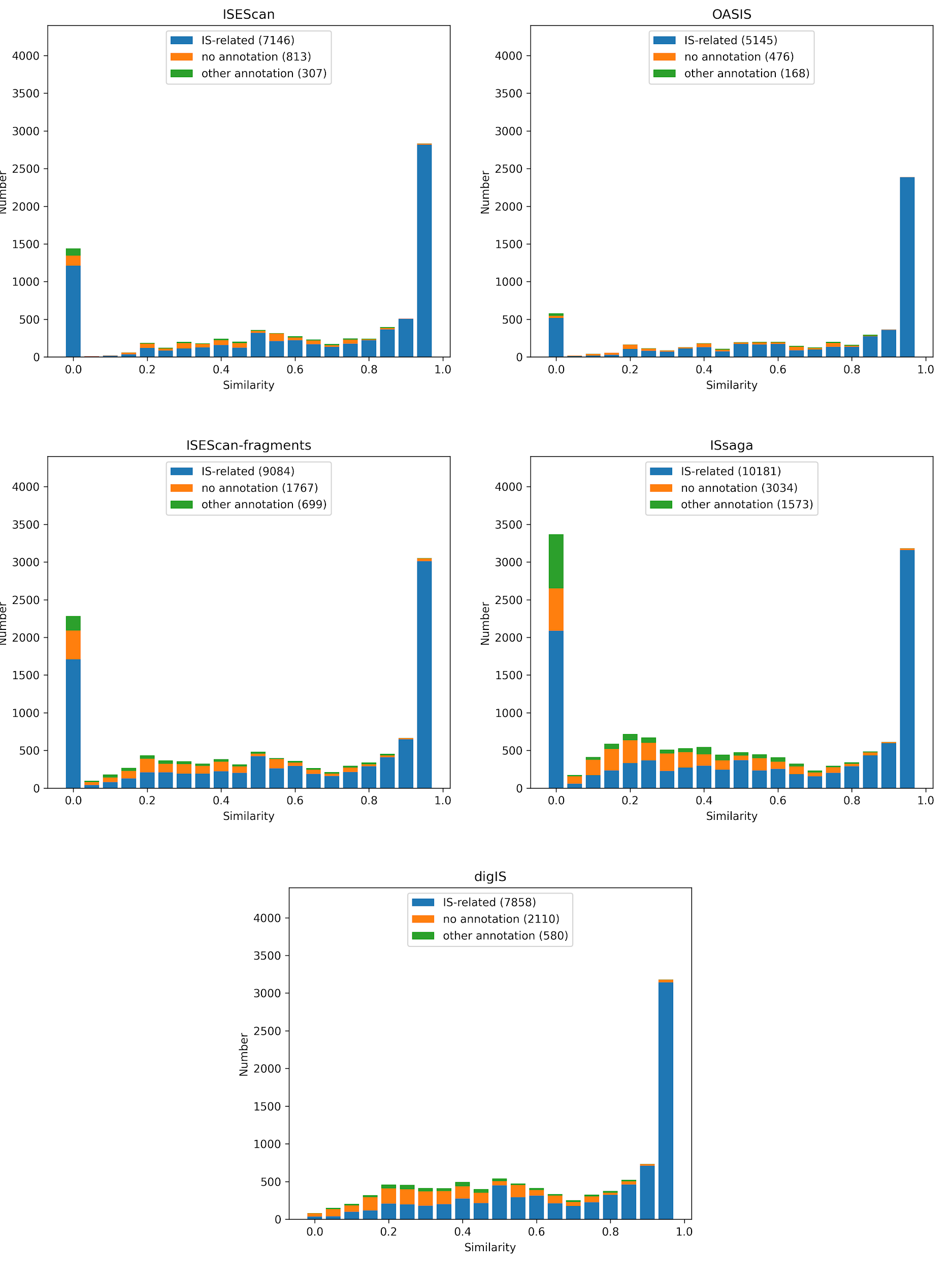


Figure 5A: NCBI Archaea dataset without reference -- Histogram depicting the number of mFPs as a function of their similarity to the ISfinder database (at ORF level) and their classification according to the GenBank annotation.


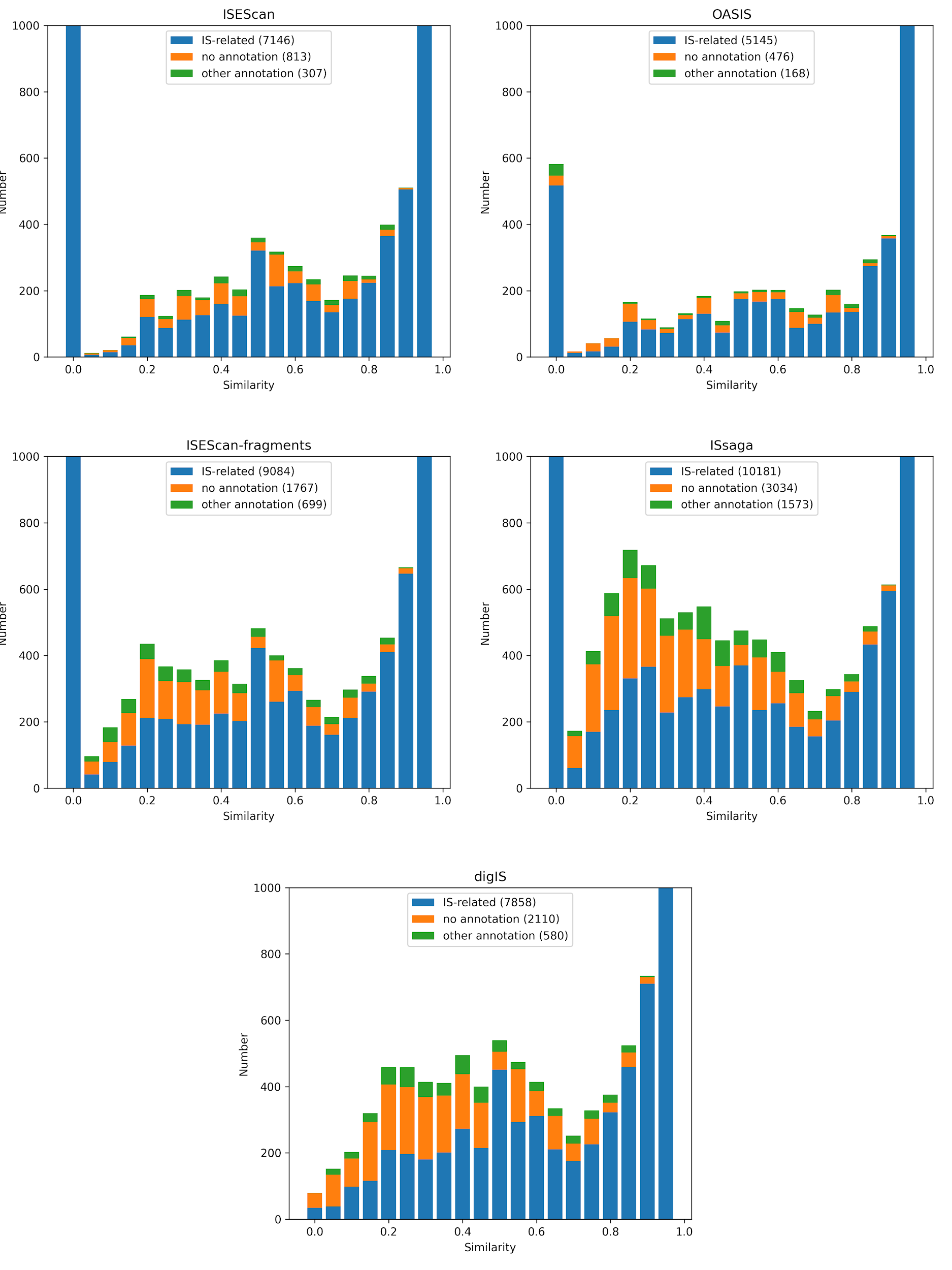


Figure 5B: NCBI Archaea dataset without reference -- Enlarged view of the histogram depicting the number of mFPs as a function of their similarity to the ISfinder database (at ORF level) and their classification according to the GenBank annotation.


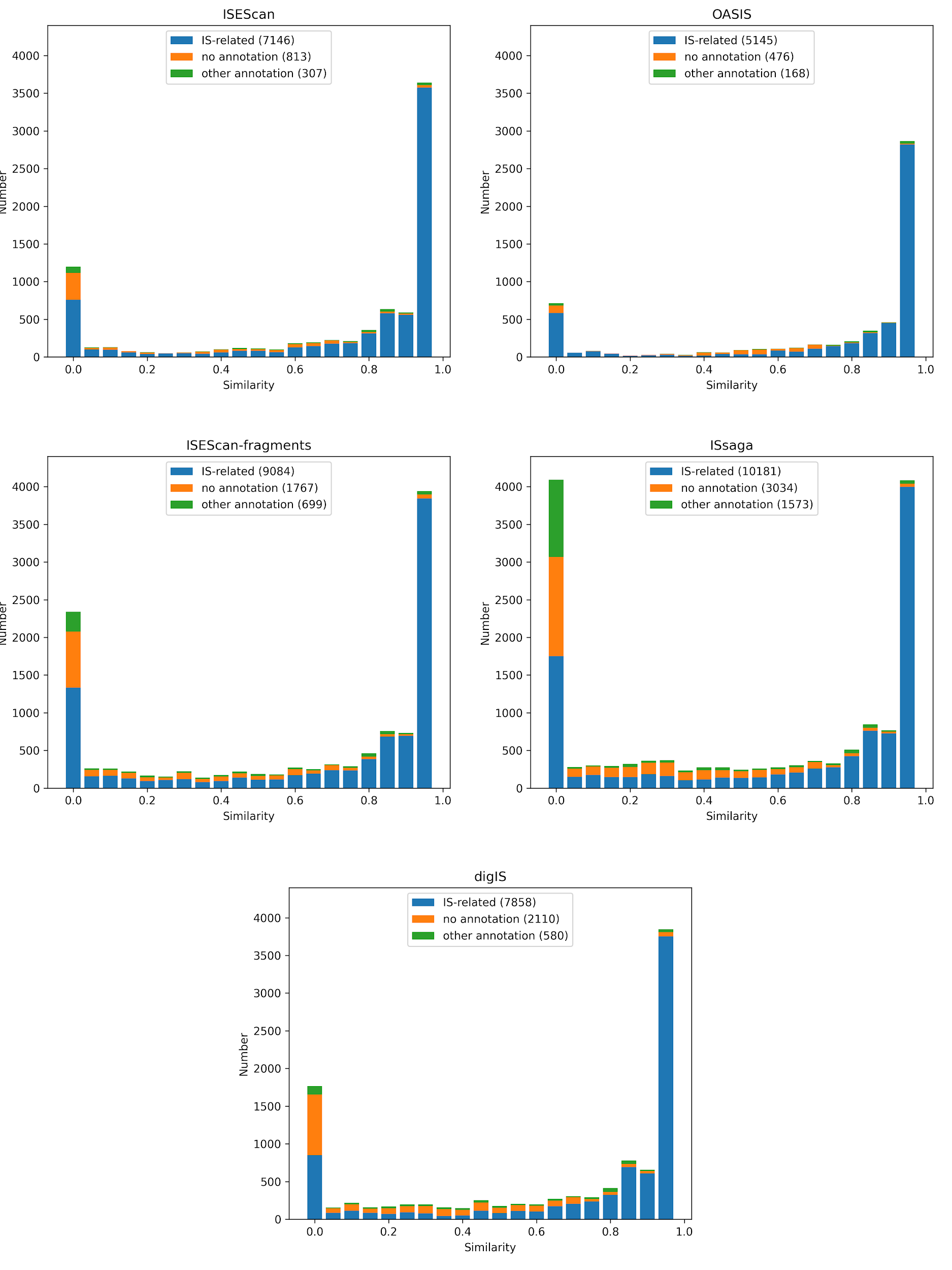


Figure 6A: NCBI Archaea dataset without reference -- Histogram depicting the number of mFPs as a function of their similarity to the ISfinder database (at DNA level) and their classification according to the GenBank annotation.


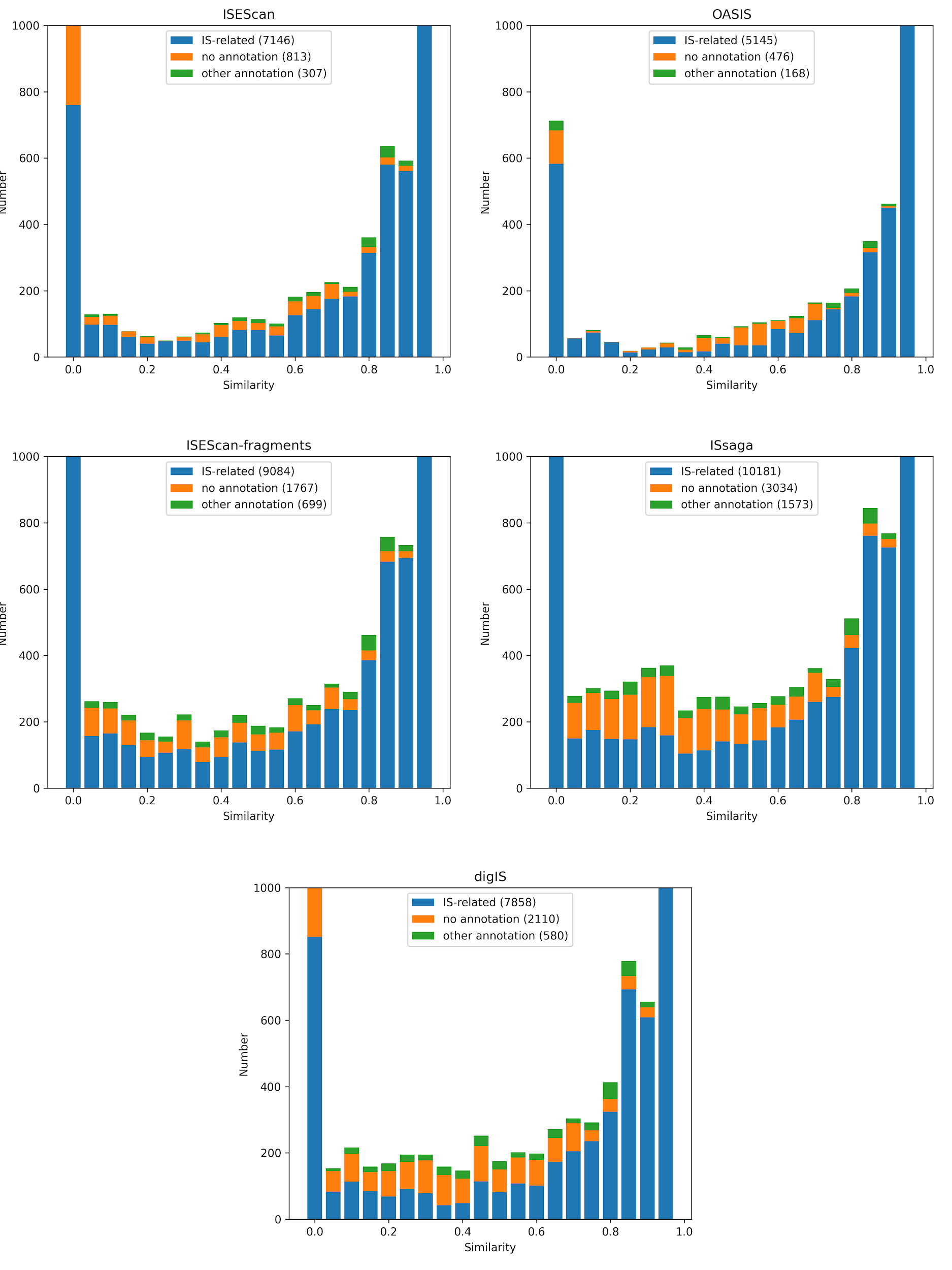


Figure 6B: NCBI Archaea dataset without reference -- Enlarged view of the histogram depicting the number of mFPs as a function of their similarity to the ISfinder database (at DNA level) and their classification according to the GenBank annotation.


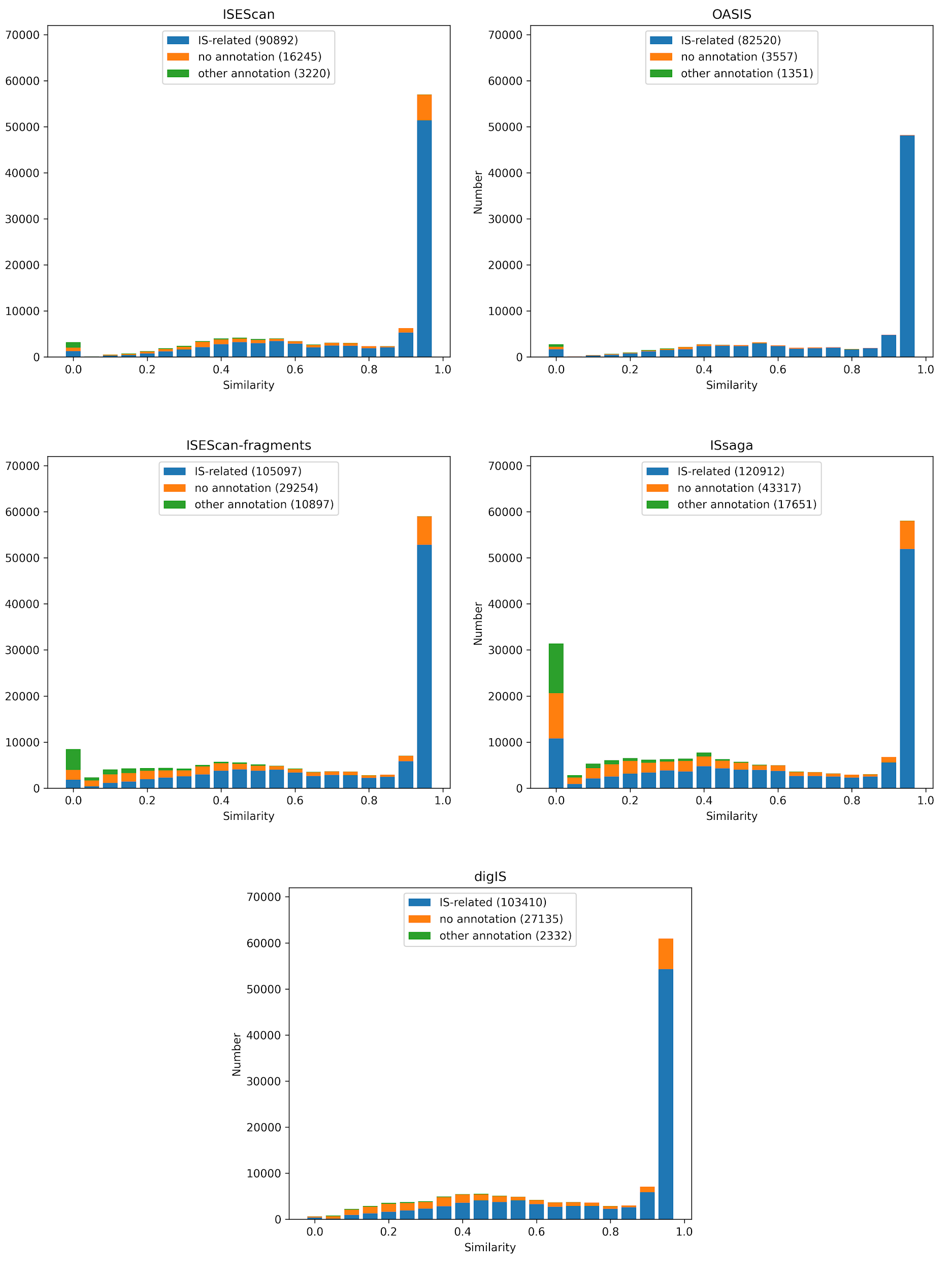


Figure 7A: NCBI Bacteria dataset without reference -- Histogram depicting the number of mFPs as a function of their similarity to the ISfinder database (at ORF level) and their classification according to the GenBank annotation.


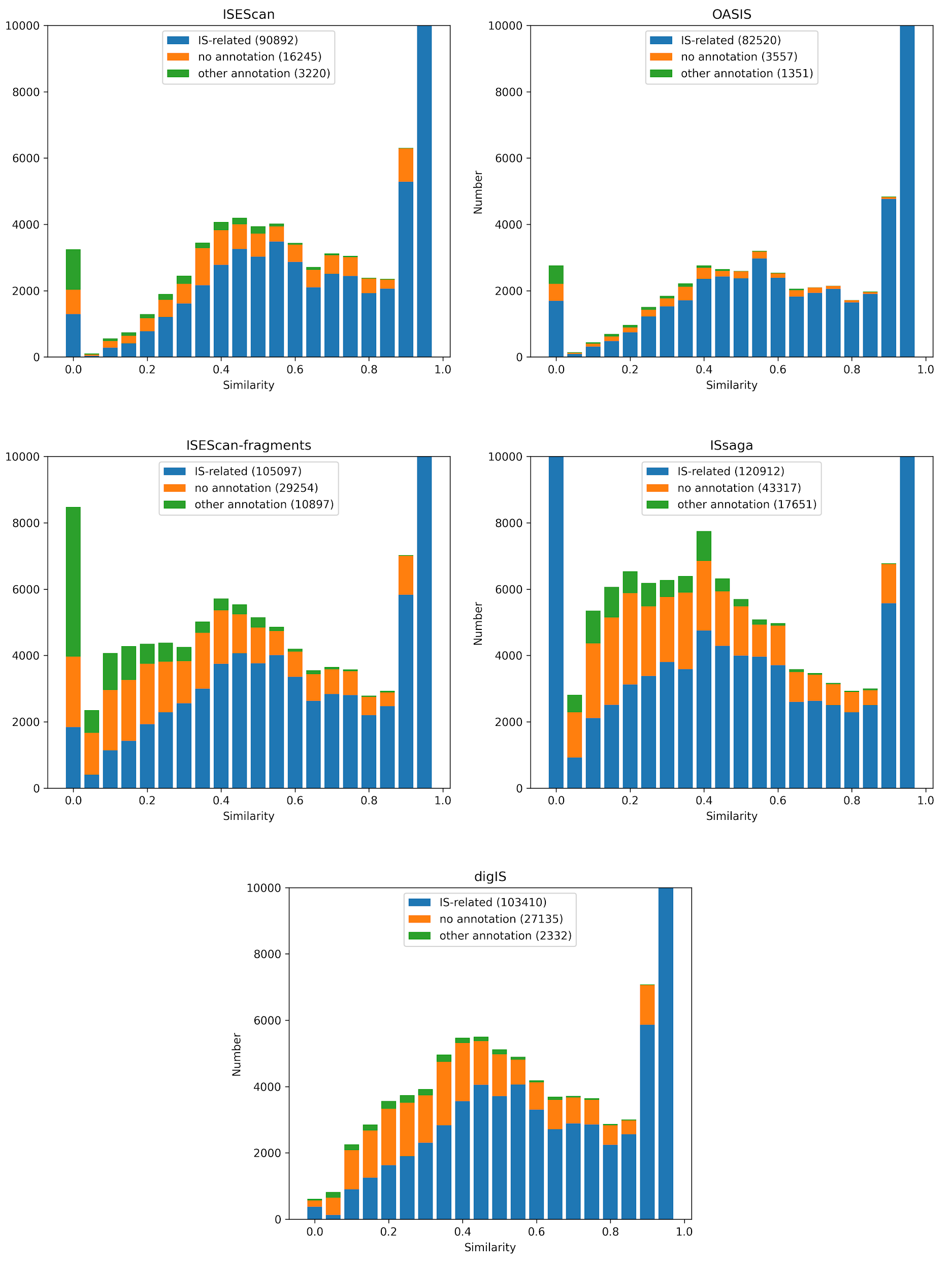


Figure 7B: NCBI Bacteria dataset without reference -- Enlarged view of the histogram depicting the number of mFPs as a function of their similarity to the ISfinder database (at ORF level) and their classification according to the GenBank annotation.


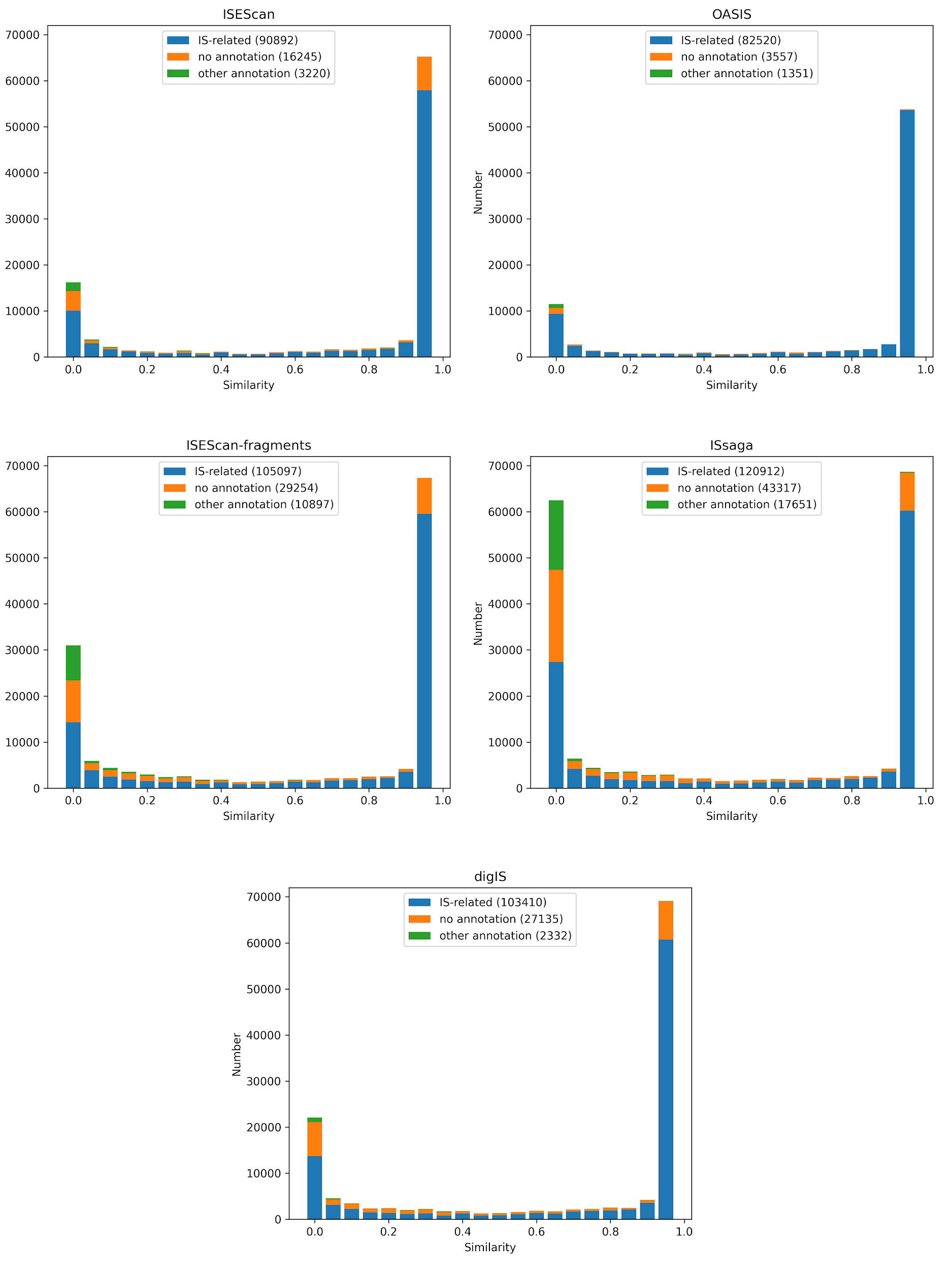


Figure 8A: NCBI Bacteria dataset without reference -- Histogram depicting the number of mFPs as a function of their similarity to the ISfinder database (at DNA level) and their classification according to the GenBank annotation.


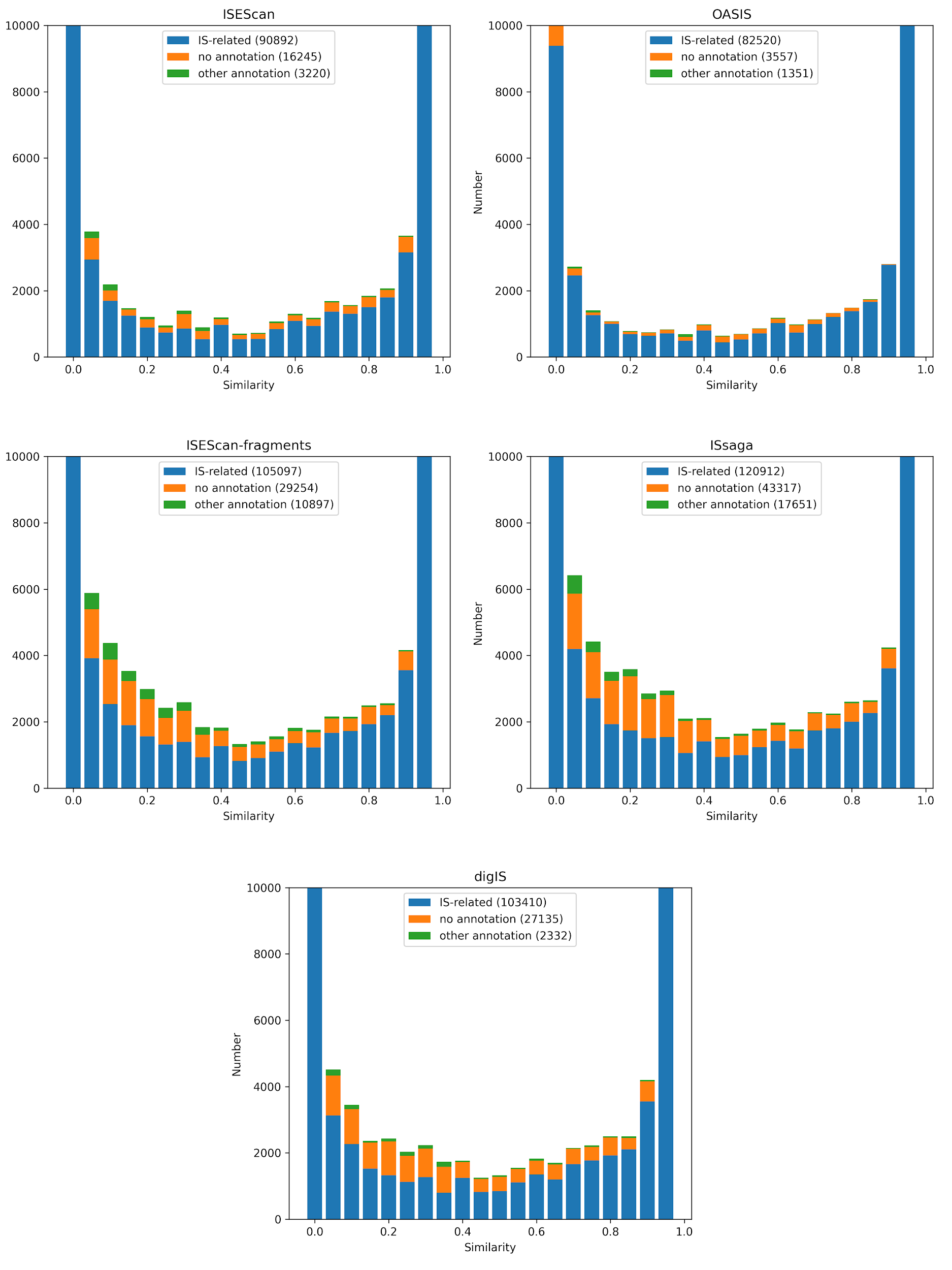


Figure 8B: NCBI Bacteria dataset without reference -- Enlarged view of the histogram depicting the number of mFPs as a function of their similarity to the ISfinder database (at DNA level) and their classification according to the GenBank annotation.

1. More than 90% identity, measured according to the length of the item in the database. [↑](#footnote-ref-0)
2. Less than 10% identity, measured according to the length of the item in the database. [↑](#footnote-ref-1)
