# Supplementary material for: digIS: towards detecting distant and putative novel insertion sequence elements in prokaryotic genomes
Source: BMC Bioinformatics. 2021 May 20;22:258. doi: 10.1186/s12859-021-04177-6 (PMC8147514; doi:10.1186/s12859-021-04177-6)
Supplement: Supplementary file 5 — Additional file 5. Calculation of the similarity at IS and ORF level. [file 12859_2021_4177_MOESM5_ESM.docx]

# Calculation of the similarity at ORF and DNA level

The threshold for *Intra-family member* similarity was calculated as follows:

1. For each IS family, sequences of full-length IS elements were extracted from the ISfinder database.
2. Sequence similarity between every two members inside the IS family was measured (see solid lines in Figure 1), and an average similarity was calculated for the whole IS family.
3. The final threshold was calculated as a mean from these values across all IS families.
4. The same procedure was repeated for Tpase ORFs at the amino acid level.

In other words, the outputs with similarity above this threshold can be interpreted as members of some known IS family (intra-family similarity).

Lower threshold for *Inter-family member* similarity was calculated as follows:

1. For each IS family, the centroid sequence was identified as a sequence with the shortest distance to all other family sequences (see circled points in Figure 1), and
2. The similarity between every two centroid sequences was measured, and the final threshold was calculated as a mean from all these values.

In other words, the outputs with similarity above this threshold can be interpreted as IS distant homologs, as they have a similarity usually seen between IS families (inter-family similarity).

Finally, classification for the IS element and Tpase/ORF is combined into the overall similarity level as follows:

- *Intra-family member* - either the IS element or Tpase/ORF reached intra-family member similarity,
- *Inter-family member* - either the IS element or Tpase/ORF reached inter-family member similarity,
- *Improbable member* - otherwise.

In other words, the outputs are classified according to the higher level from both similarities.

Calculated values of intra and inter-family similarities for individual families are listed in Table 1.


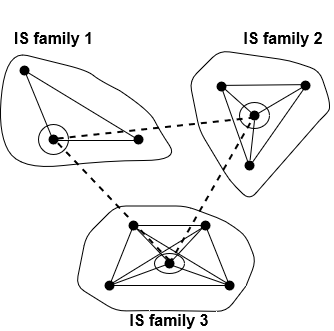


**Figure 1.** Calculation of similarity thresholds. For each IS family, the intra-family sequence similarity is measured as an average distance between every two members (solid lines), and the overall intra-family similarity threshold is calculated as a mean of these values across all families. Then, the centroid sequences (with the shortest distance to other members) are selected as family representatives (circled points) and the inter-family similarity threshold is calculated as an average distance between every two representatives (dashed lines).

| **Family** | **Intra-family (DNA)** | **Inter-family (DNA)** | **Intra-family  (ORF)** | **Inter-family  (ORF)** |
| --- | --- | --- | --- | --- |
| IS1 | 0.68 | 0.49 | 0.45 | 0.24 |
| IS110 | 0.67 | 0.46 | 0.35 | 0.23 |
| IS1182 | 0.66 | 0.53 | 0.30 | 0.26 |
| IS1380 | 0.66 | 0.52 | 0.31 | 0.26 |
| IS1595 | 0.65 | 0.54 | 0.32 | 0.26 |
| IS1634 | 0.64 | 0.40 | 0.31 | 0.22 |
| IS200_IS605 | 0.67 | 0.50 | 0.39 | 0.24 |
| IS21 | 0.70 | 0.51 | 0.39 | 0.25 |
| IS256 | 0.68 | 0.53 | 0.38 | 0.27 |
| IS3 | 0.69 | 0.51 | 0.40 | 0.26 |
| IS30 | 0.69 | 0.50 | 0.45 | 0.25 |
| IS3_IS150 | 0.69 | 0.51 | 0.44 | 0.25 |
| IS3_IS2 | 0.78 | 0.53 | 0.69 | 0.25 |
| IS3_IS3 | 0.71 | 0.51 | 0.52 | 0.26 |
| IS3_IS407 | 0.74 | 0.51 | 0.53 | 0.25 |
| IS3_IS51 | 0.73 | 0.51 | 0.57 | 0.26 |
| IS4 | 0.64 | 0.52 | 0.31 | 0.24 |
| IS481 | 0.66 | 0.54 | 0.37 | 0.27 |
| IS4_IS10 | 0.64 | 0.50 | 0.32 | 0.26 |
| IS4_IS231 | 0.73 | 0.45 | 0.55 | 0.25 |
| IS4_IS4 | 0.69 | 0.52 | 0.38 | 0.26 |
| IS4_IS4Sa | 0.67 | 0.53 | 0.37 | 0.26 |
| IS4_IS50 | 0.67 | 0.44 | 0.40 | 0.24 |
| IS4_ISH8 | 0.70 | 0.52 | 0.48 | 0.26 |
| IS4_ISPepr1 | 0.66 | 0.49 | 0.36 | 0.25 |
| IS5_IS1031 | 0.73 | 0.53 | 0.52 | 0.27 |
| IS5_IS427 | 0.66 | 0.53 | 0.36 | 0.28 |
| IS5_IS5 | 0.78 | 0.55 | 0.69 | 0.29 |
| IS5_IS903 | 0.71 | 0.54 | 0.53 | 0.27 |
| IS5_ISH1 | 0.75 | 0.54 | 0.62 | 0.27 |
| IS5_ISL2 | 0.67 | 0.54 | 0.39 | 0.27 |
| IS5_None | 0.74 | 0.53 | 0.59 | 0.27 |
| IS6 | 0.69 | 0.52 | 0.45 | 0.27 |
| IS607 | 0.68 | 0.45 | 0.49 | 0.22 |
| IS630 | 0.66 | 0.54 | 0.30 | 0.26 |
| IS66 | 0.67 | 0.52 | 0.35 | 0.26 |
| IS701 | 0.63 | 0.54 | 0.30 | 0.27 |
| IS91 | 0.66 | 0.53 | 0.34 | 0.25 |
| IS982 | 0.69 | 0.52 | 0.40 | 0.27 |
| ISAs1 | 0.68 | 0.52 | 0.42 | 0.26 |
| ISAzo13 | 0.74 | 0.54 | 0.69 | 0.27 |
| ISH3 | 0.75 | 0.53 | 0.49 | 0.26 |
| ISH6 | 0.85 | 0.54 | 0.78 | 0.26 |
| ISKra4 | 0.65 | 0.51 | 0.30 | 0.26 |
| ISL3 | 0.64 | 0.49 | 0.31 | 0.24 |
| ISLre2 | 0.68 | 0.51 | 0.31 | 0.26 |
| Tn3 | 0.72 | 0.50 | 0.49 | 0.26 |
| **Mean** | **0.69** | **0.51** | **0.44** | **0.26** |

**Table 1.** Intra- and inter-family similarities calculated for individual families at DNA and ORF levels. The last row represents mean values used as thresholds for output record classification based on similarity with the ISfinder database.
