# Supplementary material for: digIS: towards detecting distant and putative novel insertion sequence elements in prokaryotic genomes
Source: BMC Bioinformatics. 2021 May 20;22:258. doi: 10.1186/s12859-021-04177-6 (PMC8147514; doi:10.1186/s12859-021-04177-6)
Supplement: Supplementary file 6 — Additional file 6. Detailed information about NCBI GenBank archaeal and bacterial genomes used in the evaluation. [file 12859_2021_4177_MOESM6_ESM.docx]

Complete assemblies of bacterial and archaeal genomes and their GenBank annotations were downloaded from the NCBI GenBank FTP site on June 15, 2019. A list of links of archaeal genomes used in the evaluation is available at <https://github.com/janka2012/digIS/blob/master/evaluation/archaea_complete_genomic_files.txt>, and a list of links of bacterial genomes used in the evaluation is available at <https://github.com/janka2012/digIS/blob/master/evaluation/bacteria_complete_genomic_files.txt>. Altogether, 341 complete archaeal genomes containing 527 sequences and 2,500 complete bacterial genomes containing 5,018 sequences were used for the assessment.

The genomes were downloaded and filtered using the following commands:

# download NCBI GenBank bacterial genomes

wget ftp://ftp.ncbi.nlm.nih.gov/genomes/genbank/bacteria/assembly_summary.txt -O archaea_assembly_summary.txt

wget ftp://ftp.ncbi.nlm.nih.gov/genomes/genbank/bacteria/assembly_summary.txt -O bacteria_assembly_summary.txt

# filter only Complete Genome assemblies

less archaea_assembly_summary.txt | awk -F '\t' '{if($12=="Complete Genome") print $20}' archaea_assembly_summary.txt > archaea_assembly_summary_complete_genomes.txt

less bacteria_assembly_summary.txt | awk -F '\t' '{if($12=="Complete Genome") print $20}' bacteria_assembly_summary.txt > bacteria_assembly_summary_complete_genomes.txt

# Generate links for downloading the genomes

less archaea_assembly_summary_complete_genomes.txt | sed -r 's|(ftp://ftp.ncbi.nlm.nih.gov/genomes/all/.+/)(GCA_.+)|\1\2/\2_genomic.fna.gz|' > archaea_complete_genomic_files.txt

less bacteria_assembly_summary_complete_genomes.txt | sed -r 's|(ftp://ftp.ncbi.nlm.nih.gov/genomes/all/.+/)(GCA_.+)|\1\2/\2_genomic.fna.gz|' > bacteria_complete_genomic_files.txt

# Random selection of 2500 bacterial genomes

less bacteria_complete_genomic_files.txt | sort -R | head -2500 > bacteria_complete_genomic_files_subset.txt
