# Supplementary material for: digIS: towards detecting distant and putative novel insertion sequence elements in prokaryotic genomes
Source: BMC Bioinformatics. 2021 May 20;22:258. doi: 10.1186/s12859-021-04177-6 (PMC8147514; doi:10.1186/s12859-021-04177-6)
Supplement: Supplementary file 7 — Additional file 7. Analysis of hits classified as other annotation. [file 12859_2021_4177_MOESM7_ESM.docx]

## Analysis of hits classified as “other annotation”

### Goals

- Analyze hits that were incorrectly identified by the tool as IS elements or their parts.
- Focus on hits that were classified as *other annotation* based on the overlap with GenBank records.
- Find out which types of GenBank records are often a source of error for a given tool.

### Procedure

1. Hits classified as *other annotation* were extracted from the outputs of each tool.
2. For each hit, all GenBank records having an overlap of at least 100 bp with it were found.
3. From the found GenBank records, all strings of the product attribute were extracted, if available.
4. A histogram of these strings was created.
5. Strings that indicated an unknown or hypothetical protein were removed from the histogram.
6. Top histogram items were depicted.

### Notes

- The number of hits classified as *other annotation* may not match the number of items displayed in the histogram because:
  - The histogram shows only the top N items.
  - Some GenBank records do not have a product attribute (e.g., some types of pseudogenes).
  - Some hits have an overlap with multiple GenBank records, and a single GenBank record may refer to numerous products.

##

### ISbrowser dataset (without reference)

| **ISsaga** | **ISEScan-fragments** | **digIS** | **ISEScan** | **OASIS** |
| --- | --- | --- | --- | --- |
| 74 | 67 | 7 | 9 | 0 |

Table 1: Number of hits classified as *other annotations*.

###
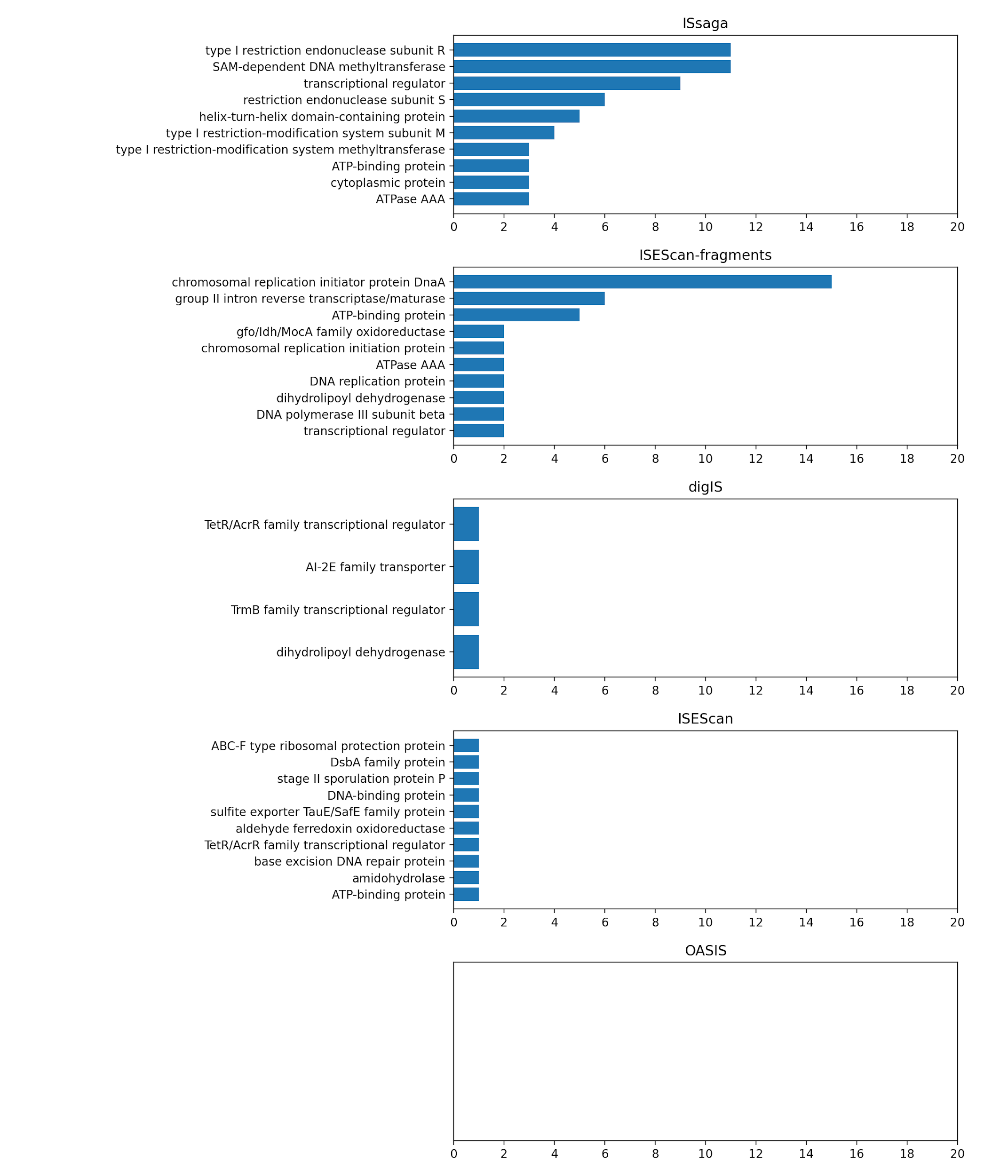


Figure 1: Histogram of product attributes of GenBank records that overlapped with hits classified as *other annotations*.

### NCBI Archaea dataset (without reference)

| **ISsaga** | **ISEScan-fragments** | **digIS** | **ISEScan** | **OASIS** |
| --- | --- | --- | --- | --- |
| 1573 | 699 | 580 | 307 | 168 |

Table 2: Number of hits classified as *other annotations*.


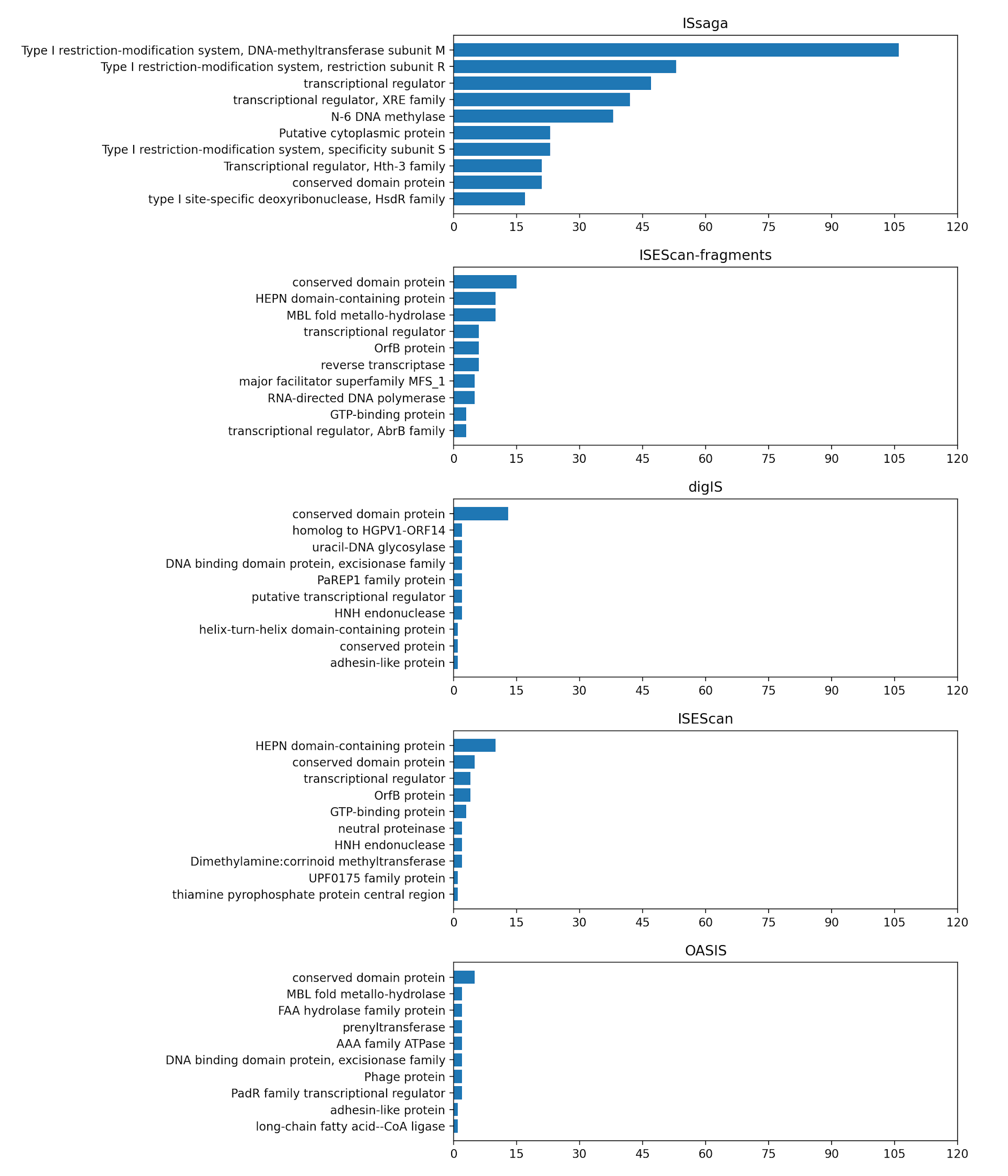


Figure 2: Histogram of product attributes of GenBank records that overlapped with hits classified as *other annotations*.

### NCBI Bacteria dataset (without reference)

| **ISsaga** | **ISEScan-fragments** | **digIS** | **ISEScan** | **OASIS** |
| --- | --- | --- | --- | --- |
| 17651 | 10897 | 2332 | 3220 | 1351 |

Table 3: Number of hits classified as *other annotations*.


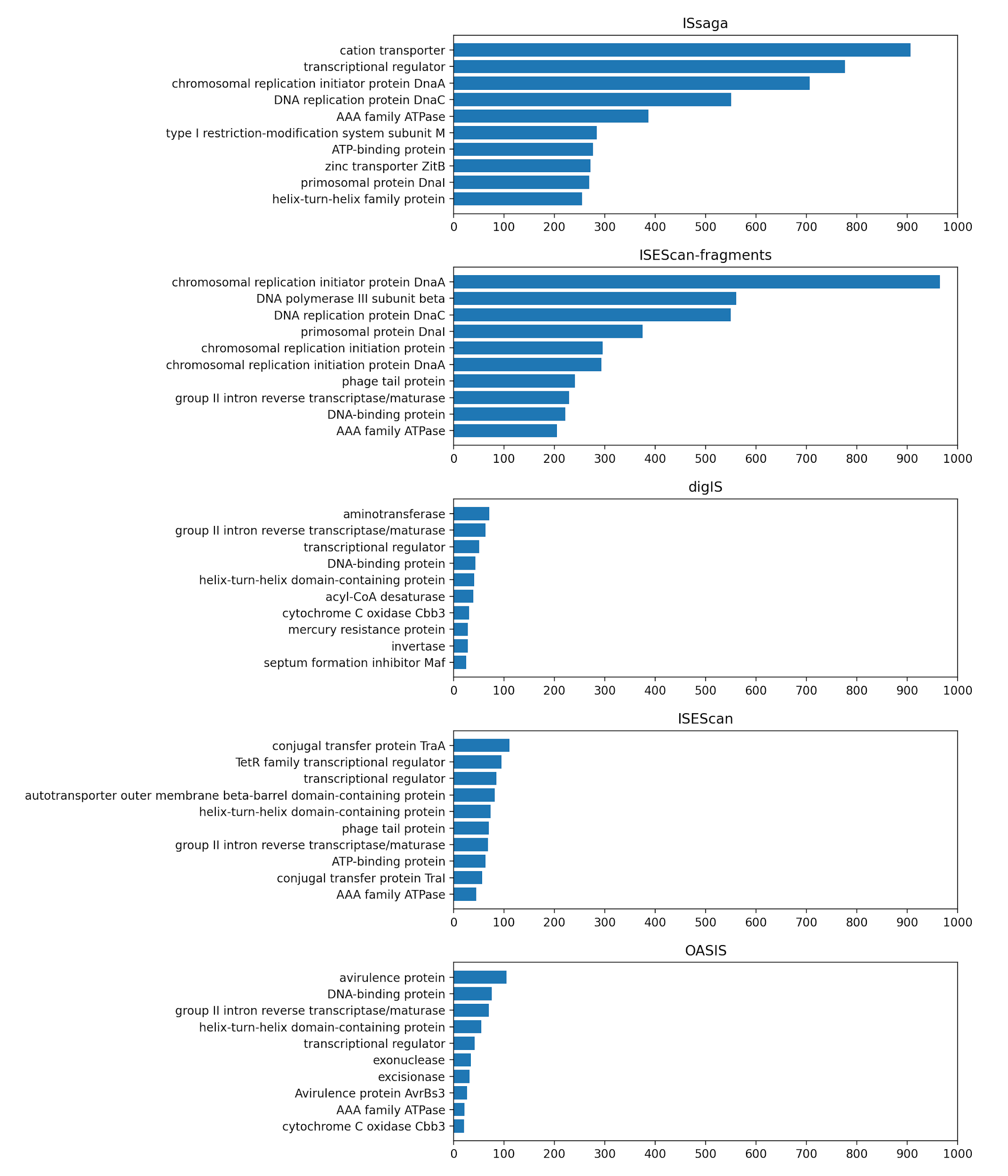


Figure 3: Histogram of product attributes of GenBank records that overlapped with hits classified as *other annotations*.

## Analysis of the most frequent hits classified as “other annotation” reported exclusively by fragment-reporting tools

### Goal

- Analyze the hits classified as “other annotation” that are reported by fragment-reporting tools (ISsaga and ISEScan-fragments) only. These products include: *chromosomal replication initiator protein DnaA*, *DNA replication protein DnaC*, or *primosomal protein DnaI*.
- Find out which parts of these products share a similarity with either transposases or other ORFs carried by IS elements.

### Procedure

1. Hits classified as *other annotation* with products i) chromosomal replication initiator protein DnaA, ii) DNA replication protein DnaC, and iii) primosomal protein DnaI were extracted from the outputs of ISsaga and ISEScan-fragments tool.
2. Hits were examined to identify the IS family and the ISfinder database best hit.
3. Sequences of the best hits with ISfinder database (ORF level) and representative protein sequences of a given product selected randomly were aligned by Clustal Omega and visualized in JalView v2.10.5.
4. Conserved regions were highlighted in red rectangles.

### Findings

- Analyzed proteins (*chromosomal replication initiator protein DnaA*, *DNA replication protein DnaC*, or *primosomal protein DnaI*) share conserved regions having significant sequence similarity with coding regions of IS elements belonging to the IS21 family.

####

#### Chromosomal replication initiator protein DnaA


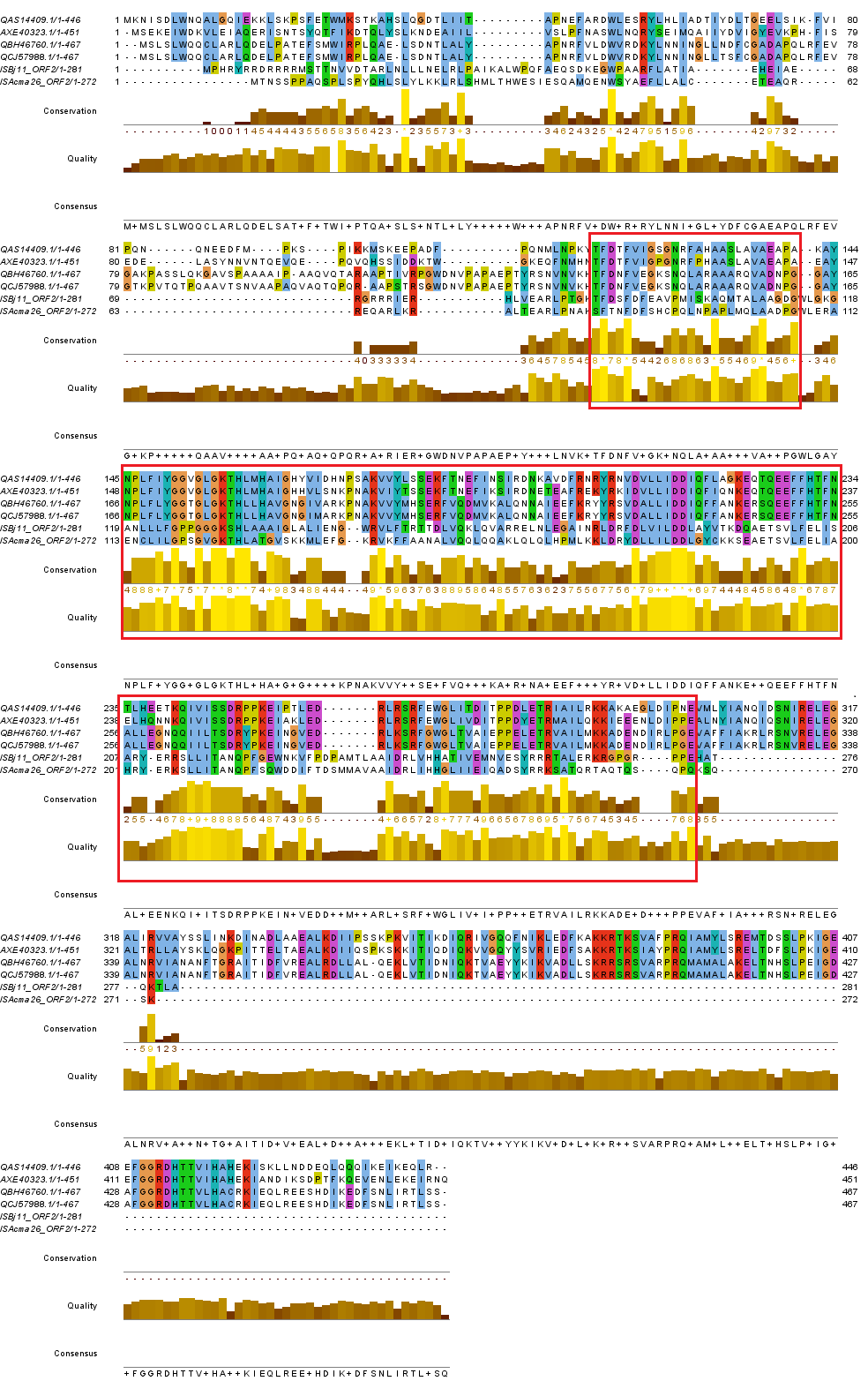


Figure 4. MSA of the best hits with the ISfinder database (ISBj11 ORF2, ISAcma26 ORF2) and randomly selected protein sequences annotated as *chromosomal replication initiator protein DnaA* (QAS14409.1, AXE40323.1, QBH46760.1, QCJ57988.1). Conserved regions are highlighted in red rectangles.

#### DNA replication protein DnaC


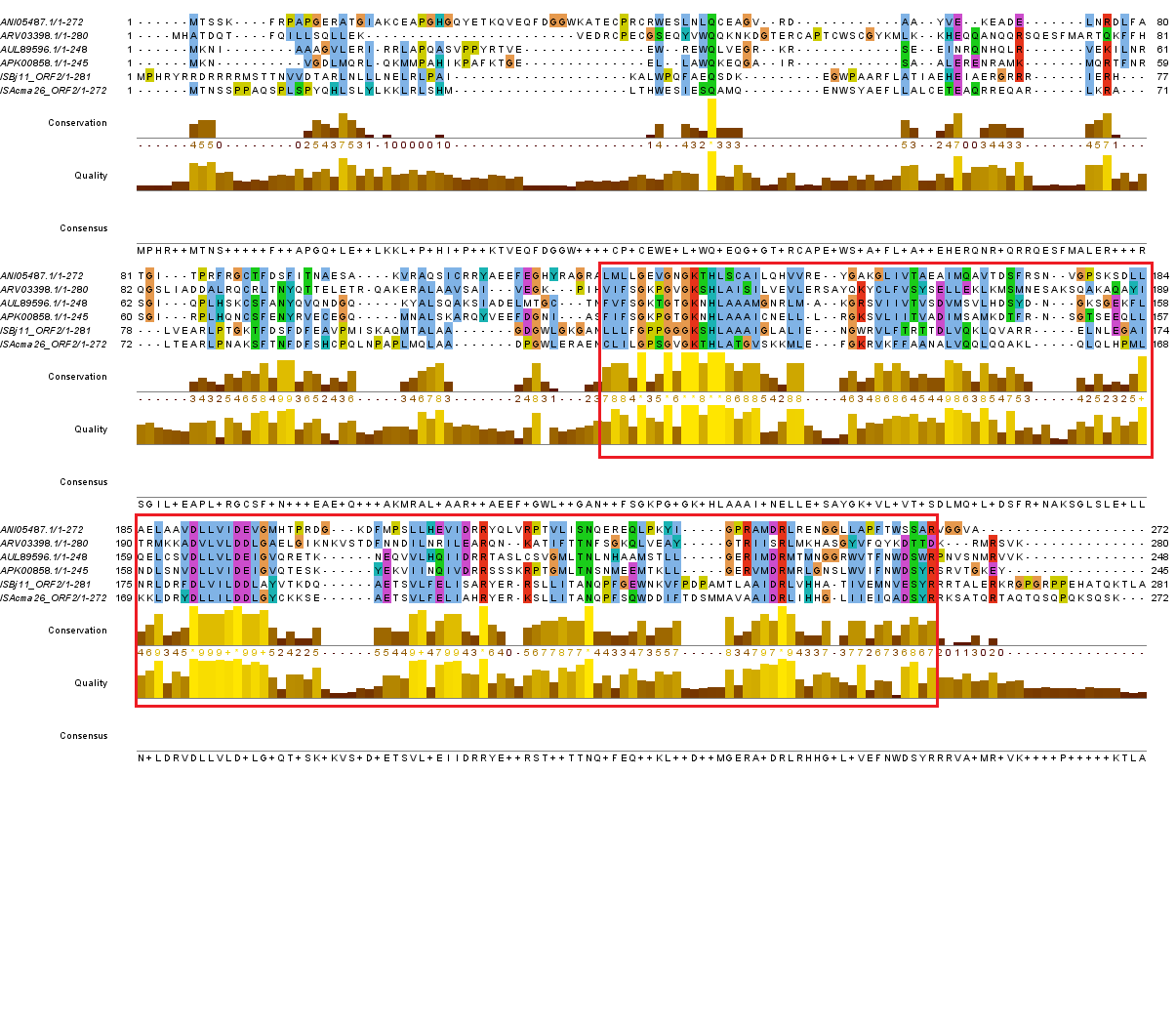


Figure 5. MSA of the best hits with the ISfinder database (ISBj11 ORF2, ISAcma26 ORF2) and randomly selected protein sequences annotated as *DNA replication protein DnaC* (ANI05487.1, ARV03398.1, AUL89596.1, APK00858.1). Conserved regions are highlighted in red rectangles.

####

#### Primosomal protein DnaI


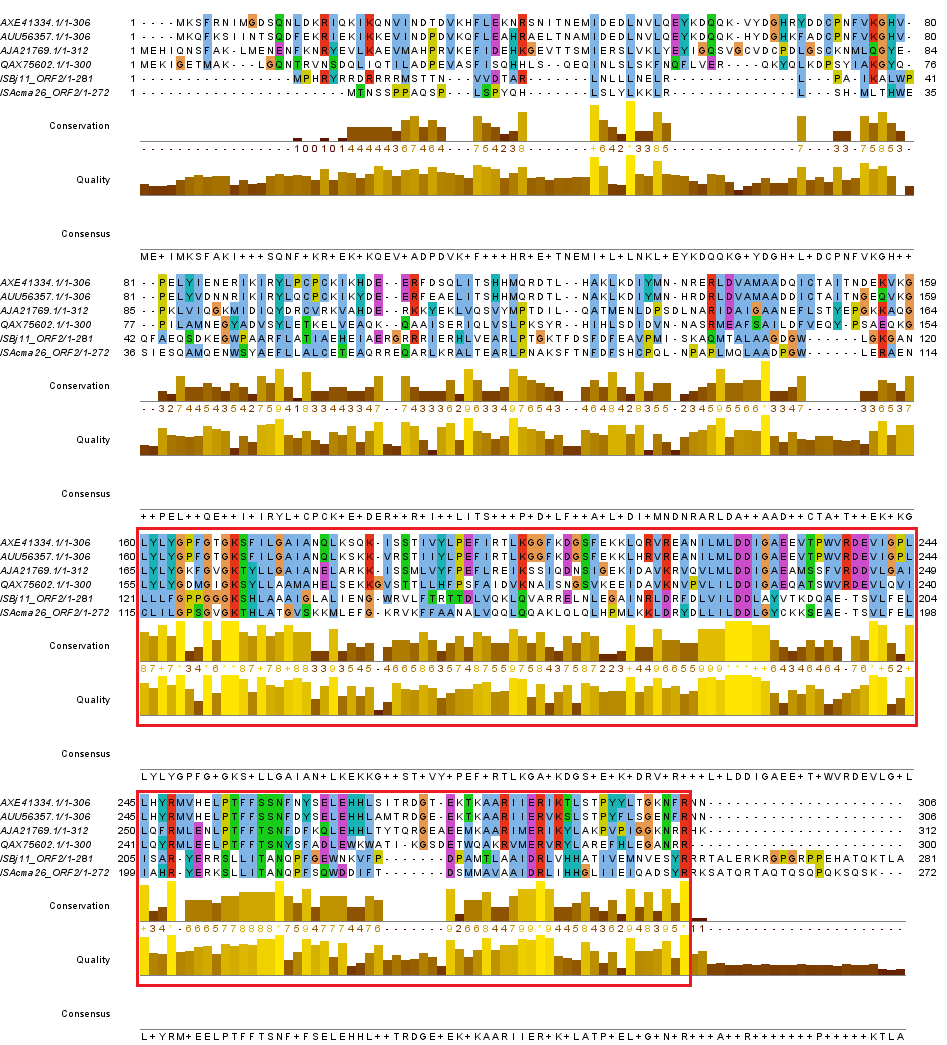


Figure 6. MSA of the best hits with the ISfinder database (ISBj11 ORF2, ISAcma26 ORF2) and randomly selected protein sequences annotated as *DNA replication protein DnaC* (AXE41334.1, AUU56357.1, AJA21769.1, QAX75602.1). Conserved regions are highlighted in red rectangles.
