# Supplementary material for: digIS: towards detecting distant and putative novel insertion sequence elements in prokaryotic genomes
Source: BMC Bioinformatics. 2021 May 20;22:258. doi: 10.1186/s12859-021-04177-6 (PMC8147514; doi:10.1186/s12859-021-04177-6)
Supplement: Supplementary file 8 — Additional file 8. Analysis of putative novel elements. [file 12859_2021_4177_MOESM8_ESM.docx]

# Analysis of putative novel elements

## Coverage of putative novel elements reported by tools and their proximity to the known IS families

### Motivation

In order to assess whether *digIS* was able to detect different putative novel elements compared to other tools or in general to determine variability of pNovs across different tools, a detailed analysis of the coverage of pNovs reported by tools was performed.

### Procedure

A Venn diagram is commonly used for this type of analysis. However, when it is applied to five or more groups, it becomes unclear. Therefore, a matrix showing all-against-all coverage of pNovs was created for both the NCBI Archaea and Bacteria datasets (see Figure 1 and 2). The rows and columns of the matrix represent the individual tools. The numbers on the diagonal correspond to the numbers of pNovs found by the tools. The intersection of a row and a column shows the number of pNovs that were identically found by the corresponding pair of tools^^[[1]](#footnote-0)^^. The ISbrowser and *E.coli* datasets were excluded from this analysis, as they contain too few pNov outputs and their coverage matrices would not be informative.

It turned out that the all-against-all coverage matrices did not provide a satisfactory level of detail. Therefore, the pNovs were further analysed from the perspective of their proximity to existing IS families. During this analysis, each pNov hit was BLASTed against a database of known IS elements (ISfinder) and the best hit was used in order to define its closest IS family. Subsequently, for each tool, a histogram representing the number of pNovs per the closest IS family was plotted for both the NCBI Archaea and Bacteria datasets (see Figures 3 and 4).


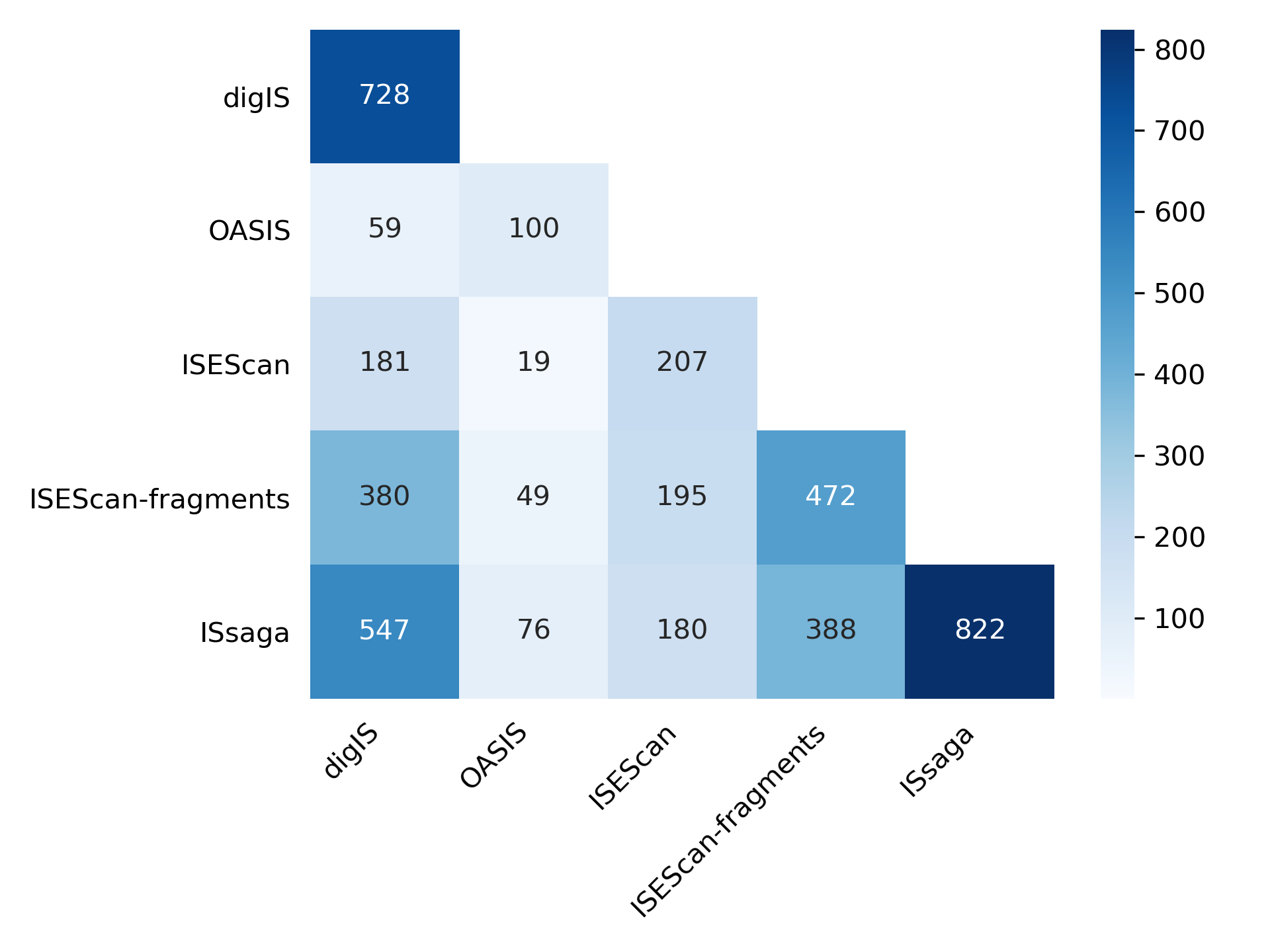


Figure 1: All-against-all coverage matrix for NCBI Archaea dataset.


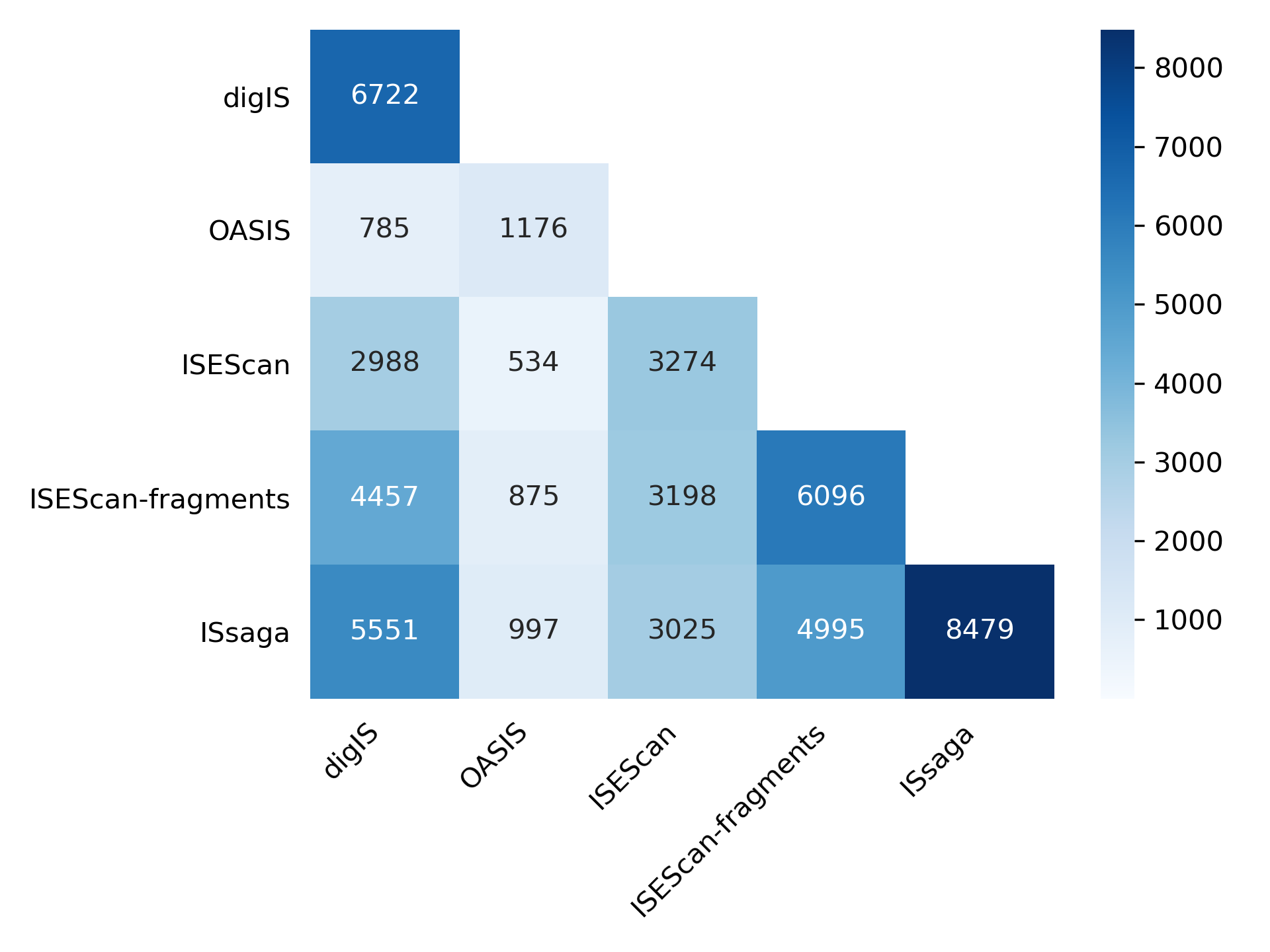


Figure 2: All-against-all coverage matrix for NCBI Bacteria dataset.

### Findings

For the NCBI Archaea dataset (see Figure 3), the following was found :

- None of the tools found any pNov related to IS families: IS1380, IS21, IS3, IS30, IS91, IS982, ISAs1, ISH6, ISKra4, ISLre2, and Tn3. Archaeal genomes lacked elements of the IS1380, ISLre2, ISAs1, and ISKra4 families, and only a few members of IS3, IS1182, IS21, IS91, IS30, and IS982 were present in the archaeal genome, even though they are widespread in bacterial genomes [1,2].
- *digIS* found the same amount or more pNovs as the other tools did for families IS1182, IS1595, IS200/IS605, IS4, IS6, IS607, ISH3, ISL3, and ISNCY. These models contained enough sequences to have a good generalization ability to detect putative novel elements.
- Significantly worse performance, in terms of detecting putative novel elements compared to other tools, was observed for pNovs related to IS families: IS1, IS110, IS256, IS5, and IS630.


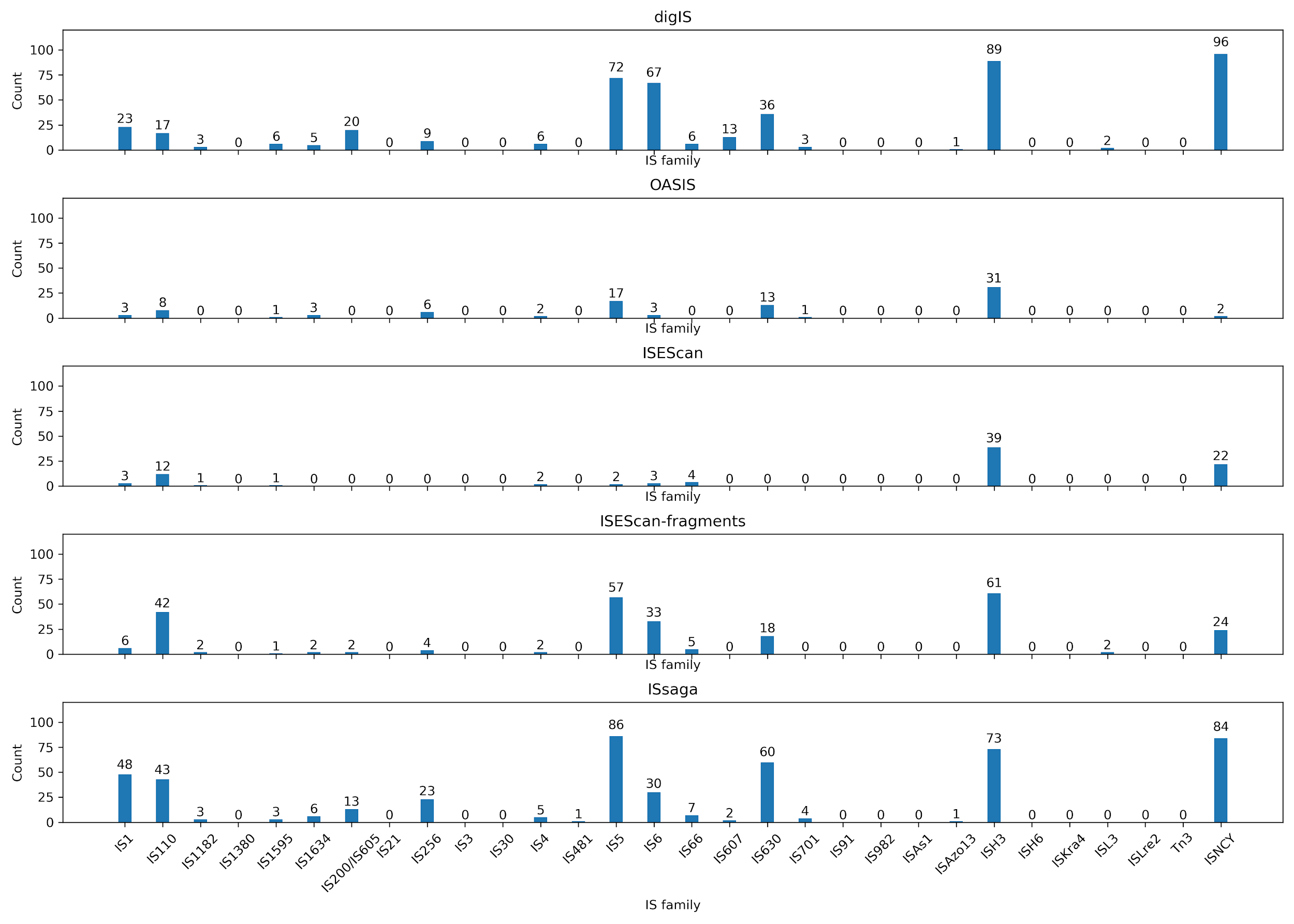


Figure 3: Analysis of putative novel elements per the closest IS family for the NCBI Archaea dataset.

For the NCBI Bacteria dataset (see Figure 4), the following was found :

- None of the tools found any pNov related to IS families ISH3 and ISH6. The IS families were Archaea-specific [3].
- *digIS* found the same amount or more of pNov as the other tools did for families IS1, IS110, IS1595, IS1634, IS200/IS605, IS6, IS607, ISAz1, and ISAzo13.
- Significantly worse performance, in terms of detecting putative novel elements compared to other tools, was observed for pNovs related to IS families IS1182, IS21, IS3, IS30, IS481, IS5, IS66, IS701, and IS91.
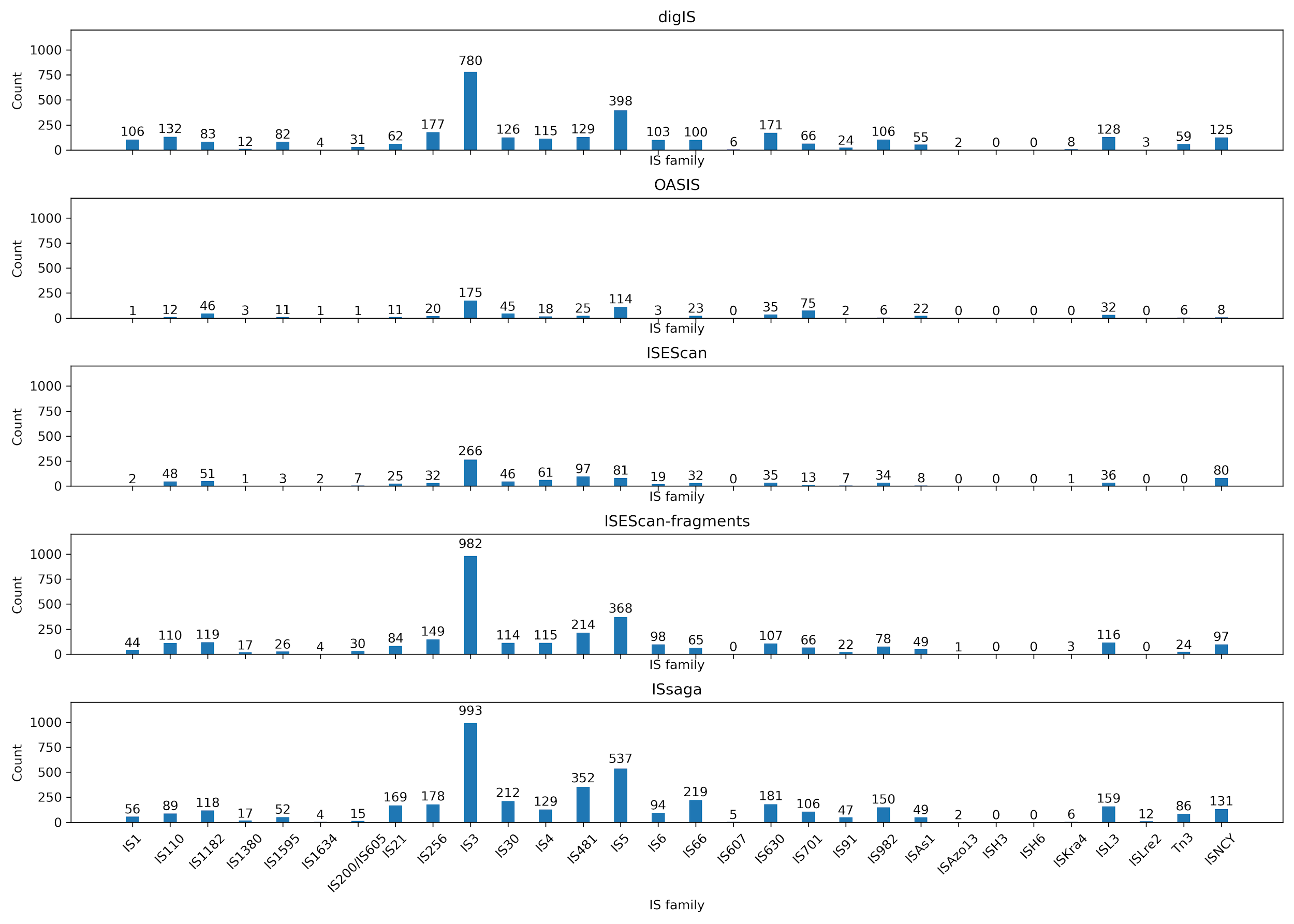


Figure 4: Analysis of putative novel elements per the closest IS family for the NCBI Bacteria dataset.

### Conclusion

It can be seen that various tools have a preference for detecting pNov elements close to various IS families. For example, *digIS* found the most pNovs close to the ISH3 family while ISsaga found the most pNovs close to the IS5 family. Based on these findings, it can be concluded that none of the tools includes all pNov elements of all other tools.

## Alternative definition of putative novel element and its impact on tools’ performance

### Motivation

Currently, a pNov is defined as a hit without a sufficiently specific GenBank annotation and having the sequence similarity that is common among members of different IS families.

Without further restrictions, this category may include, for example, the found accessory genes or some of the transposase's variable domains. To ensure that the found pNov hit is highly likely to be functional and capable of transposition, it is desirable to require the presence of Tpase and its catalytic domain.

The goal of the following experiment is to measure the effect of the alternative definition of pNov requiring the presence of a catalytic domain, and to measure its effect on tool performance.

### Procedure

Firstly, the positions and sequences of catalytic domains were extracted based on manual analysis, see section *Building profile hidden Markov models for the transposase catalytic domain of individual IS families* in the main manuscript for more details. Each pNov was BLASTed against a database of known IS elements (ISfinder) and the overlap between the best reported BLAST hit and positions of catalytic domains was determined. Finally, we counted the hits having an overlap with the catalytic domain greater than the set threshold (50 and 100 bp). The results are available in Tables 1 and 2.

### Findings

When an overlap of a pNov with a catalytic domain was required, *digIS* reported the highest number of pNovs for both the NCBI Archaea and Bacteria datasets on all required overlap thresholds. The number of pNovs reported by ISsaga decreased significantly, which means that ISsaga outputs represent fragments rather than functional IS elements containing a catalytic domain and capable of transposition.

| Tool/Overlap length | without overlap | 50 bp | 100 bp |
| --- | --- | --- | --- |
| OASIS | 100 | 69 | 48 |
| ISEScan | 207 | 195 | 169 |
| ISEScan-fragments | 472 | 404 | 321 |
| ISsaga | 822 | 603 | 412 |
| digIS | 728 | 705 | 489 |

Table 1: Number of pNovs in the NCBI Archaea dataset containing a catalytic domain.

| Tool/Overlap length | without overlap | 50 bp | 100 bp |
| --- | --- | --- | --- |
| OASIS | 1176 | 975 | 847 |
| ISEScan | 3274 | 3120 | 2917 |
| ISEScan-fragments | 6096 | 4994 | 4168 |
| ISsaga | 8479 | 6458 | 5236 |
| digIS | 6722 | 6638 | 5453 |

Table 2: Number of pNovs in the NCBI Bacteria dataset containing a catalytic domain.

### Conclusion

Based on the results in Tables 1 and 2, it can be concluded that other tools also detect a significant portion of pNovs without the catalytic domain. If the presence of the catalytic domain were required in each pNov, then *digIS* would achieve the best results in the detection of pNovs on an absolute scale for both the NCBI Archaea and Bacteria datasets.

### Literature

1. Filée, J., Siguier, P., & Chandler, M. (2007). Insertion sequence diversity in archaea. Microbiology and Molecular Biology Reviews : MMBR, 71(1), 121—157. https://doi.org/10.1128/mmbr.00031-06
2. Nicolas, E., Lambin, M., Dandoy, D., Galloy, C., Nguyen, N., Oger, C. A., & Hallet, B. (2015). The Tn3-family of Replicative Transposons. In Mobile DNA III (pp. 693–726). Retrieved from https://www.asmscience.org/content/book/10.1128/9781555819217.chap32
3. Siguier, P., Gourbeyre, E., Varani, A., Ton-Hoang, B., & Chandler, M. (2015). Everyman’s Guide to Bacterial Insertion Sequences. Mobile DNA III, 555–590. https://doi.org/10.1128/9781555819217.ch26

1. pNov is considered found by both tools if the tools’ hits have an overlap of at least 100 bp. [↑](#footnote-ref-0)
