# Supplementary material for: digIS: towards detecting distant and putative novel insertion sequence elements in prokaryotic genomes
Source: BMC Bioinformatics. 2021 May 20;22:258. doi: 10.1186/s12859-021-04177-6 (PMC8147514; doi:10.1186/s12859-021-04177-6)
Supplement: Supplementary file 9 — Additional file 9. Putative novel IS elements detected by digIS. [file 12859_2021_4177_MOESM9_ESM.docx]

# Putative novel IS elements detected by *digIS*

####

Table of Contents

[Putative novel IS elements detected by *digIS*](#_k8bo9c9a43da) **1**

[Goals](#_h71dsy37q220) 2

[Procedure](#_i97jtgn8fu5b) 2

[Findings](#_t6yn19czal9q) 2

[LM997411.1 - Methylacidiphilum fumariolicum SolV](#_38wtyntm3hi4) 3

[Report for putative novel IS element *pnov1*](#_wmdwodjvj16s) 3

[Report for putative novel IS element *pnov2*](#_rslhwxtuwdlv) 8

[Report for putative novel IS element *pnov3*](#_kodl0ybinbd0) 13

[CP017599.1 - Moorea producens PAL-8-15-08-1](#_cco2vfexvinr) 20

[Report for putative novel IS element *pnov4*](#_isqmcniepoi1) 20

[Literature](#_ren8f1s1dymp) 24

##

## Goals

- Demonstrate the ability of the *digIS* tool to detect putative novel IS elements:
  - To show that the found hits meet the basic requirements for IS elements such as multiple occurrences in the genome, the presence of IR and DR regions.
  - To show that the found IS elements are not yet part of the database of known IS elements - ISfinder.
- Demonstrate the ability of the *digIS* tool to find putative novel IS elements that were not detected by competing tools.

## Procedure

1. Nucleotide sequences of pNovs detected by *digIS* were extracted into FASTA format using *bedtools getfasta* [1]*.*
2. Extracted nucleotide sequences were clustered using *cd-hit-est* [2, 3] with sequence identity set to 0.90%. As IS elements can occur in low-copy numbers, the number of sequences required in one cluster was set to at least two.
3. For each cluster:
   1. Sequences belonging to a cluster were extracted and extended by upstream and downstream flanking regions using *bedtools slop* and *bedtools getfasta* [1].
   2. Multiple sequence alignment (MSA) of extended sequences was created by *Clustal Omega* [4] with default settings.
   3. MSA was visualized by JalView v2.10.5 [5], manually inspected and adjusted if needed, e.g. variable ends were cut.
   4. To identify DRs and IRs, a dotplot of the representative sequences was generated using *Geneious prime* [6].
   5. ORFs were predicted by *Geneious prime* [6].
   6. To confirm that the identified IS element is a putative novel IS element, *blastn* of DNA sequence and *blastp* of translated ORFs [7] was performed against the ISfinder database [8] and the resulting hits were inspected.
   7. For each ORF, a global pairwise sequence alignment with the best *blastp* hit found in the ISfinder database was performed and visualized using JalView v2.10.5 [5].

## Findings

Here we report four examples of novel IS elements detected by *digIS*: pnov1, pnov2, pnov3, and pnov4. The first two were detected by both *digIS* and competitive tools; the last two were detected by *digIS* exclusively.

## LM997411.1 - *Methylacidiphilum fumariolicum* SolV

### **Report for putative novel IS element *pnov1***

Dotplot


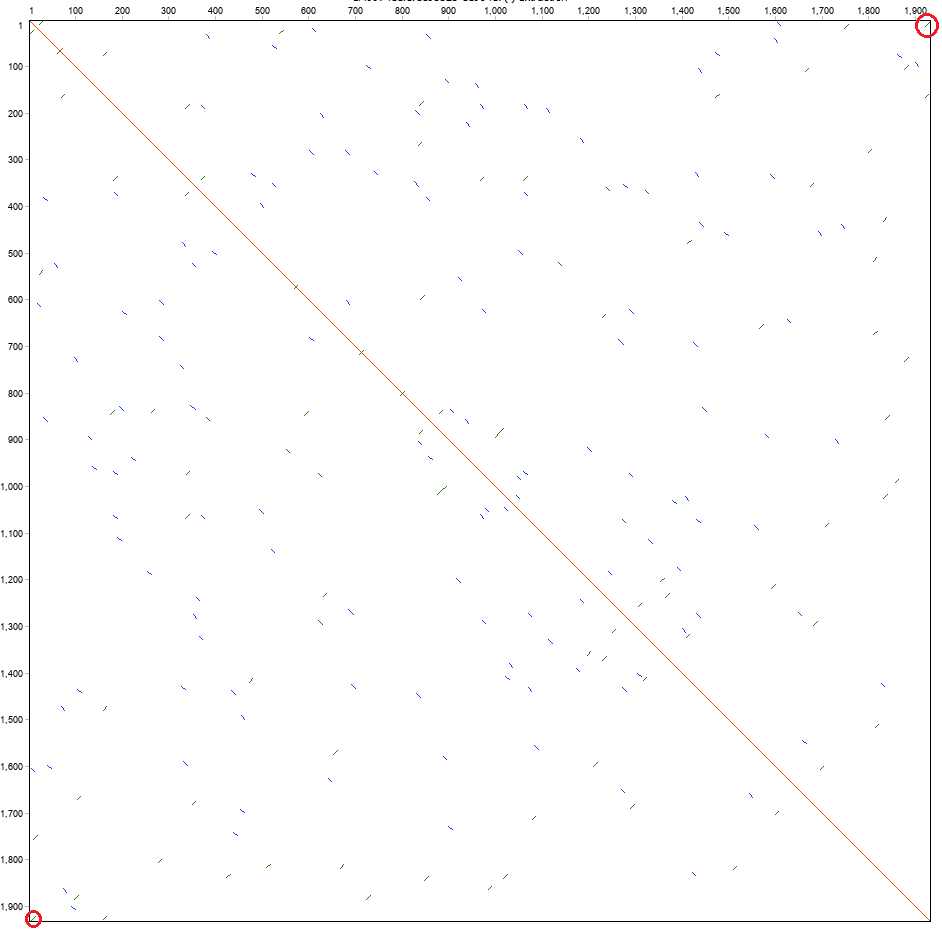


Figure 1. Dotplot of representative sequence of putative novel element *pnov1* identified in LM997411. The IRs are highlighted in red circles.

###

MSA


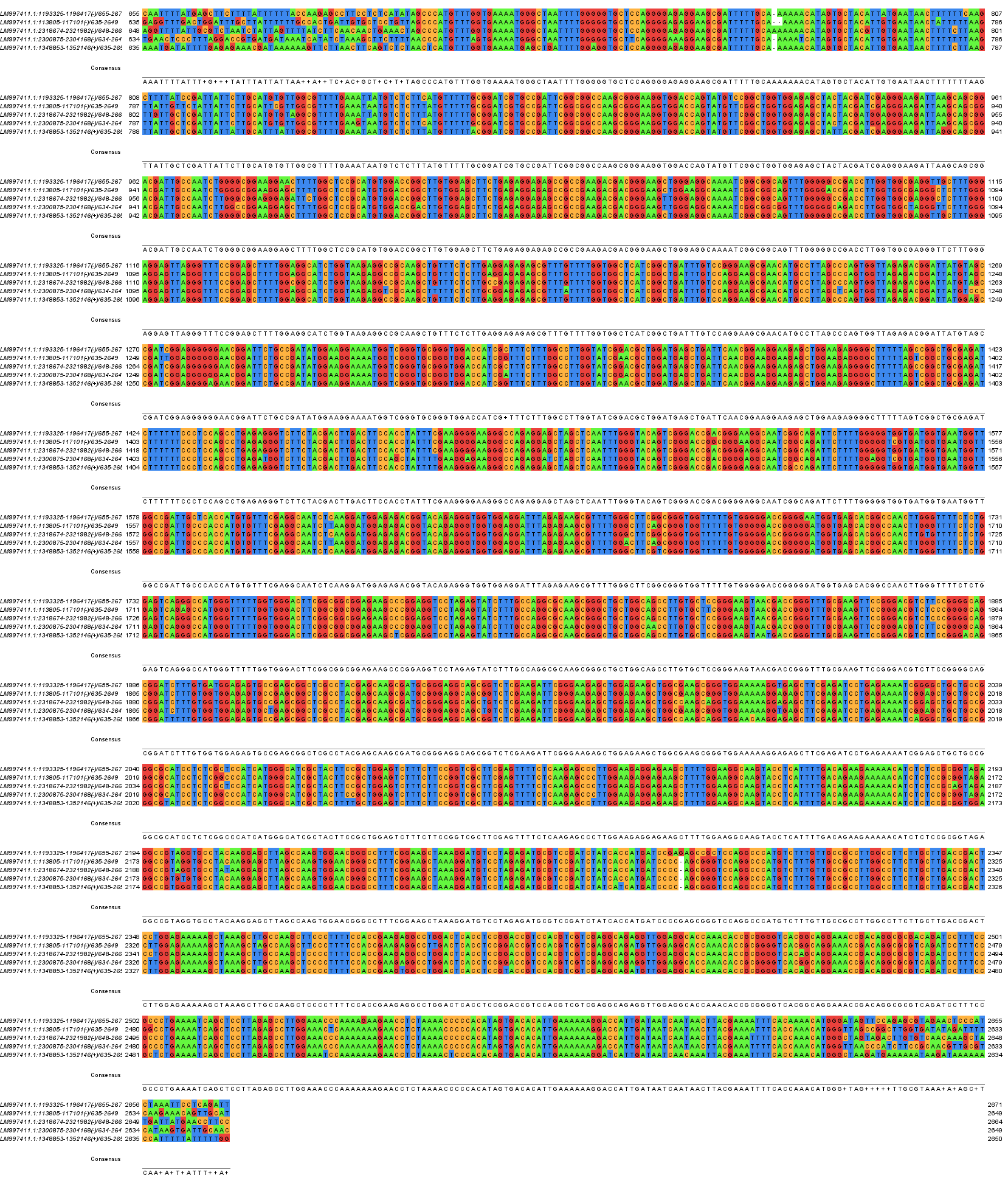


Figure 2. MSA of various copies of the *pnov1* element belonging to the same cluster.

Sequence DNA (1930 bp)

DRs are in italics, IRs are underlined.

*TA*GCCCATGTTTGGTGAAAATGGGCTAATTTTGGGGGTGCTCCAGGGGAGAGGAAGCGATTTTTGCAAAAAACATAGTGCTACATTATGAATAACTTTTTTCAAGCTTTTATCCGATTATTCTTGCATGTGTTGGCGTTTTGAAATTATGTCTCTTCATGTTTTTGCGGATCGTGCCGATTCGGCGGCCAAGCGGGAAGGTGGACCAGTATGTCCGGCTGGTGGAGAGCTACTACGATCGAGGGAAGATTAAGCAGCGGACGATTGCCAATCTGGGGCGGAAGGAACTTTTGGCTCCGCATGTGGACCGGCTTGTGGAGCTTCTGAGAGGAGAGCCGCCGAAGACGACGGGAAGCTGGGAGGCAAAATCGGCGGCAGTTTGGGGGCCGACCTTGGTGGCGAGGTTGCTTTGGGAGGAGTTAGGGTTTCCGGAGCTTTTGGAGGCATCTGGTAAGAGGCCGCAAGCTGTTTCTCTTGAGGAGAGAGCGTTTGTTTTGGTGGCTCATCGGCTGATTTGTCCGGGAAGCGAACATGCCTTAGCCCAGTGGTTAGAGACGGATTATGTAGCCGATCGGAGGGGGGAACGGATTCTGCCGATATGGAAGGAAAATGGTCGGGTGCGGGTGGACCATCGCTTTCTTTGGCCTTGGTATCGGACGCTGGATGAGCTGATTCAACGGAAGGAAGAGCTGGAAGAGGGGCTTTTTAGCCGGCTGCGAGATCTTTTTTCCCTCCAGCCTGAGAGGGTCTTCTACGACTTGACTTCCACCTATTTCGAAGGGGAAGGGCCAGAGGAGCTAGCTCAATTTGGGTACAGTCGGGACCGACGGGAAGGCAATCGGCAGATTCTTTTGGGGGTGGTGATGGTGAATGGTTGGCCGATTGCTCACCATGTGTTTCGAGGCAATCTCAAGGATGGAGAGACGGTACAGAGGGTGGTGGAGGATTTAGAGAAGCGTTTTGGGCTTCGGCGGGTGGTTTTTGTGGGGGACCGGGGAATGGTGAGCACGGCCAACTTGGGTTTTCTCTGGAGTCAGGGCCATGGGTTTTTGGTGGGACTTCGGCGGCGGAGAAGCCCGGAGGTCCTAGAGTATCTTTGCCAGGCGCAAGCGGGCTGCTGGCAGCCTTGTGCTCCGGGAAGTAACGACCGGGTTTGCGAAGTTCCGGGACGTCTTCCGGGGCAGCGGATCTTTGTGATGGAGAGTGCCGAGCGGCTCGCCTACGAGCAAGCGATGCGGGAGGCAGCGGTCTCGAAGATTCGGGAAGAGCTGGAGAAGCTGGCGAAGCGGGTGGAAAAAGGTGAGCTTCGAGATCCTGAGAAAATCGGGGCTGCTGCCGGGCGCATCCTCTCGCTCCATCATGGGCATCGCTACTTCCGCTGGAGTCTTTCTTCCGGTCGCTTCGAGTTTTCTCAAGAGCCCTTGGAAGAGGAGAAGCTTTTGGAAGGCAAGTACCTCATTTTGACAGAAGAAAAACATCTCTCCGCGGTAGAGGCCGTAGGTGCCTACAAGGAGCTTAGCCAAGTGGAACGGGCCTTTCGGAAGCTAAAGGATGTCCTAGAGATGCGTCCGATCTATCACCATGATCCGAGAGCCGCTCCAGGCCCATGTCTTTGTTGCCGCCTTGGCCTTCTTGCTTGACCGACTCCTGGAGAAAAAGCTAAAGCTTGCCAAGCTTCCCTTTTCCACCGAAGAGGCCTGGACTCACCTCCGGACCGTCCACGTCGTCGAGGCAGAGGTTGGAGGCACCAAACACCGCGGGGTCACGGCAGGAAACCGACAGGCGCGACAGATCCTTTCCGCCCTGAAAATCAGCTCCTTAGAGCCTTGGAAACCCAAAAGAAGAACCTCTAAAACCCCCACATAGTGACACATTGAAAAAAGGACCATTGATAATCAATAACTTACGAAAATTTCACAAAACATGGGA*TA*

IRs/DRs

IRL = CCCATGTTT(9 bp)

IRR = AAACATGGG(9 bp)

DR = TA (2 bp)

Sequence ORF1

frame +2, length = 507 aa, start = 116, end = 1639

MILACVGVLKLCLFMFLRIVPIRRPSGKVDQYVRLVESYYDRGKIKQRTIANLGRKELLAPHVDRLVELLRGEPPKTTGSWEAKSAAVWGPTLVARLLWEELGFPELLEASGKRPQAVSLEERAFVLVAHRLICPGSEHALAQWLETDYVADRRGERILPIWKENGRVRVDHRFLWPWYRTLDELIQRKEELEEGLFSRLRDLFSLQPERVFYDLTSTYFEGEGPEELAQFGYSRDRREGNRQILLGVVMVNGWPIAHHVFRGNLKDGETVQRVVEDLEKRFGLRRVVFVGDRGMVSTANLGFLWSQGHGFLVGLRRRRSPEVLEYLCQAQAGCWQPCAPGSNDRVCEVPGRLPGQRIFVMESAERLAYEQAMREAAVSKIREELEKLAKRVEKGELRDPEKIGAAAGRILSLHHGHRYFRWSLSSGRFEFSQEPLEEEKLLEGKYLILTEEKHLSAVEAVGAYKELSQVERAFRKLKDVLEMRPIYHHDPRAAPGPCLCCRLGLLA

Comparison with the ISfinder database at DNA level using blastn

The best hit against the ISfinder database at DNA level was not significant as it matches with only a short region (24 bp) of ISTel4. A similar IS element is not present in the ISfinder database.

### **
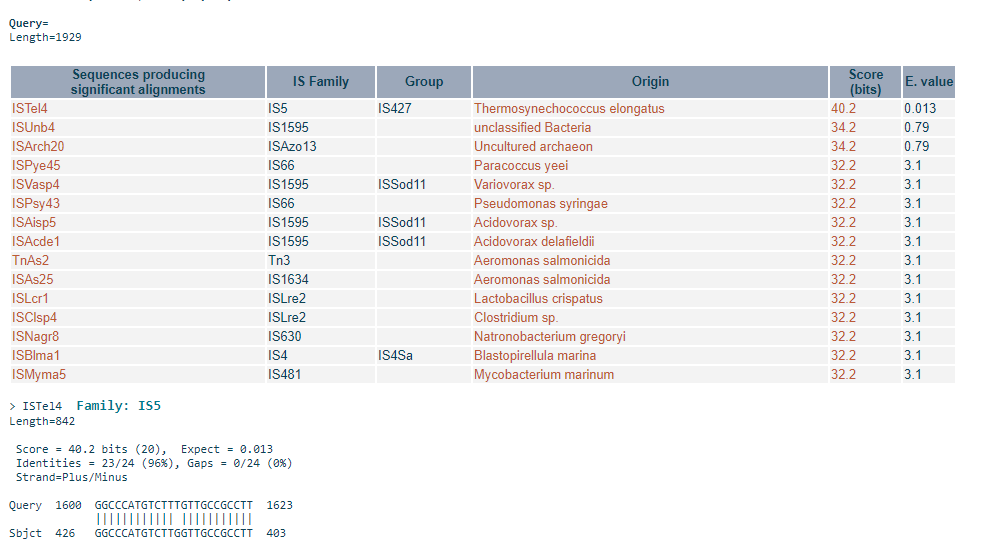
**

Comparison with the ISfinder database at ORF level using blastp

The best hit with the ISfinder database at ORF level was significant (e-value = 3e-63). However, the global identity was only 32.27%, which is considered low.


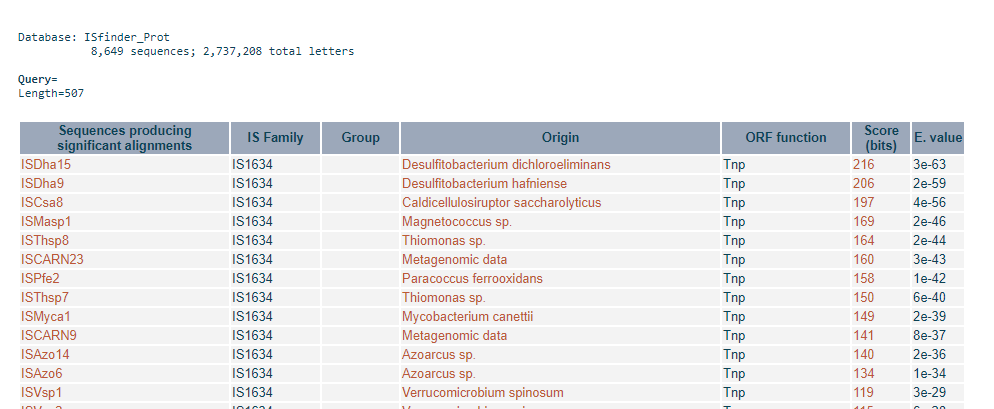


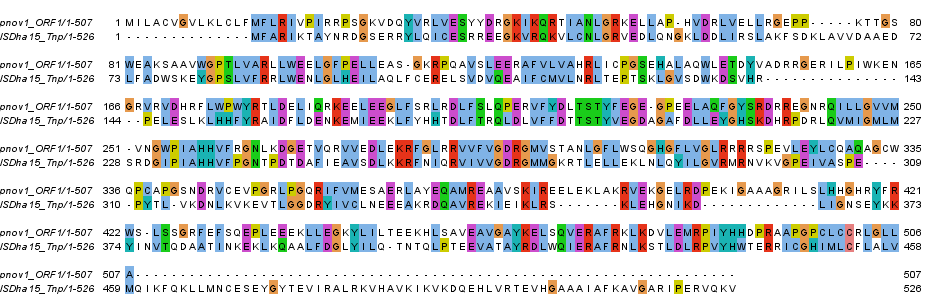


Figure 3. Global alignment of *pnov1* ORF1 and the best hit (ISDha15) detected by blastp in the ISfinder database. The identity at the global level is 32.27%.

Summary

A novel IS element *pnov1* was identified in *Methylacidiphilum fumariolicum* SolV. *pnov1* has a size of 1930 bp and carries a single ORF encoding a probable transposase of 503 amino acid residues homologous (32.27% identity) to that of ISDha15 found in *Desulfitobacterium hafniense* strain TCE1. *pnov1* includes inverted repeats of length 9 bp and is flanked by direct repeats of length 2 bp. Based on the homology with known Tnps and the structural features, *pnov1* belongs to the IS1634 family.

### **Report for putative novel IS element *pnov2***

Dotplot


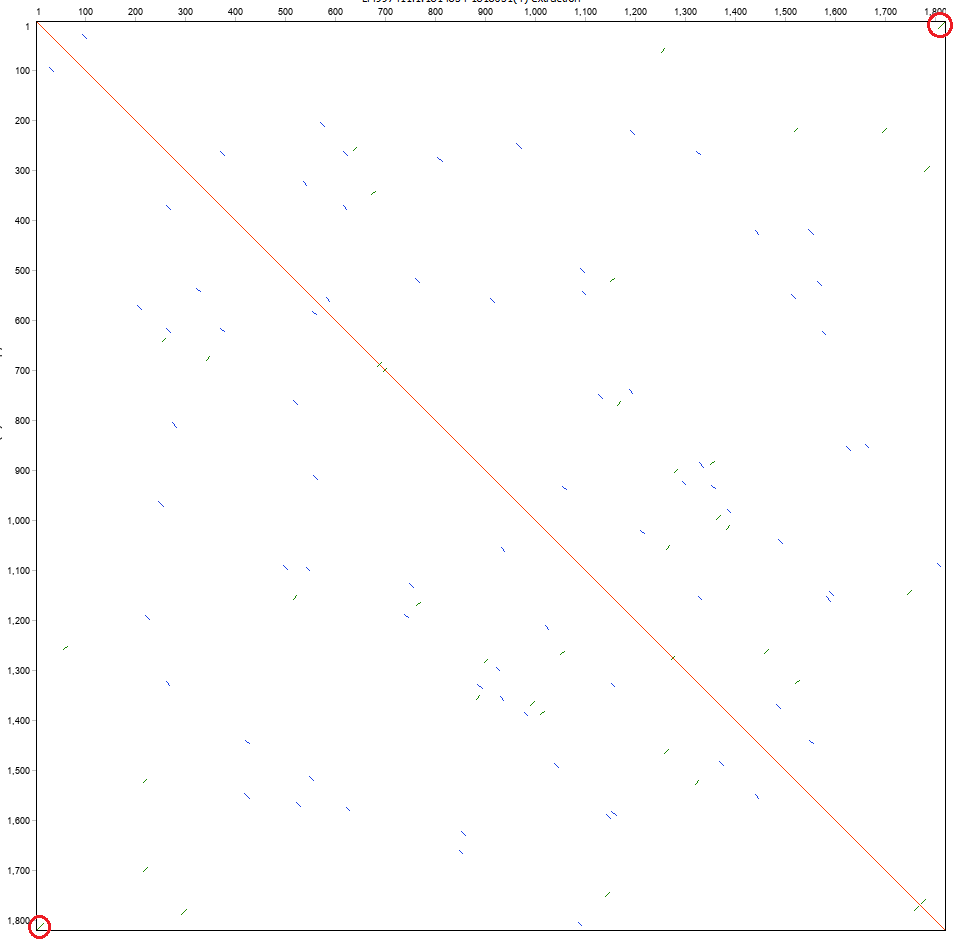


Figure 4. Dotplot of representative sequence of putative novel element *pnov2* identified in LM997411. The IRs are highlighted in red circles.

####

####

MSA


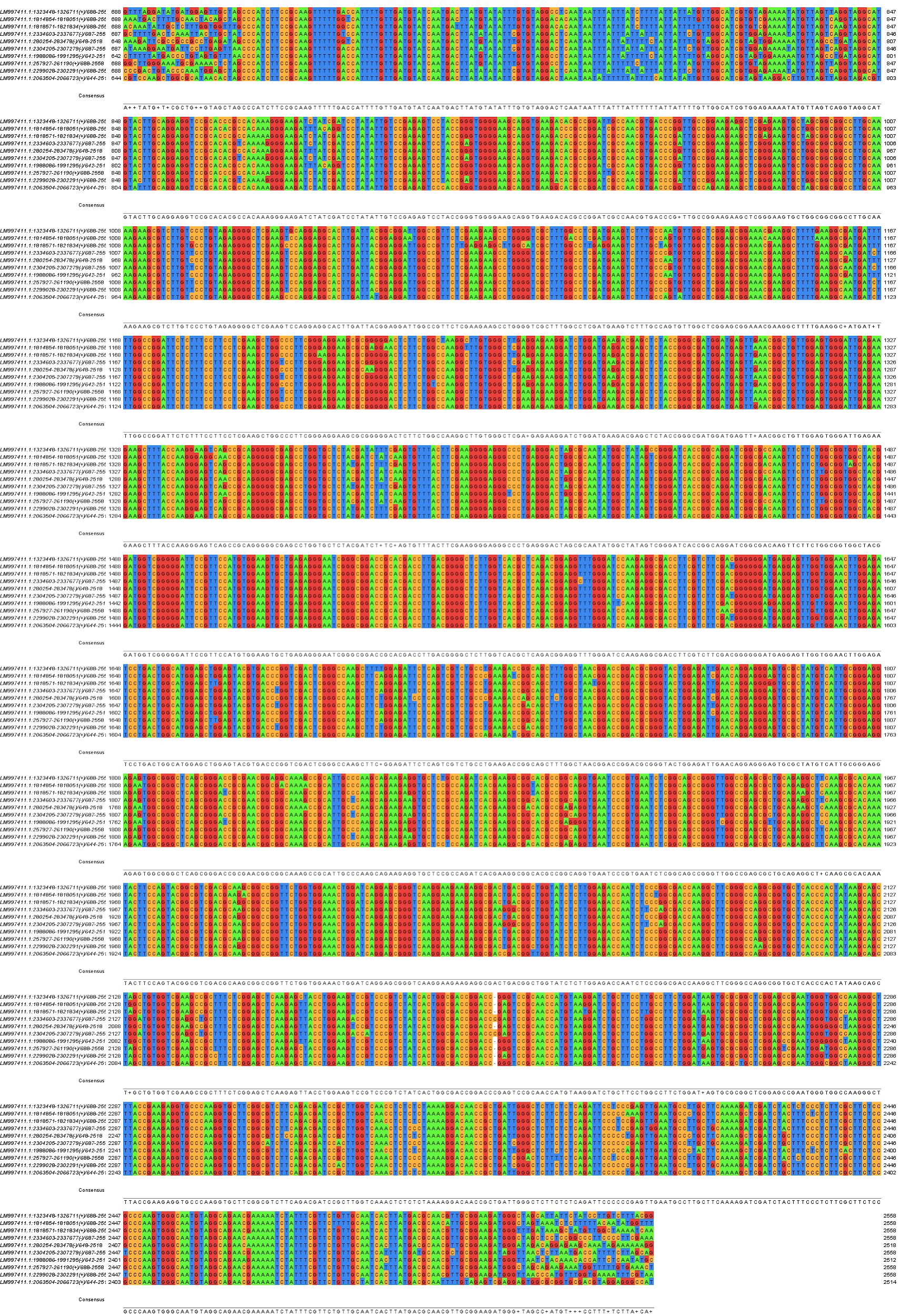


Figure 5. MSA of various copies of the *pnov2* element belonging to the same cluster.

Sequence DNA (1817 bp)

DRs are in italics, IRs are underlined.

CCCATCTTCCGCAAGTTTTTGGTCATTTTGTTGATGTATCAATGACTTATGTATATTTGTGTAGGACACAATAATTTATTTATCTTTTATTATTTTGTTGGCATCGTGTCGAAAATATGTTAGTCAGGTAGGCATGTACTTGCAGGAGGTCCGCACACGCCACAAAGGGAAGATTTACAGGTCCTATATTGTCCGAGAGTCCTACCGGGTGGGGAAGCAGGTGAAGACCCGCCGGATCGCCAACGTGACCCGGTTGCCGGAAGAAGCTCGGGAAGTGCTGGCGGCGGCCTTGCAAAAGAAGCGTCTTGTCCCTGTAGAGGGGCTCGAAGTCCAGGAGGCACTTGATTACGGAGGAATGGCCGTTCTCGAAGAAGCCTGGGGTCGCTTTGACCTCGATGAAGTCTTTGCCAGTGTTGGCTCGGAGCGGAAACAAAGGCTTTTAAAGGCGATGATTTTTGGCCGGATTCTCTTTCCTTCCTCGAAGCTGGCCCTTCGGGAGGAAGCGCGAGGAACTCTTCTGGCCAAGGTTTGTGGGCTCGAGGAGAAGGATCTGGATGAGGACGAGCTCTACCGGGCGATGGATGAGTTAAACGGCTGTTGGAGTGGGATTGAGAAGAAGCTTTACCAAGGGAGGCAACCGCAGGGGGCGAGCCTGGTGCTCTACGATCTATCAAGTGTTTACTTCGAAGGGGAGGGCCCTGAGGGACTGGCGCAATATGGCTATAGTCGGGATCACCGGCAGGATCGGCGACAAGTTCTTCTGGCGGTGGCTACGGATGGTCGGGGGATTCCGTTCCATGTGGAAGTGCTGAGAGGGAATCGGGCGGACCGCACGACCTTGACGGGGCTCTTGGTCACGCTCAGACGGAGGTTTGGGATCCAAGAGGCGACCTTCGTCTTCGATGGGGGGATGAGGAGTTGGTGGAACTTGGAGATCCTGACTGGCATGGAGCTGGAGTACGTGACCCGGTCGACTCGGGCCAAGCTTCAGGAGATTCTCAGTCGTCTGCCCGAAGATCGGCAGCTTTGGCTAACGGACCGGACGCGGGTACTGGAGATCGAACAGGAGGGAGTGCGCTATGTCATTGCGGGAGGAGAATGGCGGGCTCAGCGGGACCGCGAACGGCGACAAAACCGCATTGCCCAAGCAGAAGAGGTGCTCCGCCAGATCACGAAGGCGGTACGCCGGCAGGTGAATCCCGTGAATCTCGGCAGCCGGGTTGGCCGAGCGCTGCAGAGGCTCCAAGCGCACAAATACTTCCAGTACGGCGTCGACGAAGACGGCCGGTTCTGGTGGAAACTGGATCAGGAGCGGGTCAAGGAAGAAGAGGCGACTGACGGCTGGTATCTCTTGGAGACCAATCTCCTGGCGACCAAGGCTTCGGGCCAGGCGGTGCTCACCCACTATAAGCAGCTGGCTGTGGTCGAAGCCGCTTTCTCGGAGCTCAAGAGTTACCTGGAAGTCCGTCCCGTCTATCACTGGCGACCGGACCGAGTCCGCAACCATGTAAGGATCTGCTTCCTTGCCTTCTGGATAAGTGCGCGGCTCGGAGCCGAATGGGTGGCCAAGGGCTTTACCGAAGAGGTGCCCAAGGTGCTTCGGCGTCTTCAGACGATCCGCTTGGTCAACCTCTCTCTAAAAGGACAACCGCTGATTGGGCTCTTCTCTCAGATTCCCCCAAAGTTGAATGCCCTGCTTCAAAAGCTCGATCTACTTTCTCTCTTCGCTTCTCCGCCCAAGTGGGCAATGTAGGCAGAACGAAAAATCTATTTCGTTCTTTTGCAATCACTTATGACGCAACATTGCGGAAGATGGG

IRs/DRs

IRL = CCCATCTTCCGCAA (14 bp)

IRR = TTGCGGAAGATGGG (14 bp)

DR = N/A

Sequence ORF1

frame +2, length = 539 aa, start = 71, end = 1753

MYLQEVRTRHKGKIYRSYIVRESYRVGKQVKTRRIANVTRLPEEAREVLAAALQKKRLVPVEGLEVQEALDYGGLAVLEEAWGRFGLDEVFANVGSERKRRLLKAMIFGRILFPSSKLALREEARGTLLAKACGLEEKDLDEDELYRAMDELNGCWSGIEKKLYQGSQPQGASLVLYDISSVYFEGEGPEGLAQYGYSRDHRQDRRQVLLAVATDGRGIPFHVEVLRGNRADRTTLTGLLVTLRRRFGIQEATFVFDGGMRSWWNLEILTGMELEYVTRSTRAKLLEILSRLPEDRQLWLTDRTRVLEIEQEGVRYVIAGGEWRAQRDRERRQSRIAQAEEVLCQITKAARRQVNPVNLGSRVGRALQRLQAHKYFQYGVDASGRFWWKLDQERVKEEEATDGWYLLETNLPATKASGQAVLTHYKQLAVVEAAFSELKSYLEVRPVYHWRPDRVRNHVRICFLAFWISARLGAEWVAKGFTEEVPKVLRRLQTIRLVNLSLKGQPLIGLFSQIPPELNALLQKIDLLSLFASPPKWAM

Comparison with the ISfinder database at DNA level using blastn

The best hit against the ISfinder database at DNA level was not significant as it matches with only a short region (18 bp) of ISAzs30. A similar IS element is not present in the ISfinder database.


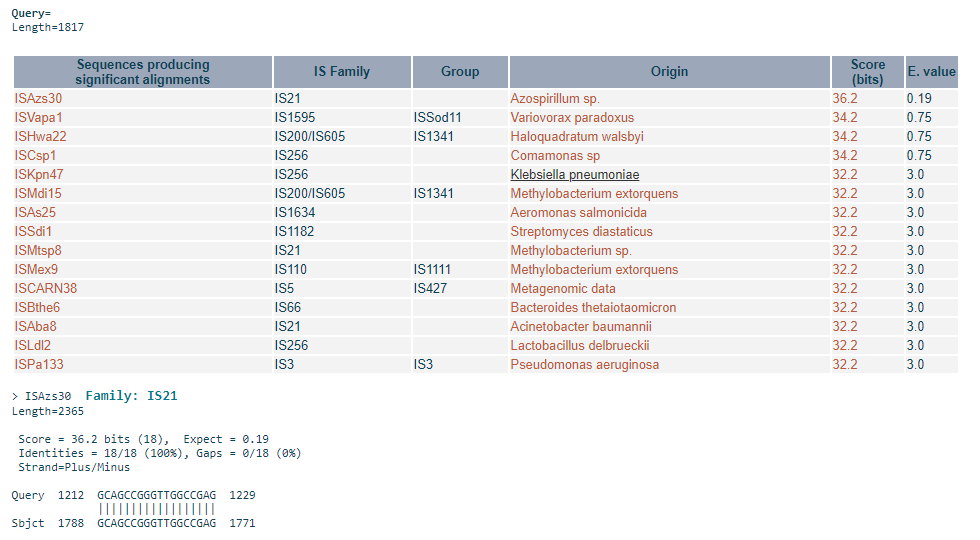


Comparison with ISfinder database at ORF level using blastp

The best hit with the ISfinder database at ORF level, ISMasp1 was significant (e-value = 1e-70). However, the global identity was only 30.35%, which is considered low.


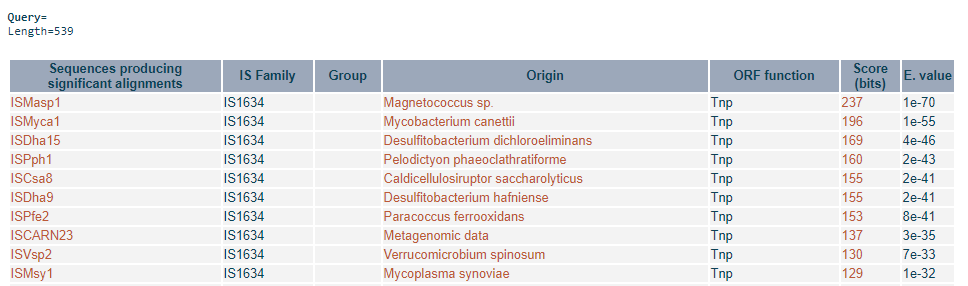


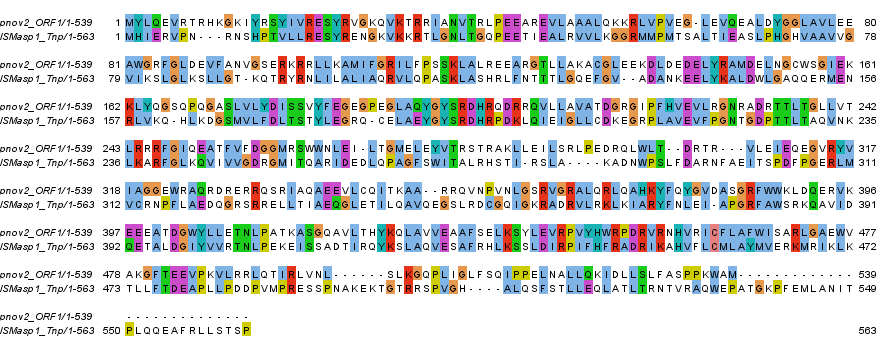


Figure 6. Global alignment of *pnov2* ORF1 and the best hit (ISMasp1 Tnp) detected by blastp in the ISfinder database. The identity at the global level is 30.35%.

Summary

A novel IS element *pnov2* was identified in *Methylacidiphilum fumariolicum* SolV. *pnov2* has a size of 1817 bp and contains a single ORF encoding a probable transposase of 539 amino acid residues homologous (30.35% identity) to that of ISMasp1 found in *Magnetococcus sp*. MC-1. *pnov2* includes inverted repeats of length 14 bp. DRs were not identified. Based on the homology with known Tnps and structural features, *pnov2* belongs to the IS1634 family.

###

### **Report for putative novel IS element *pnov3***

Dotplot


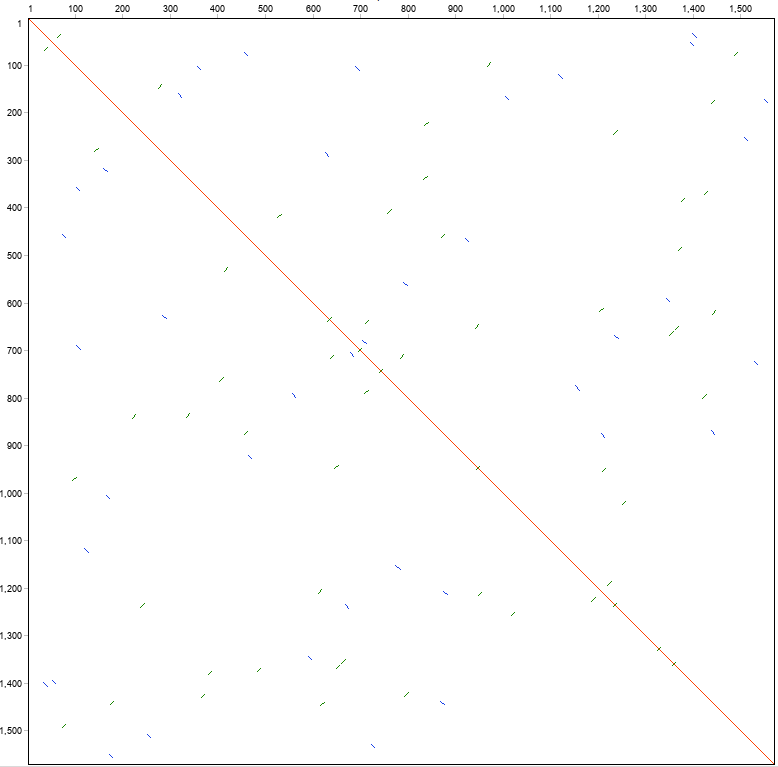


Figure 7. Dotplot of representative sequence of putative novel element *pnov3* identified in LM997411. DRs or IRs were not detected.

####

MSA


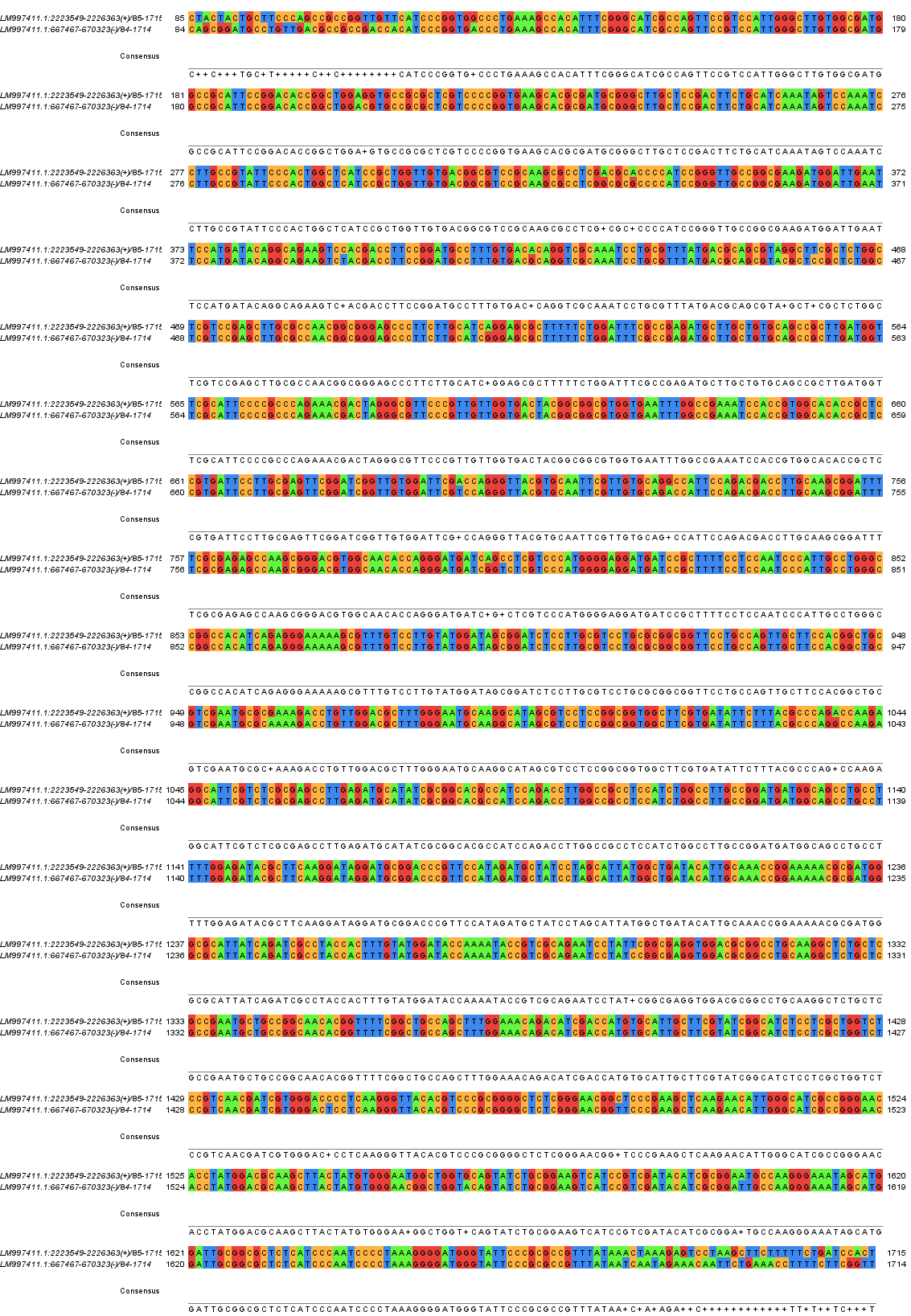


Figure 8. MSA of two copies of the *pnov3* element belonging to the same cluster.

Sequence DNA (1567 bp)

No DRs or IRs were detected.

CATCCCGGTGGCCCTGAAAGCCACATTTCGGGCATCGCCAGTTCCGTCCATTGGGCTTGTGGCGATGGCCGCATTCCGGACACCGGCTGGAGGTGCCGCGCTCGTCCCCGGTGAAGCACGCGATGCGGGCTTGCTCCGACTTCTGCATCAAATAGTCCAAATCCTTGCCGTATTCCCACTGGCTCATCCGCTGGTTGTGACGGCGTCCGCAAGCGCCTCGACGCACCCCATCCGGGTTGCCGGCGAAGATGGATTGAATTCCATGATACAGGCAGAAGTCCACGACCTTCCGGATGCCTTTGTGACACAGGTCGCAAATCCTGCGTTTATGACGCAGCGTAGGCTTCGCTCTGGCTCGTCCGAGCTTGCGCCAACGGCGGGAGCCCTTCTTGCATCAGGAGCGCTTTTTCTGGATTTCGCCGAGATGCTTGCTGTGCAGCCGCTTGATGGTTCGCATTCCCCGCCCAGAAACGACTAGGGCGTTCCCGTTGTTGGTGACTACGGCGGCGTGGTGAATTTGGCCGAAATCCACCGTGGCACACCGCTCCGTGATTCCTTGCGAGTTCGGATCGGTTGTGGATTCGACCAGGGTTACGTGCAATTCGTTGTGCAGGCCATTCCAGACGACCTTGCAAGCGGATTTTCGCGAGAGCCAAGCGGGACGTGGCAACACCAGGGATGATCAGCCTCGTCCCATGGGGAGGATGATCCGCTTTTCCTCCAATCCCATTGCCTGGGCCGGCCACATCAGAGGGAAAAAGCGTTTGTCCTTGTATGGATAGCGGATCTCCTTGCGTCCTGCGCGGCGGTTCCTGCCAGTTGCTTCCACGGCTGCGTCGAATGCGCGAAAGACCTGTTGGACGCTTTGGGAATGCAAGGCATAGCGTCCTCCGGCGGTGGCTTCGTGATATTCTTTACGCCCAGACCAAGAGGCATTCGTCTCGCGAGCCTTGAGATGCATATCGCGGCACGCCATCCAGACCTTGGCCGCCTCCATCTGGCCTTGCCGGATGATGGCAGCCTGCCTTTTGGAGATACGCTTCAAGGATAGGATGCGGACCCGTTCCATAGATGCTATCCTAGCATTATGGCTGATACATTGCAAACCGGAAAAACGCGATGGGCGCATTATCAGATCGCCTACCACTTTGTATGGATACCAAAATACCGTCGCAGAATCCTATTCGGCGAGGTGGACGCGGCCTGCAAGGCTCTGCTCGCCGAATGCTGCCGGCAACACGGTTTTCGGCTGCCAGCTTTGGAAACAGACATCGACCATGTGCATTGCTTCGTATCGGCATCTCCTCGCTGGTCTCCGTCAACGATCGTGGGACCCCTCAAGGGTTACACGTCCCGCGGGGCTCTCGGGAACGGCTCCCGAAGCTCAAGAACATTGGGCATCGCCGGGAACACCTATGGACGCAAGCTTACTATGTGGGAATGGCTGGTGCAGTATCTGCGGAAGTCATCCGTCGATACATCGCGGAATGCCAAGGGAAATAGCATGGATTGCGGCGCTCTCATCCCAATCCCCTAAAGGGGATGGGTATTCCCGCGCCGTTTATAA

IRs/DRs

IRL = N/A

IRR = N/A

DR = N/A

Sequence ORF1

length = 158 aa, start = 2, end = 478, strand = +

MPVALKATFRASPVPSIGLVAMAAFRTPAGGAALVPGEARDAGLLRLLHQIVQILAVFPLAHPLVVTASASASTHPIRVAGEDGLNSMIQAEVHDLPDAFVTQVANPAFMTQRRLRSGSSELAPTAGALLASGALFLDFAEMLAVQPLDGSHSPPRND

Sequence ORF2

length = 105 aa, start = 734, end = 1051, strand = +

MGRPHQREKAFVLVWIADLLASCAAVPASCFHGCVECAKDLLDALGMQGIASSGGGFVIFFTPRPRGIRLASLEMHIAARHPDLGRLHLALPDDGSLPFGDTLQG

Sequence ORF3

length = 150 aa, start = 1085, end = 1537, strand = +

MMADTLQTGKTRWAHYQIAYHFVWIPKYRRRILFGEVDAACKALLAECCRQHGFRLPALETDIDHVHCFVSASPRWSPSTIVGPLKGYTSRGALGNGSRSSRTLGIAGNTYGRKLTMWEWLVQYLRKSSVDTSRNAKGNSMDCGALIPIP

Sequence ORF4

length = 134 aa, start = 1099, end = 1503, strand = +

MANRKNAMGALSDRLPLCMDTKIPSQNPIRRGGRGLQGSARRMLPATRFSAASFGNRHRPCALLRIGISSLVSVNDRGTPQGLHVPRGSRERLPKLKNIGHRREHLWTQAYYVGMAGAVSAEVIRRYIAECQGK

Sequence ORF5

length = 194 aa, start = 327, end = 911, strand = -

MSRSHRRRTLCLAFPKRPTGLSRIRRSRGSNWQEPPRRTQGDPLSIQGQTLFPSDVAGPGNGIGGKADHPPHGTRLIIPGVATSRLALAKIRLQGRLEWPAQRIARNPGRIHNRSELARNHGAVCHGGFRPNSPRRRSHQQRERPSRFWAGNANHQAAAQQASRRNPEKALLMQEGLPPLAQARTSQSEAYAAS

Sequence ORF6

length = 154 aa, start = 683, end = 1147, strand = -

MVGDLIMRPSRFSGLQCISHNARIASMERVRILSLKRISKRQAAIIRQGQMEAAKVWMACRDMHLKARETNASWSGRKEYHEATAGGRYALHSQSVQQVFRAFDAAVEATGRNRRAGRKEIRYPYKDKRFFPLMWPAQAMGLEEKRIILPMGRG

Comparison with ISfinder database at DNA level using blastn

The best hit against the ISfinder database at DNA level was not significant as it matches only with a short region (34 bp) of the ISTsi3 element belonging to the IS200/IS605 family, group IS605. A similar IS element is not present in the ISfinder database.


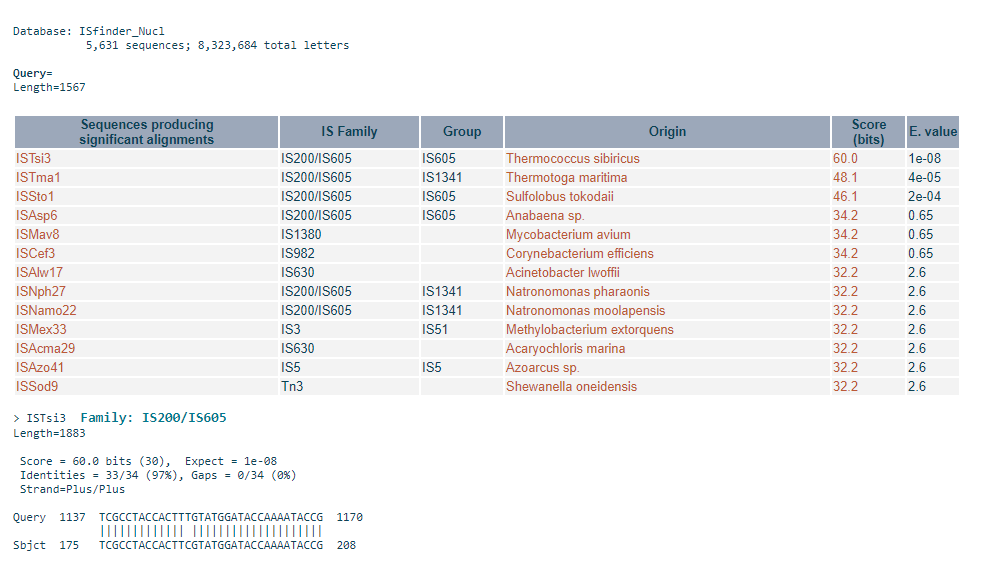


Comparison with ISfinder database at ORF level using blastp

ORF1


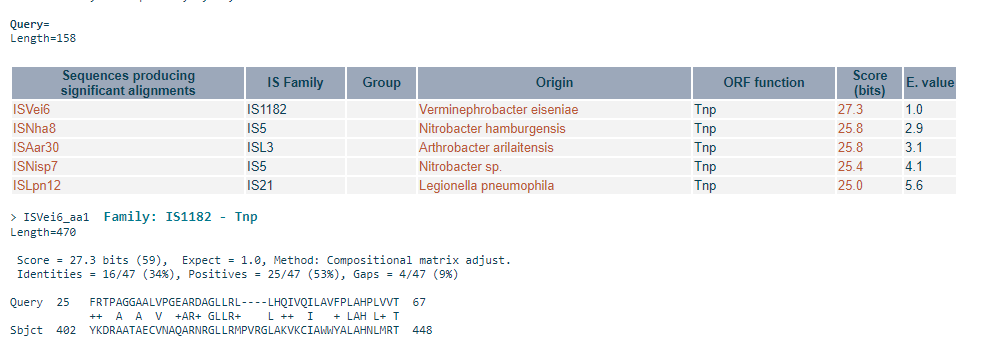


ORF2


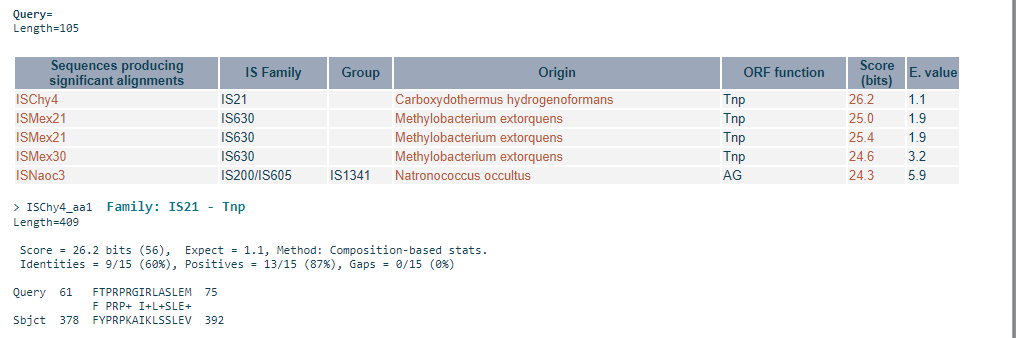


ORF3


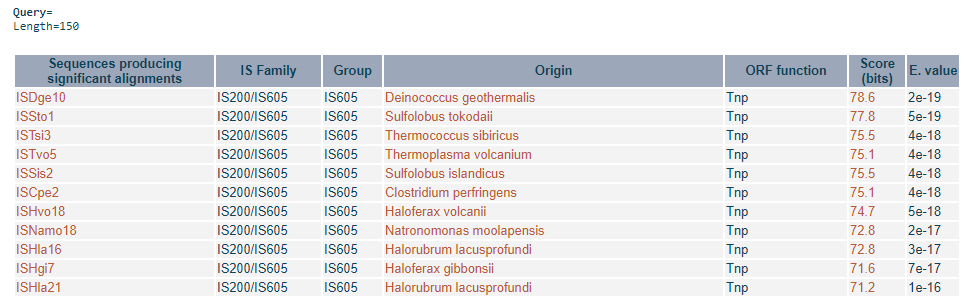


#####
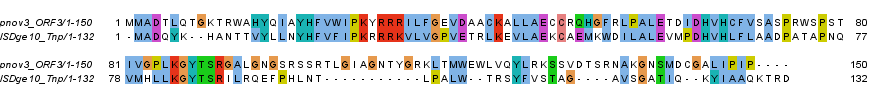


Figure 9. Global alignment of *pnov3* ORF3 and the best hit (ISDge10 Tnp) detected by blastp in the ISfinder database. The identity at the global level is 35.07%.

ORF4


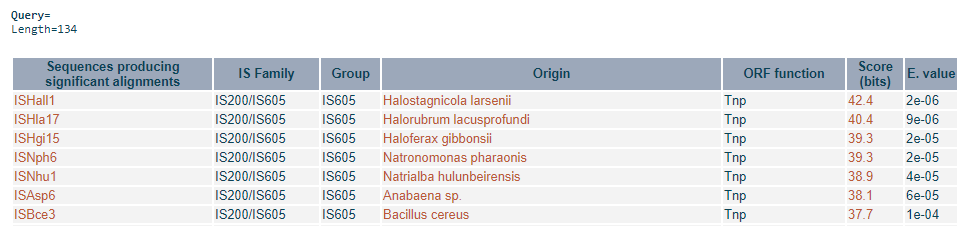


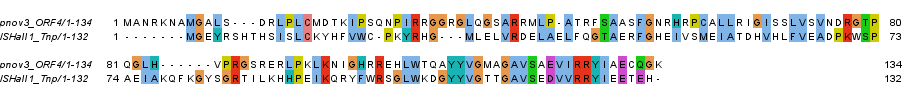


Figure 10. Global alignment of *pnov3* ORF4 and the best hit (ISHall1 Tnp) detected by blastp in the ISfinder database. The identity at the global level is 25.93%.

ORF5


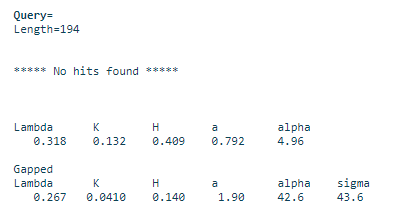


ORF6


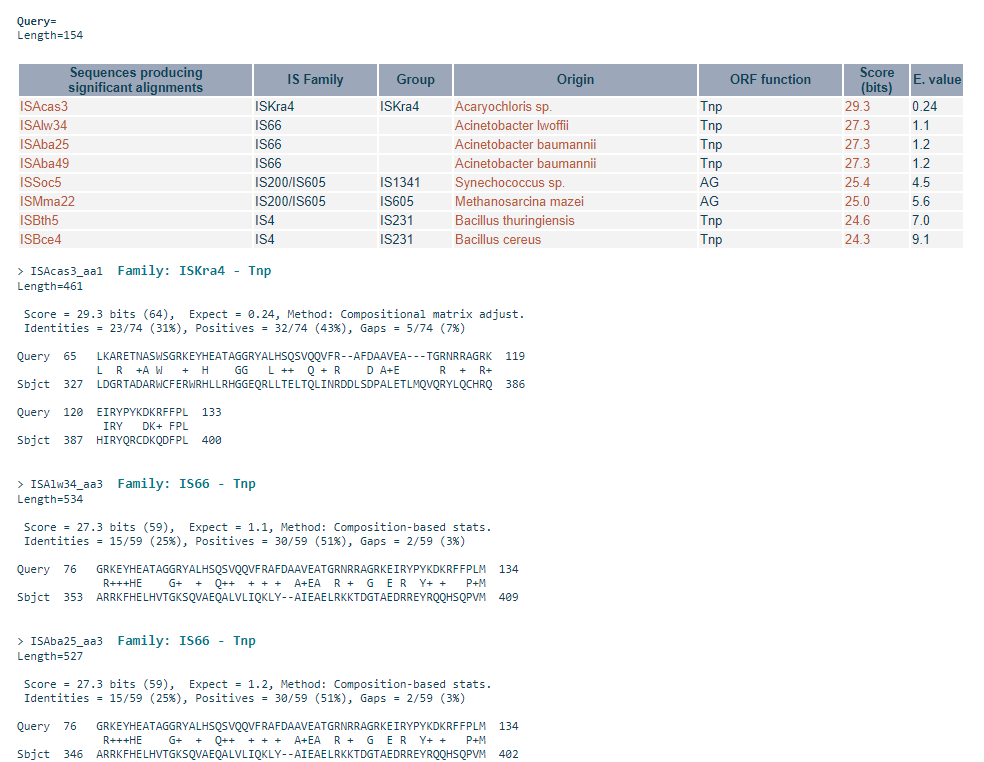


Summary

A novel IS element *pnov3* was identified in *Methylacidiphilum fumariolicum* SolV. *pnov3* has a size of 1567 bp. *pnov3 was* shown to carry multiple ORFs. Regions of ORF3 (150 aa) and ORF4 (134 aa) are homologous to transposases of ISDge10 (local identity 44%, alignment length = 90 aa) and ISHall1 (local identity 49%, alignment length = 39 aa), respectively. These transposases are classified as members of the IS200/IS605 family, group IS605. ORF1, ORF2, and ORF6 did not show significant similarity with records in the ISfinder database; for ORF5, no hits were found in the ISfinder database. However, these ORFs may represent accessory genes. No IRs or DRs were detected.

Generally, members of the IS200/IS605 family, group IS605, carry two ORFs: *tnpA* encoding Tpase, and *tnpB* (protein of unknown function) which is not required for transposition [9], in a given order. However, it was shown that ORFs encoding Tpase in the *pnov3* are located in the second part of the element, and ORFs encoding unknown proteins are located in the front - a reversal of the usual organization of members of IS200/IS605 family. *pnov3* may represent a member of a novel subfamily of the family IS200/IS605. However, further research is required. This element was identified exclusively by *digIS*.

##

## CP017599.1 - *Moorea producens* PAL-8-15-08-1

### **Report for putative novel IS element *pnov4***

Dotplot


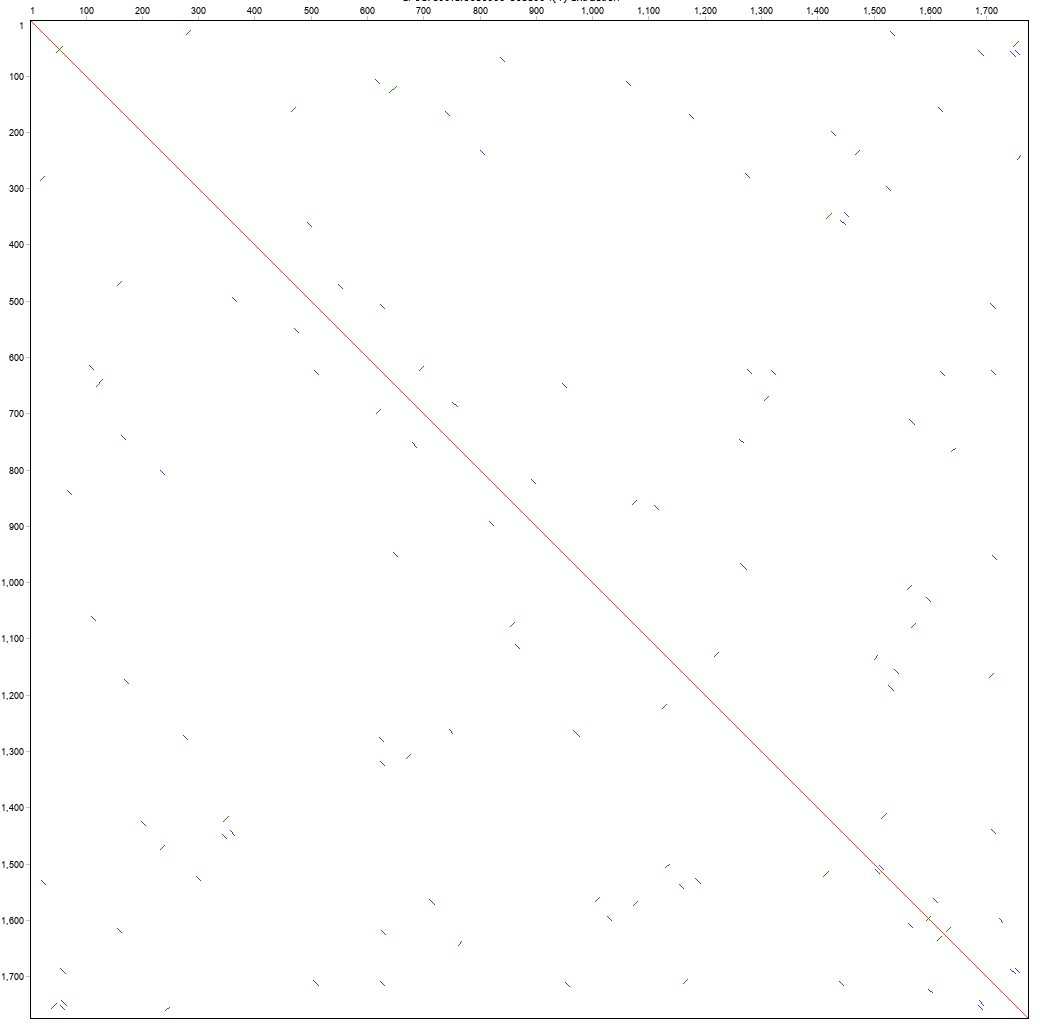


Figure 11. Dotplot of representative sequence of putative novel element *pnov4* identified in CP017599. IRs were not detected.

MSA


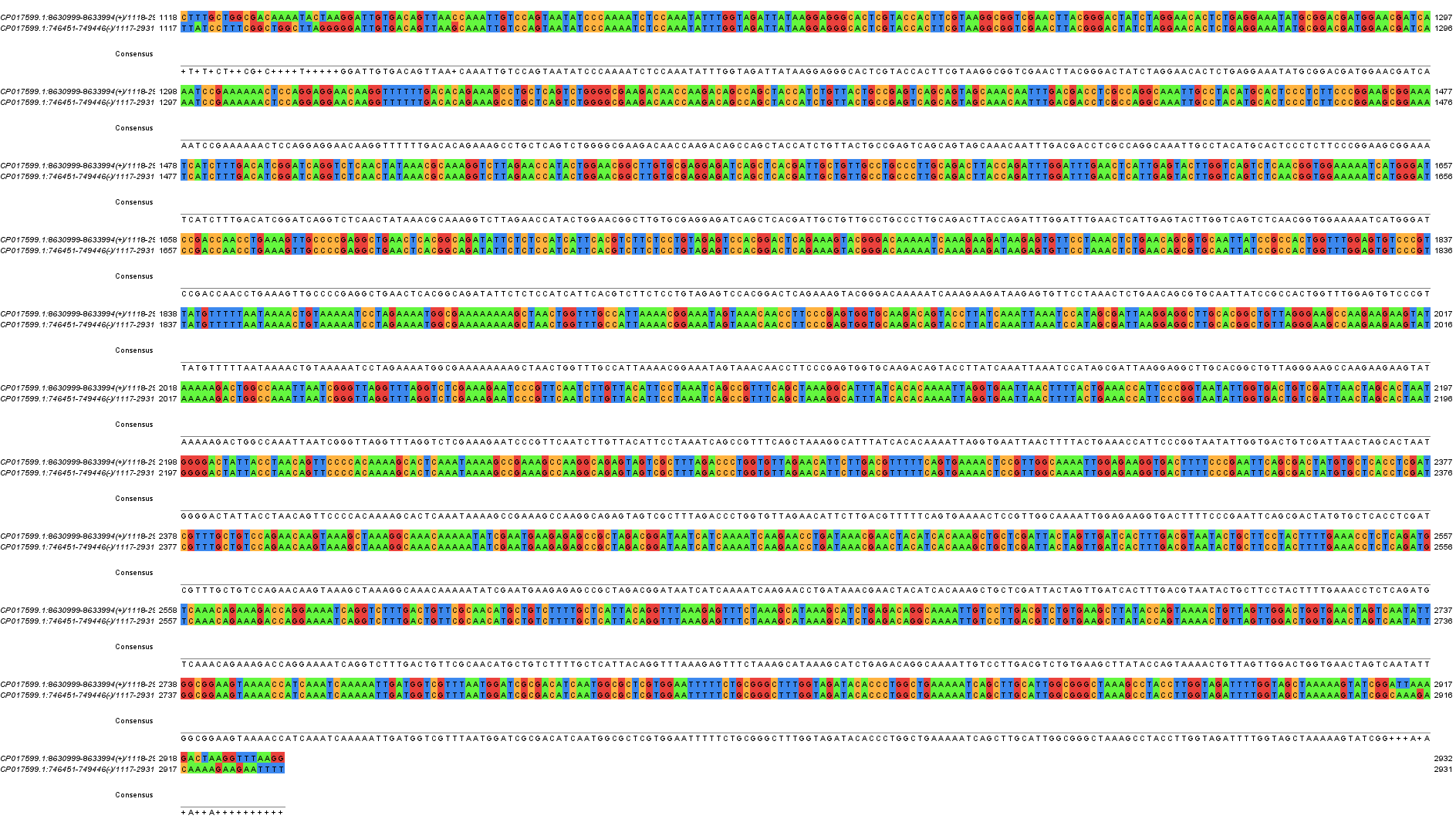


Figure 12. MSA of two copies of the *pnov4* element belonging to the same cluster.

Sequence DNA (1771 bp)

DRs are in italics.

*GG*ATTGTGACAGTTAACCAAATTGTCCAGTAATATCCCAAAATCTCCAAATATTTGGTAGATTATAAGGAGGGCACTCGTACCACTTCGTAAGGCGGTCGAACTTACGGGACTATCTAGGAACACTCTGAGGAAATATGCGGACGATGGAACGATCAAATCCGAAAAAACTCCAGGAGGAACAAGGTTTTTTGACACAGAAAGCCTGCTCAGTCTGGGGCGAAGACAACCAAGACAGCCAGCTACCATCTGTTACTGCCGAGTCAGCAGTAGCAAACAATTTGACGACCTCGCCAGGCAAATTGCCTACATGCACTCCCTCTTCCCGGAAGCGGAAATCATCTTTGACATCGGATCAGGTCTCAACTATAAACGCAAAGGTCTTAGAACCATACTGGAACGGCTTGTGCGAGGAGATCAGCTCACGATTGCTGTTGCCTGCCCTTGCAGACTTACCAGATTTGGATTTGAACTCATTGAGTACTTGGTCAGTCTCAACGGTGGAAAAATCATGGGATCCGACCAACCTGAAAGTTGCCCCGAGGCTGAACTCACGGCAGATATTCTCTCCATCATTCACGTCTTCTCCTGTAGAGTCCACGGACTCAGAAAGTACGGGACAAAAATCAAAGAAGATAAGAGTGTTCCTAAACTCTGAACAGCGTGCAATTATCCGCCACTGGTTTGGAGTGTCCCGTTATGTTTTTAATAAAACTGTAAAAATCCTAGAAAATGGCGAAAAAAAAGCTAACTGGTTTGCCATTAAAACGGAAATAGTAAACAACCTTCCCGAGTGGTGCAAGACAGTACCTTATCAAATTAAATCCATAGCGATTAAGGAGGCTTGCACGGCTGTTAGGGAAGCCAAGAAGAAGTATAAAAAGACTGGCCAAATTAATCGGGTTAGGTTTAGGTCTCGAAAGAATCCCGTTCAATCTTGTTACATTCCTAAATCAGCCGTTTCAGCTAAAGGCATTTATCACACAAAATTAGGTGAATTAACTTTTACTGAAACCATTCCCGGTAATATTGGTGACTGTCGATTAACTAGCACTAATGGGGACTATTACCTAACAGTTCCCCACAAAAGCACTCAAATAAAAGCCGAAAGCCAAGGCAGAGTAGTCGCTTTAGACCCTGGTGTTAGAACATTCTTGACGTTTTTCAGTGAAAACTCCGTTGGCAAAATTGGAGAAGGTGACTTTTCCCGAATTCAGCGACTATGTGCTCACCTCGATCGTTTGCTGTCCAGAACAAGTAAAGCTAAAGGCAAACAAAAATATCGAATGAAGAGAGCCGCTAGACGGATAATCATCAAAATCAAGAACCTGATAAACGAACTACATCACAAAGCTGCTCGATTACTAGTTGATCACTTTGACGTAATACTGCTTCCTACTTTTGAAACCTCTCAGATGTCAAACAGAAAGACCAGGAAAATCAGGTCTTTGACTGTTCGCAACATGCTGTCTTTTGCTCATTACAGGTTTAAAGAGTTTCTAAAGCATAAAGCATCTGAGACAGGCAAAATTGTCCTTGACGTCTGTGAAGCTTATACCAGTAAAACTGTTAGTTGGACTGGTGAACTAGTCAATATTGGCGGAAGTAAAACCATCAAATCAAAAATTGATGGTCGTTTAATGGATCGCGACATCAATGGCGCTCGTGGAATTTTTCTGCGGGCTTTGGTAGATACACCCTGGCTGAAAAATCAGCTTGCATTGGCGGGCTAAAGCCTACCTTGGTAGATTTTGGTAGCTAAAAAGTATC*GG*

IRs/DRs

IRL = N/A

IRR = N/A

DR = GG (2 bp)

Sequence ORF1

frame +1, length = 198 aa, start = 61, end = 657

MIRRALVPLRKAVELTGLSRNTLRKYADDGTIKSEKTPGGTRFFDTESLLSLGRRQPRQPATICYCRVSSSKQFDDLARQIAYMHSLFPEAEIIFDIGSGLNYKRKGLRTILERLVRGDQLTIAVACPCRLTRFGFELIEYLVSLNGGKIMGSDQPESCPEAELTADILSIIHVFSCRVHGLRKYGTKIKEDKSVPKL

Sequence ORF2

frame +2, length = 531 aa, start = 137, end = 1732

MRTMERSNPKKLQEEQGFLTQKACSVWGEDNQDSQLPSVTAESAVANNLTTSPGKLPTCTPSSRKRKSSLTSDQVSTINAKVLEPYWNGLCEEISSRLLLPALADLPDLDLNSLSTWSVSTVEKSWDPTNLKVAPRLNSRQIFSPSFTSSPVESTDSESTGQKSKKIRVFLNSEQRAIIRHWFGVSRYVFNKTVKILENGEKKANWFAIKTEIVNNLPEWCKTVPYQIKSIAIKEACTAVREAKKKYKKTGQINRVRFRSRKNPVQSCYIPKSAVSAKGIYHTKLGELTFTETIPGNIGDCRLTSTNGDYYLTVPHKSTQIKAESQGRVVALDPGVRTFLTFFSENSVGKIGEGDFSRIQRLCAHLDRLLSRTSKAKGKQKYRMKRAARRIIIKIKNLINELHHKAARLLVDHFDVILLPTFETSQMSNRKTRKIRSLTVRNMLSFAHYRFKEFLKHKASETGKIVLDVCEAYTSKTVSWTGELVNIGGSKTIKSKIDGRLMDRDINGARGIFLRALVDTPWLKNQLALAG

Comparison with the ISfinder database at DNA level using blastn

The best hit against the ISfinder database at DNA level was not significant as it matches with only a short region (24 bp) of ISC1913. A similar IS element is not present in the ISfinder database.


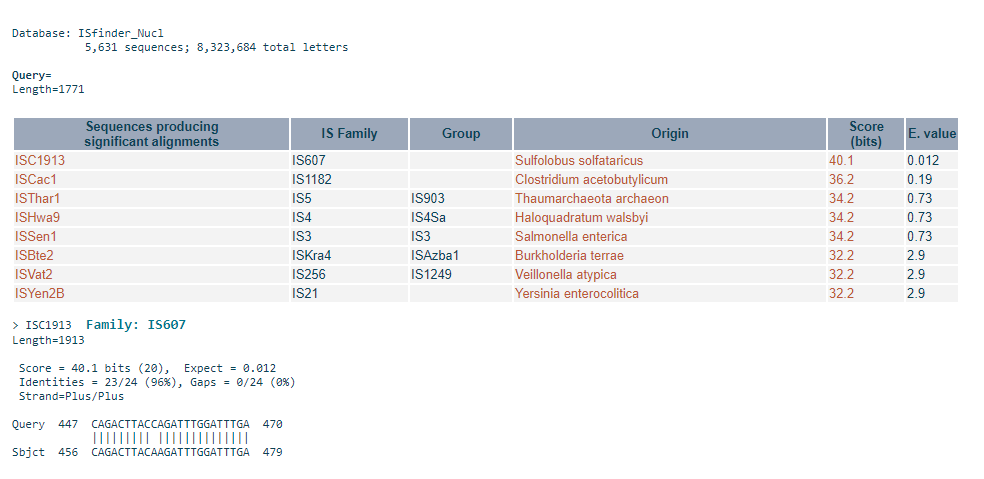


Comparison with the ISfinder database at ORF level using blastp

ORF1


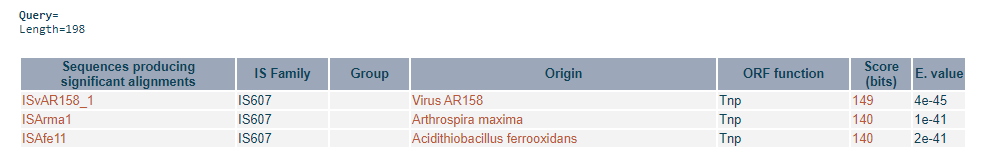


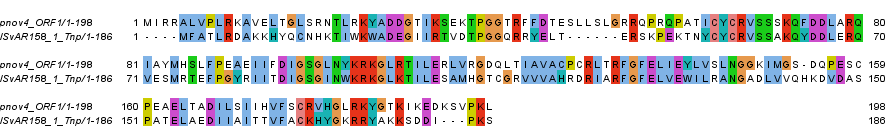


Figure 13. Global alignment of *pnov4* ORF1 and the best hit (ISvAR158_1 Tnp) detected by blastp in the ISfinder database. The identity at the global level is 40.51%.

ORF2


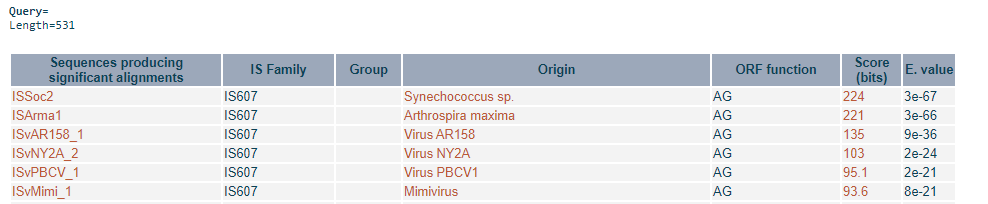


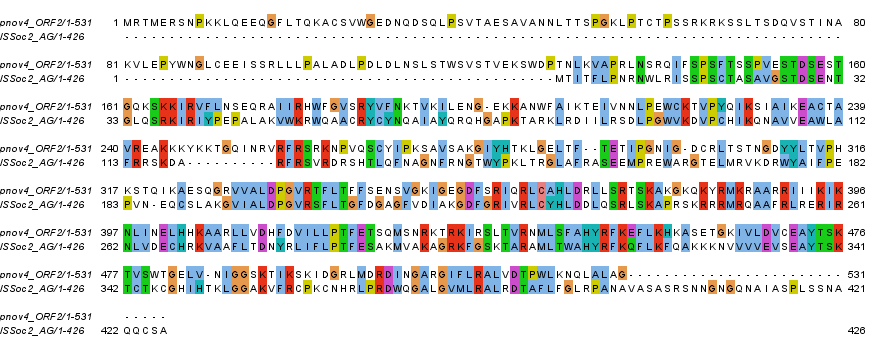


Figure 14. Global alignment of *pnov4* ORF2 and the best hit (ISSoc2 Accessory gene) detected by blastp in the ISFinder database. The identity at global level is 36.47%.

Summary

A novel IS element *pnov4* was identified in *Moorea producens PAL-8-15-08-1*. *pnov4* has a size of 1771 bp and it carries two overlapping ORFs. The first ORF encodes a probable transposase of 198 amino acid residues homologous (40.51% identity) to that of ISvAR158_1 found in Virus AR158 (host : *Chlorella* sp. NC64A) belonging to the IS607 family. The second ORF of 531 amino acid residues encodes a probable accessory gene which is homologous (36.47% identity) to an accessory gene found in *Synechococcus* sp and classified in the IS607 family. No IRs were and only short DRs of length 2 bp were detected.

Based on the sequence homology with known Tnps and structural features, *pnov4* belongs to the IS607 family. Moreover, *pnov4* was detected exclusively by *digIS*.

## Literature

1. Quinlan, A. R., & Hall, I. M. (2010). BEDTools: A flexible suite of utilities for comparing genomic features. Bioinformatics, 26(6), 841–842. <https://doi.org/10.1093/bioinformatics/btq033>
2. Li, W., & Godzik, A. (2006). Cd-hit: a fast program for clustering and comparing large sets of protein or nucleotide sequences. Bioinformatics, 22(13), 1658–1659. https://doi.org/10.1093/bioinformatics/btl158
3. Fu, L., Niu, B., Zhu, Z., Wu, S., & Li, W. (2012). CD-HIT: accelerated for clustering the next generation sequencing data. Bioinformatics (Oxford, England), 28, 3150–3152. <https://doi.org/10.1093/bioinformatics/bts565>
4. Sievers, F., Wilm, A., Dineen, D., Gibson, T. J., Karplus, K., Li, W., … Higgins, D. G. (2011). Fast, scalable generation of high-quality protein multiple sequence alignments using Clustal Omega. Molecular Systems Biology, 7(539). <https://doi.org/10.1038/msb.2011.75>
5. Waterhouse, A. M., Procter, J. B., Martin, D. M. A., Clamp, M., & Barton, G. J. (2009). Jalview Version 2 — a multiple sequence alignment editor and analysis workbench. 25(9), 1189–1191. <https://doi.org/10.1093/bioinformatics/btp033>
6. Geneious Prime 2020.2.4. ([https://www.geneious.com](http://www.geneious.com/))
7. Altschul, S. F., Gish, W., Miller, W., Myers, E. W., & Lipman, D. J. (1990). Basic local alignment search tool. Journal of Molecular Biology, 215(3), 403–410. https://doi.org/10.1016/S0022-2836(05)80360-2
8. Siguier, P. (2006). ISfinder: the reference centre for bacterial insertion sequences. Nucleic Acids Research, 34(90001), D32–D36. https://doi.org/10.1093/nar/gkj014
9. Filée, J., Siguier, P., & Chandler, M. (2007). Insertion sequence diversity in archaea. Microbiology and Molecular Biology Reviews : MMBR, 71(1), 121—157. https://doi.org/10.1128/mmbr.00031-06

### 
